# (*S*)‐Pyrrolo[1,2‐*a*]pyrazine‐3‐carboxamide Analogs as Dual Akt/JNK Modulators With Antiproliferative Effects in MDA‐MB‐468 Cells

**DOI:** 10.1002/cmdc.70463

**Published:** 2026-08-27

**Authors:** Narges Hosseininasab, Sou Hyun Kim, Ye‐Mi Kwon, Jungjin Park, Chanhyun Jung, Kwanghee Lee, Beom‐Jun Wi, Su‐Jin Yoo, Soonsil Hyun, Young‐Suk Jung, Jae‐Hwan Kwak

**Affiliations:** ^1^ College of Pharmacy Chungbuk National University Cheongju Chungbuk Republic of Korea; ^2^ College of Pharmacy Research Institute for Drug Development Pusan National University Busan Republic of Korea; ^3^ Development Center (NDDC) Daegu‐Gyeongbuk Medical Innovation Foundation (KMEDIhub) Daegu Republic of Korea

**Keywords:** cytotoxic activity, JNK phosphorylation, MDA‐MB‐468 cell, pyrrolopyrazine scaffold, triple‐negative breast cancer

## Abstract

Triple‐negative breast cancer (TNBC) remains a major clinical challenge due to the absence of effective targeted therapies. In this study, thirty analogs of (*S*)‐1‐oxo‐1,2,3,4‐tetrahydropyrrolo[1,2‐*a*]pyrazine‐3‐carboxamide (**A1**–**F5**) were designed, synthesized, and evaluated for their cytotoxic activity against hormone receptor‐positive breast cancer (MCF‐7) and EGFR‐overexpressing TNBC (MDA‐MB‐468) cell lines. Notably, compounds **B1** and **B3** exhibited potent growth inhibition toward MDA‐MB‐468 cells, with GI_50_ values of 2.8 and 4.7 µM, respectively, surpassing the efficacy of the reference compound (**Ref 2**, GI_50_: 7.6 µM) and gefitinib (GI_50_: 16.5 µM). Flow cytometric analysis demonstrated significant apoptosis induction by compounds **B1** and **B3**, with rates of 42.8% and 42.2%, respectively. Mechanistic investigations revealed that compounds **B1** and **B3** selectively inhibited Akt phosphorylation and enhanced JNK phosphorylation, suggesting a dual modulation of survival and apoptotic pathways. These findings support the development of *N2*‐benzyl‐(*S*)‐1‐oxo‐1,2,3,4‐tetrahydropyrrolo[1,2‐*a*]pyrazine‐3‐carboxamide analogs, particularly compound **B1**, as selective therapeutic candidates for triple‐negative breast cancer.

## Graphical abstract

Among 30 synthesized pyrrolo[1,2‐*a*]pyrazine analogs, compounds **B1** and **B3** showed strong and selective cytotoxicity toward TNBC cells, inducing apoptosis through Akt suppression and JNK activation.

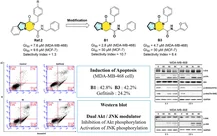

## 1 Introduction

According to recent global estimates (Global Burden of Disease (GBD) 2023; World Health Organization (WHO) 2024), cancer remains the second leading cause of death globally, accounting for 10.4 million deaths and 18.5 million new cases in 2023. By 2050, the global burden will be expected to increase significantly [1]. Tumors develop as a result of cells dividing uncontrollably and proliferating rapidly, while cancer cells have the potential to spread to different parts of the body. Ultimately, this process disrupts the natural function of organs [2]. Breast cancer ranks as the most prevalent neoplasm and the second leading cause of cancer‐related deaths among women [3]. Compared to other cancers, breast cancer treatment results in the largest economic loss [4], highlighting the significance of creating novel treatment approaches for this disease.

There are currently four main subtypes of breast cancer based on the expression of the hormone receptor (HR) and human epidermal growth factor receptor‐2 (HER2): HR^+^/HER2^−^, HR^+^/HER2^+^, HR^−^/HER2^+^, and triple‐negative breast cancer (TNBC), which lacks both HER2 and HR receptor. For HR^+^ cases, hormone therapies such as aromatase inhibitors and estrogen receptor (ER) antagonists are employed, while for HER2^+^ breast cancer cases, HER2‐targeted therapies like pertuzumab and trastuzumab are utilized [5]. However, TNBC, characterized by the absence of these therapeutic targets, emerges as the most aggressive and challenging subtype, requiring innovative approaches [6]. Some factors have been demonstrated to impact the efficacy of anticancer treatment approaches over the years, including multidrug resistance transporter (P‐glycoprotein) and human epidermal growth factor receptor (EGFR) [7].

EGFR is a transmembrane RTK found in the plasma membrane, nuclear membrane, and other cellular components [8]. In some tumors, such as TNBC (52%–54%), lung cancer (40%), glioblastoma (50%), and head and neck cancers (80%–90%), EGFR is overexpressed, whereas it is almost undetectable in the corresponding normal organs [9]. EGFR has two main functions: it increases DNA replication and repair through BRCA1, and it stimulates cell proliferation signaling by phosphorylating phosphatidylinositol 3‐kinase (PI3K) [9]. Altered expressions and mutations of EGFR are frequently observed in TNBC, underscoring the potential for exploiting EGFR as a molecular target in the creation of more efficient treatment strategies [10, 11]. EGFR‐targeted therapies, including small molecule tyrosine kinase inhibitors (TKI) or anti‐EGFR monoclonal antibodies (MAbs) have the capacity to suppress cell proliferation and trigger apoptosis through the downregulation of downstream signaling cascades [12, 13].

Multiple molecular pathways, notably the phosphoinositide 3‐kinase (PI3K)/protein kinase B (AKT)/mechanistic target of rapamycin (mTOR) signaling pathway, are activated in TNBC. This pathway serves as a critical survival and resistance mechanism in TNBC and presents a promising molecular target for TNBC treatment [14]. The pathway connects receptor tyrosine kinase (RTK) signaling to the regulation of cell growth and survival. Hyperactivation of this pathway can lead to abnormal cell differentiation and autophagy, increased cell proliferation, inhibition of apoptosis, tumor formation, and metastasis [15]. In a prior investigation, a combined approach involving inhibitors targeting the PI3K/AKT/mTOR pathway alongside an EGFR‐TK inhibitor was employed to enhance therapeutic efficacy synergistically against TNBC with resistance [16, 17].

Two well‐characterized human breast cancer cell lines, MDA‐MB‐231 and MCF‐7, have different rates of proliferation, metastasis, and response to treatment. MCF‐7 cells are treatable with hormone therapy because they are nonmetastatic. In contrast, MDA‐MB‐231 cells, with their elevated proliferation rate, tend to metastasize and display resistance to hormone therapy, chemotherapy, and radiotherapy [18]. It is interesting to note that MDA‐MB‐468 cells are basal‐like breast carcinoma cells characterized by EGFR overexpression, EGFR wild‐type status, p53 mutation, and PTEN deletion [19]. Because PTEN is deleted in MDA‐MB‐468 cells, the PI3K/AKT/mTOR signaling pathway becomes hyperactive, promoting increased cell growth and survival and making MDA‐MB‐468 cells more resistant to EGFR‐TK inhibitors [20, 21, 22]. Therefore, therapeutic strategies are being developed to directly target PTEN‐deficient cancers or to exploit the synergistic effects of EGFR‐TK inhibitors [23].

Heterocycles, defined as cyclic compounds containing one or more heteroatoms, represent one of the most significant classes of organic compounds in medicinal chemistry. Many heterocycles are essential for life, as they play crucial roles in cellular metabolism [24].

**FIGURE 1:**
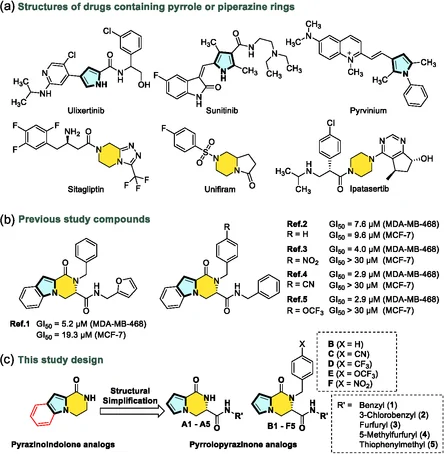
(a) Structures of representative drugs containing pyrrole and piperazine rings; (b) reported compounds active against EGFR‐TKI‐resistant cancer cell lines; and (c) designed compounds obtained through structural simplification of the parent scaffold.

Nitrogen‐containing heterocycles hold a unique position within the class of heterocyclic compounds, serving as widely used structural frameworks in pharmaceuticals [25]. Pyrrole, a five‐membered nitrogen heterocycle, and piperazine, a six‐membered nitrogen‐containing heterocycle, are both of great significance in medicinal chemistry due to their wide‐ranging pharmacological potential [26, 27]. Piperazine derivatives exhibit diverse biological activities, including anticancer [28], antidepressant [29], antibacterial [30], and anticonvulsant [31] effects. Similarly, pyrrole‐based compounds, which occur in both natural and synthetic sources, have gained considerable attention as valuable scaffolds in drug discovery, particularly for their notable anticancer activity [25]. As shown in Figure 1, pyrrole and piperazine rings are key structural motifs in various clinically important drugs.

In our previous efforts, we assessed EGFR‐TK inhibitor resistance using pyrazinoindolone scaffold against two cell lines: MBA‐MB‐468 cell line, which overexpresses EGFR in TNBC, and MCF‐7 cell line, which represents a hormone‐receptor‐positive subtype of breast cancer [23, 32]. Among the pyrazinoindolone analogs, compounds (**Ref 1**–**5**) demonstrated potent activity against MDA‐MB‐468 cells (Figure 1b). Structural simplification of the pyrazinoindolone scaffold was undertaken with two primary objectives. First, elimination of the benzo‐fused indole moiety reduces both molecular weight and lipophilicity, which may enhance aqueous solubility and overall drug‐like characteristics compared to the parent framework. Second, substitution of the indole ring with a smaller pyrrole unit streamlines the synthetic route while preserving the core lactam architecture and the key *N*2 and C3 pharmacophoric elements, namely the *N*2‐benzyl and C3‐carboxamide groups, previously identified as critical for biological activity. Based on this, a pyrrolopyrazine scaffold was designed as a simplified and synthetically accessible analog of the original pyrazinoindolone, retaining the essential structural features associated with antiproliferative effects. In connection with our ongoing interest in developing specific therapeutic agents for patients with cancers resistant to EGFR‐TK inhibitors, this study introduces pyrrolo[1,2‐*a*]pyrazine‐3‐carboxamide derivatives as potential anticancer agents targeting PTEN‐deficient and EGFR‐overexpressing carcinoma cells. Biological evaluations of synthesized compounds were carried out through various methods, including cytotoxicity assays, flow cytometric analysis, combinational treatment with gefitinib, and western blot‐based phosphorylation signaling analysis.

## 2 Results and Discussion

### 2.1 Chemistry

**SCHEME 1:**
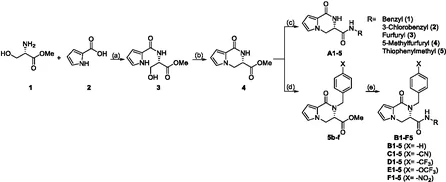
General methods for preparing (*S*)‐1‐oxo‐1,2,3,4‐tetrahydropyrrolo[1,2‐*a*]pyrazine‐3‐carboxamide analogs. Reagents and conditions: (a) EDC, HOBt, Et_3_N, CH_2_Cl_2_, 0 °C to r.t., 17 h, 88%; (b) DIAD, PPh_3_, THF, r.t., 18 h, 48%; (c) arylmethyl amine, MeOH, reflux, 18 h, 14%–75%; (d) substituted benzyl bromide, TBAI, Cs_2_CO_3_, CH_2_Cl_2_, r.t., 12 h, 35%–67%; and (e) amine, MeOH, reflux, 18 h, 29%–86%.

The synthetic procedure for (*S*)‐1‐oxo‐1,2,3,4‐tetrahydropyrrolo[1,2‐*a*]pyrazine‐3‐carboxamide analogs (**A1**–**F5**) is outlined in Scheme 1, based on previously reported studies [23, 32]. The synthesis involves EDC (1‐ethyl‐3‐[3‐dimethylaminopropyl]carbodiimide)/HOBt (1‐hydroxybenzotriazole) mediated amide coupling, followed by Mitsunobu reaction, alkylation, and amidation. Starting with commercially available *L*‐serine methyl ester (**1**) and pyrrole‐2‐carboxylic acid (**2**), methyl (1*H*‐pyrrole‐2‐carbonyl)‐*L*‐serinate (**3**) was produced in 88% yield through an EDC/HOBt coupling reaction. Then, an intramolecular Mitsunobu reaction was performed on compound **3** using diisopropyl azodicarboxylate (DIAD) and triphenylphosphine in tetrahydrofuran to form intermediate **4**, which includes the piperazinone ring. The introduction of an intramolecular Mitsunobu reaction to form the piperazinone ring preserves the stereochemistry of the starting material (**1**). Compounds **A1**–**A5** were obtained in yields ranging from 14% to 75% through direct transamidation of methyl (*S*)‐1‐oxo‐1,2,3,4‐tetrahydropyrrolo[1,2‐*a*]pyrazine‐3‐carboxylate (**4**) with arylmethyl amines. Benzylation of methyl (*S*)‐1‐oxo‐1,2,3,4‐tetrahydropyrrolo[1,2‐*a*]pyrazine‐3‐carboxylate (**4**) using benzyl bromide and its analogs with para‐substituents like CN, CF_3_, OCF_3_, or NO_2_, followed by transamidation with arylmethyl amines, produced compounds **B1**–**F5**. All target compounds and intermediates were characterized using mass spectrometry (LRMS or HRMS), ^1^H NMR, and ^13^C NMR (Supporting Information).

### 2.2 Antiproliferative Activity Against MCF‐7 and MDA‐MB‐468 Cell Lines

A total of thirty analogs of (*S*)‐1‐oxo‐1,2,3,4‐tetrahydropyrrolo[1,2‐*a*]pyrazine‐3‐carboxamide were designed, synthesized, and evaluated for cytotoxic activity against the HR‐positive breast cancer cell line (MCF‐7) and the EGFR‐overexpressed TNBC cell line (MDA‐MB‐468) using a dose‐dependent MTT (3‐(4,5‐dimethylthiazol‐2‐yl)−2,5‐diphenyltetrazolium bromide) assay, as illustrated in Table 1. The compounds were categorized into six groups (**A** to **F**) based on substituents on the *N*2‐position of the (*S*)‐1‐oxo‐1,2,3,4‐tetrahydropyrrolo[1,2‐*a*]pyrazine ring, to facilitate the comparison of their structure–activity relationship (SAR).

**TABLE 1:** Cell viability at 10 and 30 µM, GI_50_ value, and selectivity index (SI) of (*S*)‐1‐oxo‐1,2,3,4‐tetrahydropyrrolo[1,2‐*a*]pyrazine‐3‐carboxamide analogs (**A1**–**F5**) against the MCF‐7 and MDA‐MB‐468 cell lines.

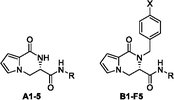							
Compounds	R	X	% Cell viability at 30 µM		GI_50_ value^a^, µM		
			MCF‐7	MDA‐MB‐468	MCF‐7	MDA‐MB‐468	SI
**Ctrl**	—	—	100 ± 2.8	100 ± 3.2	—	—	—
**A1**	Benzyl	—	89.8 ± 1.6	92.2 ± 1.4	>30	>30	—
**A2**	3‐Chlorobenzyl	—	89.4 ± 4.6	87.7 ± 5.4	>30	>30	—
**A3**	Furfuryl	—	95.0 ± 2.7	92.1 ± 4.6	>30	>30	—
**A4**	5‐Methylfurfuryl	—	86.3 ± 3.2	81.1 ± 9.8	>30	>30	—
**A5**	Thiophenylmethyl	—	90.6 ± 8.0	93.4 ± 5.0	>30	>30	—
**B1**	Benzyl	H	62.6 ± 2.4	19.0 ± 0.8	>30	2.8	>10.7
**B2**	3‐Chlorobenzyl	H	72.6 ± 2.5	22.5 ± 1.3	>30	7.1	>4.2
**B3**	Furfuryl	H	61.4 ± 5.1	19.4 ± 0.2	>30	4.7	>6.4
**B4**	5‐Methylfurfuryl	H	78.9 ± 2.6	24.1 ± 1.0	>30	8.4	>3.6
**B5**	Thiophenylmethyl	H	56.5 ± 2.3	17.1 ± 0.5	>30	6.0	>5.0
**C1**	Benzyl	CN	80.3 ± 4.0	27.4 ± 0.9	>30	19.5	>1.5
**C2**	3‐Chlorobenzyl	CN	71.6 ± 3.3	40.9 ± 15.0	>30	25	>1.2
**C3**	Furfuryl	CN	85.2 ± 1.3	45.0 ± 4.7	>30	27.6	>1.1
**C4**	5‐Methylfurfuryl	CN	92.3 ± 3.0	65.2 ± 7.5	>30	>30	—
**C5**	Thiophenylmethyl	CN	77.8 ± 5.2	36.4 ± 1.3	>30	23.2	>1.3
**D1**	Benzyl	CF_3_	44.1 ± 4.0	10.0 ± 1.2	25.6	15.5	1.7
**D2**	3‐Chlorobenzyl	CF_3_	45.7 ± 2.1	21.1 ± 1.1	27.3	16.4	1.7
**D3**	Furfuryl	CF_3_	75.5 ± 3.2	35.5 ± 1.8	>30	23.7	>1.3
**D4**	5‐Methylfurfuryl	CF_3_	64.6 ± 2.9	33.1 ± 3.5	>30	23.1	>1.3
**D5**	Thiophenylmethyl	CF_3_	76.2 ± 1.9	68.2 ± 2.2	>30	>30	—
**E1**	Benzyl	OCF_3_	48.7 ± 2.2	13.1 ± 2.5	28.7	14.5	2.0
**E2**	3‐Chlorobenzyl	OCF_3_	48.6 ± 3.0	32.7 ± 2.7	28.8	21.2	1.4
**E3**	Furfuryl	OCF_3_	70.9 ± 1.0	30.4 ± 4.1	>30	21.3	>1.4
**E4**	5‐Methylfurfuryl	OCF_3_	54.6 ± 3.8	19.0 ± 2.6	>30	25.1	>1.2
**E5**	Thiophenylmethyl	OCF_3_	48.5 ± 6.0	15.2 ± 2.4	28.8	14.8	2.0
**F1**	Benzyl	NO_2_	67.6 ± 4.7	34.3 ± 1.5	>30	21.8	>1.4
**F2**	3‐Chlorobenzyl	NO_2_	77.4 ± 4.4	44.6 ± 3.0	>30	25.6	>1.2
**F3**	Furfuryl	NO_2_	92.9 ± 2.6	92.1 ± 7.6	>30	>30	—
**F4**	5‐Methylfurfuryl	NO_2_	81.4 ± 4.7	42.2 ± 4.4	>30	26.3	>1.1
**F5**	Thiophenylmethyl	NO_2_	76.4 ± 8.5	41.5 ± 2.0	>30	25.2	>1.2
**Ref 2**	—	—	30.7 ± 1.7	20.7 ± 0.8	9.6	7.6	1.26
**Gefitinib**	—	—	79.1 ± 4.9	9.7 ± 1.1	>30	16.5	>1.8

a GI_50_ values are reported as the mean of four experiments and correspond to the agent's concentration that causes a 50% decrease in net cell growth.

In general, they exhibited low activity against MCF‐7 cell lines. Only compounds **D1** (GI_50_: 25.6 µM) and **D2** (GI_50_: 27.3 µM), featuring a 4‐trifluoromethyl benzyl group, and compounds **E1** (GI_50_: 28.7 µM), **E2** (GI_50_: 28.8 µM), and **E5** (GI_50_: 28.8 µM), with a 4‐trifluoromethoxy benzyl group, displayed GI_50_ values below 30 µM. However, their antiproliferative activity was lower than that of the reference compound (**Ref 2**, GI_50_: 9.6 µM).

In terms of activity against MDA‐MB‐468 cells, compounds **A1**–**A5** without the *N*2‐benzyl group exhibited very low inhibitory activity. Conversely, compounds **B1**–**F5**, with the exception of compound **F3**, demonstrated results indicating inhibition of MDA‐MB‐468 cell growth. When comparing based on substituents on the *N*2‐benzyl group of (*S*)‐1‐oxo‐1,2,3,4‐tetrahydropyrrolo[1,2‐*a*]pyrazine‐3‐carboxamide, compounds **B1**–**B5**, with hydrogen instead of substituents (4‐CN, 4‐CF_3_, 4‐OCF_3_, and 4‐NO_2_), exhibited the highest activity. In the B series, compounds **B1** and **B3** showed GI_50_ values of 2.8 and 4.7 µM, respectively, surpassing those of **B2** (GI_50_: 7.1 µM), **B4** (GI_50_: 8.4 µM), and **B5** (GI_50_: 6.0 µM). In the C–F series, five compounds (**E1**, **E5**, **D1**, **D2**, and **C1**) exhibited GI_50_ values of 14.5, 14.8, 15.5, 16.4, and 19.5 µM, respectively, while the remaining compounds ranged from 21.2 to 27.6 µM, except for **C4**, **D5**, and **F3**, which had GI_50_ values over 30 µM. The type of substituent on the 3‐carboxamide of (*S*)‐1‐oxo‐1,2,3,4‐tetrahydropyrrolo[1,2‐*a*]pyrazine analogs showed a slight difference in their antiproliferative activity. Among the benzyl (**A1**–**F1**), 3‐chlorobenzyl (**A2**–**F2**), furfuryl (**A3**–**F3**), 5‐methylfurfuryl (**A4–F4**), and thiophene‐2‐ylmethyl group (**A5**–**F5**), compounds with a benzyl carboxamide group, except for series A which exhibited low activity, demonstrated the strongest GI_50_ values in each series: 2.8 µM (**B1**), 19.5 µM (**C1**), 15.5 µM (**D1**), 14.5 µM (**E1**), and 21.8 µM (**F1**), respectively. Notably, compound **B1** exhibited the highest activity against MDA‐MB‐468 cells, showing activity 2.7‐fold more potent than the reference compound (**Ref 2**), which possesses *N*2‐benzyl and benzyl carboxamide on the tetrahydropyrazino[1,2‐*a*]indole moiety instead of the tetrahydropyrrolo[1,2‐*a*]pyrazine ring. Additionally, it exhibited 5.89‐fold stronger activity than gefitinib, an EGFR‐TK inhibitor.

**FIGURE 2:**
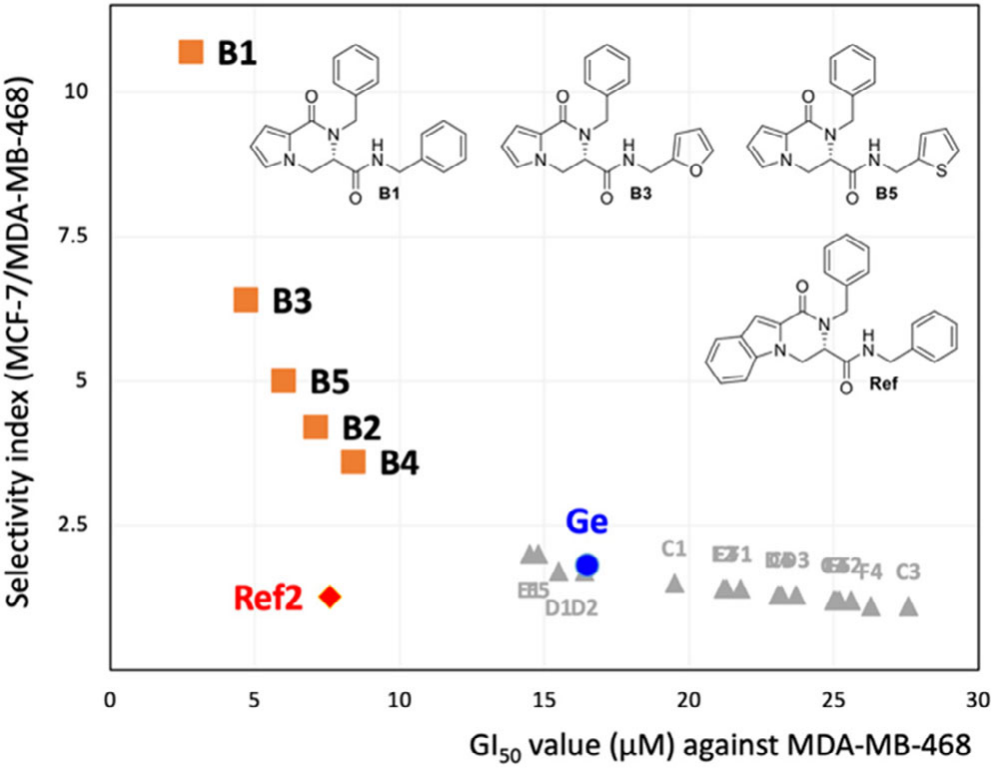
Selectivity index and GI_50_ values of compounds against MCF‐7 and MDA‐MB‐468 cells.

The comparison of antiproliferative activity between MCF‐7 and MDA‐MB‐468 cells suggests that novel compounds may offer the potential to target specific receptors on triple‐negative cancer cells, rather than exerting a generalized chemotherapy effect without selectivity. The Selectivity Index (SI) was calculated based on the GI_50_ values against MCF‐7 and MDA‐MB‐468 cells. The SI value greater than 1 indicates that the activity on MDA‐MB‐468 cells is more potent than on MCF‐7 cells. Compounds, excluding series **A**, **C4**, **D5**, and **F3**, which have low activity, showed SI values greater than 1. The SI values of compounds **Ref 2** and gefitinib are 1.26 and more than 1.8, respectively. Several compounds in series C–F showed comparable SI values, with **E1** and **E5** achieving the highest selectivity in these series (SI = 2.0 each). Compounds **B1**–**B5**, an *N*2‐benzyl‐(*S*)‐1‐oxo‐1,2,3,4‐tetrahydropyrrolo[1,2‐*a*]pyrazine‐3‐carboxamide series, exhibited SI values of more than 3.6. Compound **B1** (SI value: >10.7) demonstrated the highest SI value, being 8.49 and 5.94‐fold more selective than compounds **Ref 2** and gefitinib, respectively (Figure 2).

To summarize, the introduction of substituents into the *N*2‐benzyl ring resulted in modifications in antiproliferative activity and tumor selectivity. A particularly noteworthy observation was a significant improvement observed in series B, which lacked substituents. The enhanced activity observed for Series B, which features an unsubstituted *N*2‐benzyl moiety, can be interpreted through a combination of steric and electronic considerations. The unsubstituted phenyl ring likely achieves an optimal balance between hydrophobic surface area and steric demand, enabling favorable hydrophobic and π–π interactions within the presumed binding pocket without introducing steric strain. In contrast, incorporation of electron‐withdrawing substituents in Series **C**–**F** (4‐CN, 4‐CF_3_, 4‐OCF_3_, and 4‐NO_2_) decreases the electron density of the aromatic system, potentially weakening π‐interactions that contribute to ligand binding. Additionally, the nature of the substituent on the 3‐carboxamide was found to be critical for their antiproliferative activity. Among these substituents, the benzyl amide group exhibited the highest activity. Therefore, compound **B1**, possessing both *N*2‐benzyl and 3‐benzylamide, emerged as the most potent and selective against the MDA‐MB‐468 cell lines. It demonstrated heightened antiproliferative activity, showing greater sensitivity in MDA‐MB‐468 cells compared to MCF‐7 cells. This suggests a potential impact on activated pathways specific to MDA‐MB‐468 cells. Subsequently, additional biological studies were conducted to validate the mechanism of action.

### 2.3 Flow Cytometric Analysis

**FIGURE 3:**
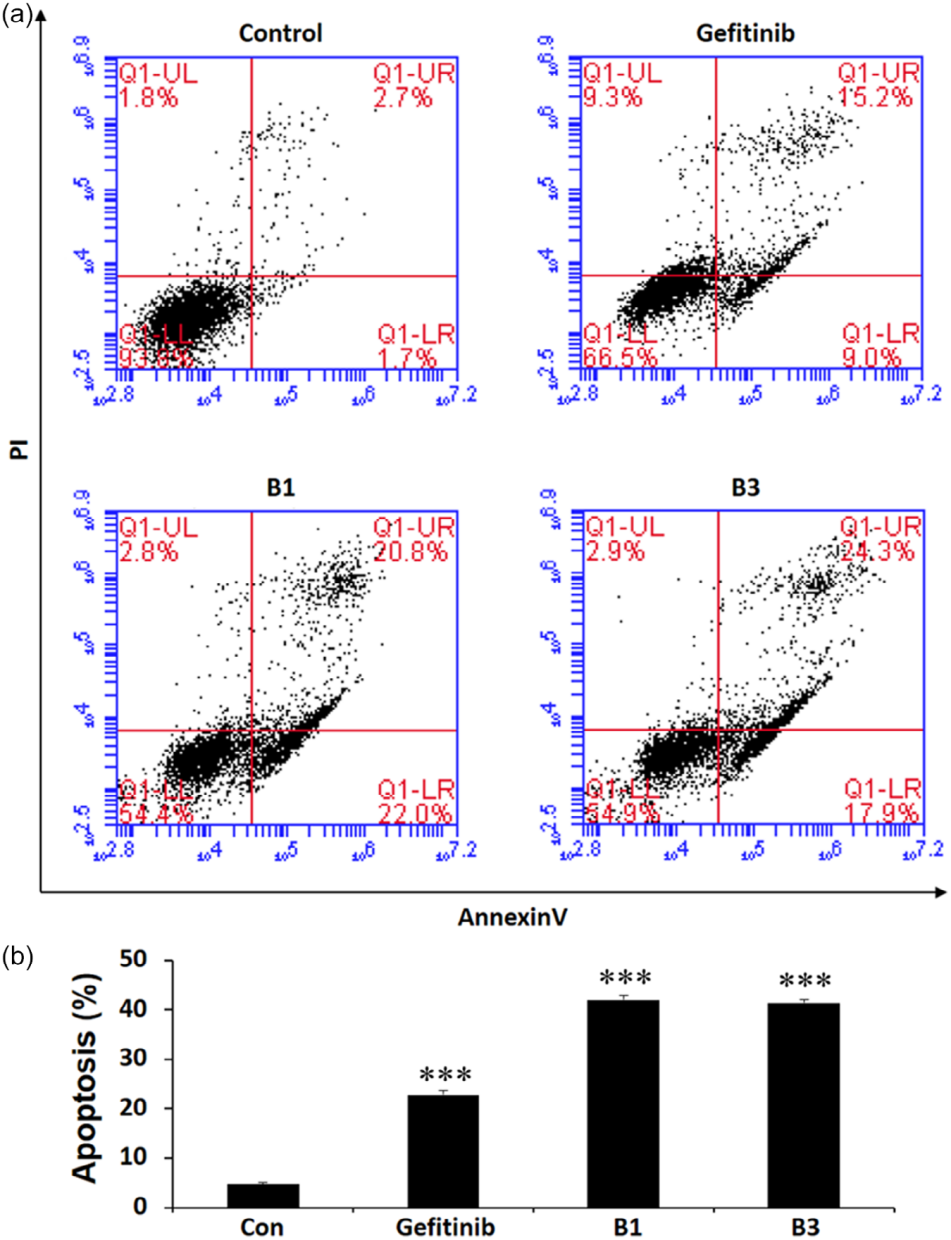
Flow cytometric analysis to determine apoptosis in MDA‐MB‐468 cells. (a) The cells were treated with 10 μM of gefitinib, and compound **B1**, and **B3**, respectively, for 24 h. (b) Fluorescence‐activated cell sorting (FACS) analysis of propidium iodide (PI) uptake and annexin V binding in nonpermeabilized cells (lower‐left, live cells; lower‐right, early apoptotic cells; upper‐right, late apoptotic cells; upper‐left, necrotic cells). The quantification of apoptotic cells from three independent experiments. (***p < 0.001, compared with control cells).

To assess the apoptotic effect on the MDA‐MB‐468 cell line, compounds **B1** and **B3**, which exhibited the highest potency and tumor‐selective activity, underwent comprehensive analysis using flow cytometry with annexin V‐FITC and propidium iodide (PI), as illustrated in Figure 3. The measured rates of apoptosis induction activated by compounds **B1** and **B3** were 42.8% and 42.2%, respectively. These rates were approximately 1.75‐fold more potent than the positive control, gefitinib, which displayed a 24.2% induction rate at a concentration of 10 µM over a 24‐h period. These compelling findings strongly suggest that compounds **B1** and **B3** induce apoptosis through programmed cell death pathways in MDA‐MB‐468 cells.

### 2.4 Effects on Phosphorylation Signaling of Erk, Akt, and JNK

To enhance our understanding of the mechanisms underlying the activity of compounds **B1** and **B3**, we conducted tests to assess and compare their inhibitory effects with gefitinib (**GE**) against MDA‐MB‐468 cells through western blot analysis. Initially, MDA‐MB‐468 cell lines, associated with basal‐like TNBC, exhibited an overexpression of the EGFR. Consequently, our aim was to investigate the impact of compounds **B1** and **B3** on the Ras/Raf/MEK/ERK and PI3K/AKT/mTOR pathways, which constitute the major downstream signaling pathways of EGFR.

**FIGURE 4:**
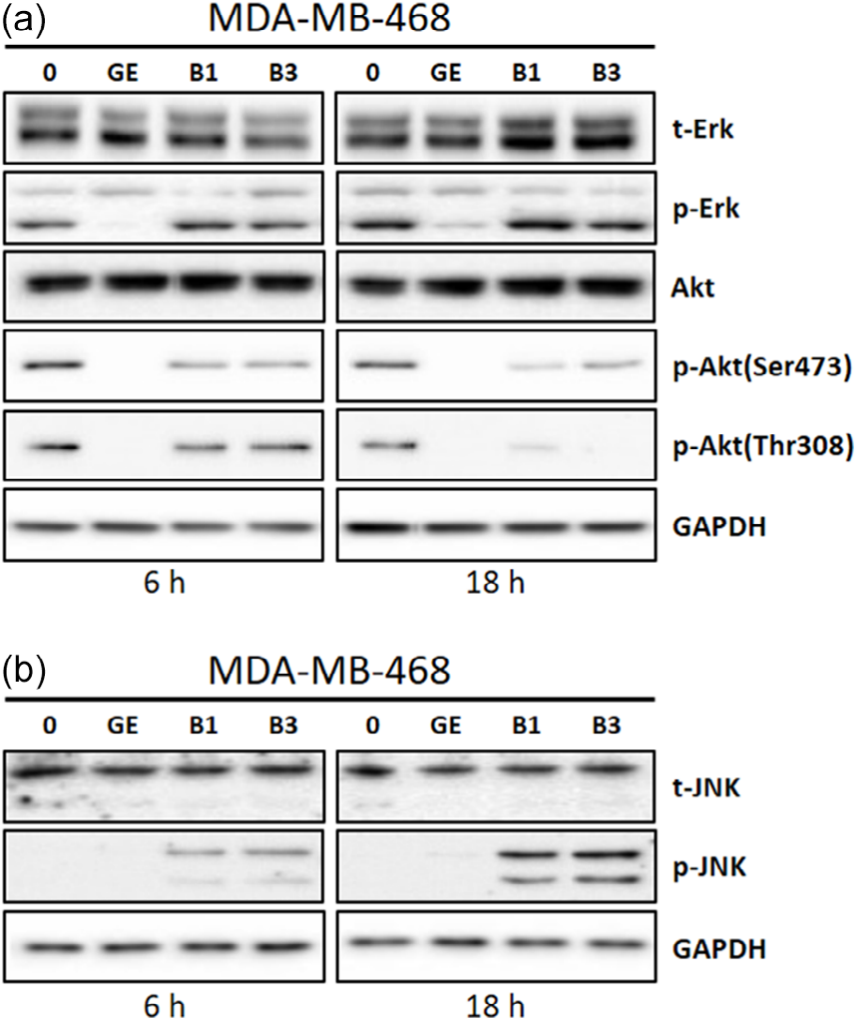
Effects on phosphorylation signaling of Erk, Akt, and JNK in MDA‐MB‐468 cells. Cells were treated with 10 μM of **GE**, compound **B1**, and compound **B3** for 6 and 18 h, respectively. (a) Protein expression of total‐ERK, phospho‐ERK, Akt, phospho‐Akt (Ser^473^), and phospho‐Akt (Thr^308^) was detected by immunoblotting. GAPDH was used as a loading control. (b) Protein expression of total‐JNK and phospho‐JNK was detected by immunoblotting. GAPDH was used as a loading control.

In the western blot analysis after 6 h, gefitinib (**GE**), a well‐known EGFR‐TK inhibitor, significantly reduced phosphorylation levels of both ERK and Akt. However, compounds **B1** and **B3** did not suppress ERK phosphorylation but exhibited a slight inhibition of Akt phosphorylation at residues Ser^473^ and Thr^308^. In an 18‐h analysis, gefitinib (**GE**) consistently demonstrated inhibitory activity on the ERK and Akt pathways. In the case of the synthesized compounds, they specifically targeted the Akt pathways and exhibited a more potent inhibition of Akt phosphorylation compared to the 6‐h time point (Figure 4a).

However, the basis for its higher activity compared to gefitinib remains unclear, and we therefore sought to clarify the mechanism by which it suppresses the growth of MDA‐MB‐468 cells. It has been reported that the expression of caspases, key components of the apoptotic machinery, is not uniform among human breast cancer cell lines [33, 34]. In particular, procaspase‐3 is lacking in MCF‐7 cells but highly expressed in MDA‐MB‐468 cells, and this discrepancy influences the survival of the two cell lines when treated with the active compound. Since JNK signaling is known to facilitate caspase‐3 activation and apoptosis in cancer cells, [33, 35] the different levels of procaspase‐3 expression may help to account for the distinct cellular responses to JNK activation. To verify this possibility, we examined the effect of our compounds on JNK phosphorylation and confirmed that they markedly enhanced JNK activity (Figure 4b). Taken together, these findings indicate that the antiproliferative effects of the compounds are at least partly mediated by JNK‐dependent caspase activation.

### 2.5 Combinational Treatment of Compounds B1 and B3 With Gefitinib

**FIGURE 5:**
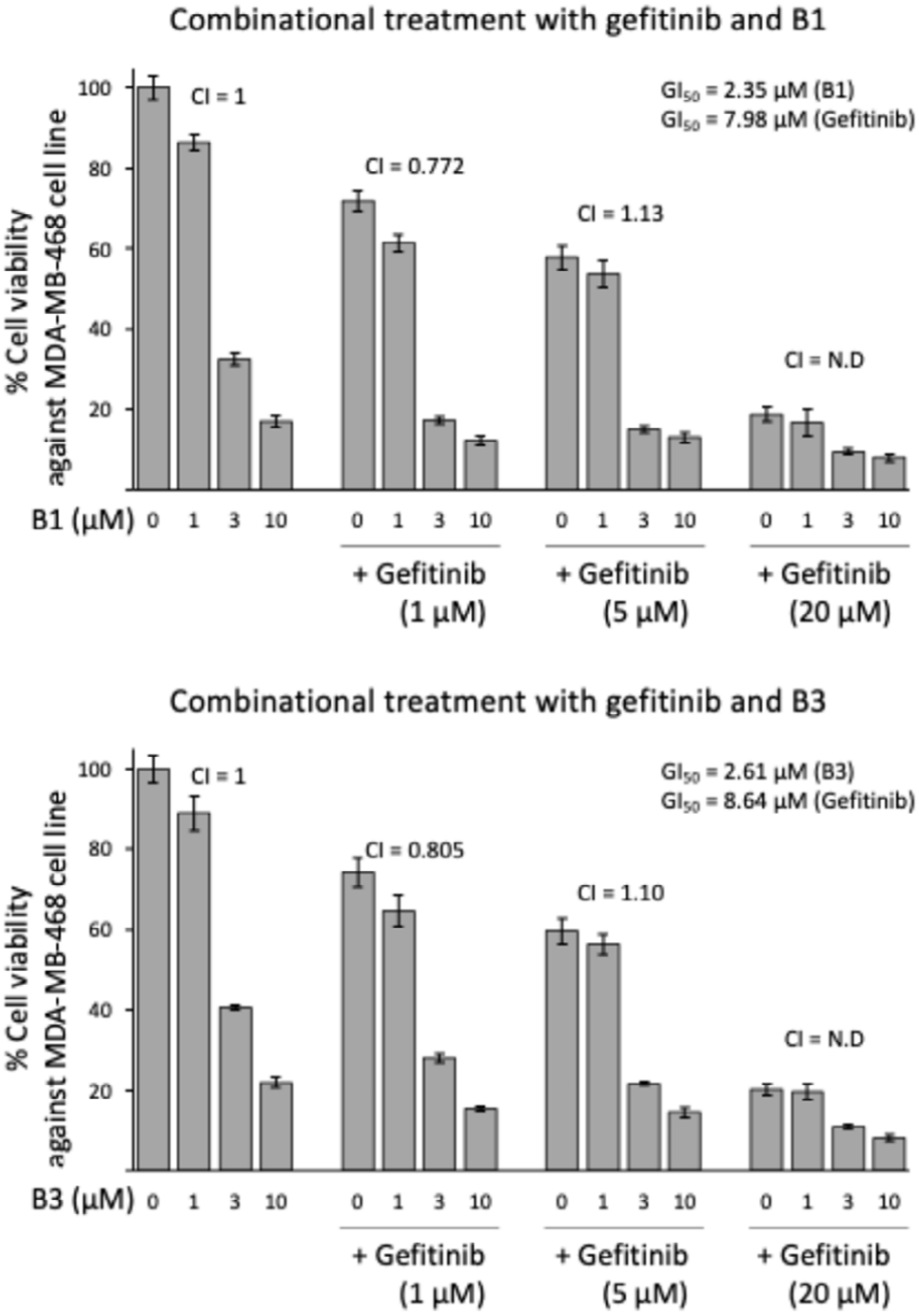
Combinational treatment with gefitinib and compounds (**B1** and **B3**) against MDA‐MB‐468 cell viability and CI value. N.D., not detected.

To explore potential synergistic effects, combinational treatments of gefitinib with compounds **B1** and **B3** were evaluated against MDA‐MB‐468 cells (Figure 5). The combination index (CI) was calculated using the Chou‐Talalay method, where CI values < 1 indicate synergism, CI = 1 indicates an additive effect, and CI > 1 indicates antagonism. Both **B1** and **B3** demonstrated synergistic effects when combined with low dose of gefitinib, as indicated by CI values below 1. These results suggest that combining **B1** or **B3** with EGFR inhibitors could enhance therapeutic efficacy against PTEN‐deficient, EGFR‐overexpressing TNBC.

## 3 Conclusion

To summarize, this study successfully synthesized and evaluated thirty analogs of (*S*)‐1‐oxo‐1,2,3,4‐tetrahydropyrrolo[1,2‐*a*]pyrazine‐3‐carboxamide, demonstrating their cytotoxic potential against breast cancer cell lines MCF‐7 and MDA‐MB‐468. While the analogs generally exhibited low activity against MCF‐7 cells, specific compounds (**D1**, **D2**, **E1**, **E2**, and **E5**) showed moderate activity. In contrast, some compounds (notably **B1** and **B3**) showed significant inhibitory activity against the MDA‐MB‐468 cell line, surpassing the reference compound in potency and selectivity. Compound **B1** emerged as the most effective, with a GI_50_ value of 2.8 µM and an SI value greater than 10.7, indicating high selectivity for MDA‐MB‐468 cells over MCF‐7 cells. Apoptosis studies confirmed that compounds **B1** and **B3** induce significant programmed cell death in MDA‐MB‐468 cells, with apoptosis rates significantly higher than those induced by the positive control, gefitinib. In addition, mechanistic studies indicated that these compounds selectively inhibited Akt phosphorylation while promoting JNK activation. These findings underscore the potential of *N*2‐benzyl‐substituted (*S*)‐1‐oxo‐1,2,3,4‐tetrahydropyrrolo[1,2‐*a*]pyrazine‐3‐carboxamide analogs as selective and potent therapeutic agents against TNBC.

## 4 Experimental Section

### 4.1 Materials and Methods

^1^H NMR spectra were recorded on a JEOL JNM‐EX400 (400 MHz, JEOL, Tokyo, Japan). Chemical shifts are reported in ppm from tetramethyl silane (TMS) with the solvent resonance resulting from incomplete deuteration as the internal reference (CDCl_3_: 7.26 ppm). Data are reported as follows: chemical shift, multiplicity (s = singlet, d = doublet, t = triplet, q = quartet, br = broad, m = multiplet, dd = doublet of doublets, dt = doublet of triplets), coupling constants (Hz), and number of protons. ^13^C NMR spectra were recorded on a JEOL JNM‐EX400 (100 MHz) with complete proton decoupling. Chemical shifts are reported in ppm from tetramethyl silane (TMS) with the solvent used as the internal reference (CDCl_3_: 77.16 ppm). Low‐resolution and high‐resolution mass data were recorded on a Dalton 2000 (Biotage, Uppsala, Sweden) in APCI positive mode or an ESI‐MS Agilent 6530 Accurate‐Mass Quadrupole Time‐of‐Flight (Q‐TOF) spectrometer (Agilent, Santa Clara, CA, USA) in ESI positive mode. Analytical thin‐layer chromatography was performed on Kieselgel 60 F254 silica gel plates (Merck). Isolations by column chromatography were performed using silica gel 60 (230–400 mesh, Merck) and medium pressure liquid chromatography (MPLC: Biotage Isolera, Uppsala, Sweden).

### 4.2 Synthesis of Methyl (1*H*‐Pyrrole‐2‐Carbonyl)‐*L*‐Serinate (3)

To an anhydrous CH_2_Cl_2_ solution of pyrrole‐2‐carboxylic acid (**2**) (300 mg, 2.70 mmol), HOBt (365 mg, 2.70 mmol), and *L*‐serine methyl ester (**1**) (504 mg, 3.24 mmol), EDC (480 mg, 2.70 mmol) was added, and the mixture was cooled to 0 °C. Triethylamine was added dropwise, and then the reaction mixture was warmed to ambient temperature. After stirring for 17 h, the reaction mixture was concentrated under reduced pressure. The crude product was purified by flash column chromatography on silica gel (EtOAc/Hexane = 2/1) to yield compound **3** with an 88% yield. ^1^H NMR (400 MHz, CDCl_3_) *δ* 9.94 (s, 1H, NH), 7.08 (d, *J* = 7.5 Hz, 1H, NH), 6.92–6.89 (m, 1H), 6.73–6.70 (m, 1H), 6.22–6.19 (m, 1H), 4.82 (dt, *J* = 7.5, 3.7 Hz, 1H), 4.01 (ABq, *J* = 11.4, 3.6 Hz, 2H), 3.77 (s, 3H); ^13^C NMR (100 MHz, CDCl_3_) *δ* 171.4, 161.6, 125.0, 122.6, 110.9, 110.1, 63.5, 54.8, 53.0; LRMS (APCI) *m*/*z* 213.2 [M + H]^+^.

### 4.3 Synthesis of Methyl (*S*)‐1‐Oxo‐1,2,3,4‐Tetrahydropyrrolo[1,2‐*a*]pyrazine‐3‐Carboxylate (4)

A mixture of methyl (1*H*‐pyrrole‐2‐carbonyl)‐*L*‐serinate (**3**) (503 mg, 2.37 mmol), triphenylphosphine (808 mg, 3.08 mmol), DIAD (1.62 mL, 3.08 mmol), and anhydrous THF (60 mL) was stirred. After stirring for 18 h, the solvent evaporated, and purification via flash column chromatography (EtOAc/Hexane = 1/1) provided compound **4** with a yield of 48%. ^1^H NMR (400 MHz, CDCl_3_) *δ* 9.79 (s, 1H, NH), 6.95–6.93 (m, 1H), 6.80–6.78 (m, 1H), 6.25–6.23 (m, 1H), 4.89 (dd, *J* = 10.4, 7.7 Hz, 1H), 4.63 (dd, *J* = 8.6, 7.7 Hz, 1H), 4.55 (dd, *J* = 10.4, 8.6 Hz, 1H), 3.79 (s, 3H); ^13^C NMR (100 MHz, CDCl_3_) *δ* 171.9, 160.9, 122.8, 119.3, 113.9, 110.2, 69.6, 68.1, 52.9; LRMS (APCI) *m*/*z* 195 [M + H]^+^.

### 4.4 General Procedure for (*S*)‐2‐Substituted Benzyl‐1‐Oxo‐1,2,3,4‐Tetrahydropyrrolo[1,2‐*a*]pyrazine‐3‐Carboxylate (5b–5f)

A mixture of methyl (*S*)‐1‐oxo‐1,2,3,4‐tetrahydropyrrolo[1,2‐*a*]pyrazine‐3‐carboxylate (**4**) (1 eq.), Cs_2_CO_3_ (2 eq.) and anhydrous CH_2_Cl_2_ was stirred for 30 min, then substituted benzyl bromide (1.2 eq.) was added. After stirring for 12 h, the solvent evaporated, and purification via flash column chromatography provided compounds **5b**–**5f**.

**Methyl (*S*)‐2‐Benzyl‐1‐Oxo‐1,2,3,4‐Tetrahydropyrrolo[1,2‐*a*]pyrazine‐3‐Carboxylate** (**5b**). Compound **5b** was synthesized using the synthetic procedure outlined above, yield: 57%. ^1^H NMR (400 MHz, CDCl_3_) *δ* 7.31–7.26 (m, 2H), 7.26–7.21 (m, 1H), 7.12 (d, *J* = 6.6 Hz, 2H), 6.86–6.83 (m, 2H), 6.18 (dd, *J* = 3.9, 2.6 Hz, 1H), 5.67 (s, 2H), 4.86 (dd, *J* = 10.3, 7.3 Hz, 1H), 4.50 (dd, *J* = 8.6, 7.3 Hz, 1H), 4.40 (dd, *J* = 10.3, 8.6 Hz, 1H), 3.73 (s, 3H); ^13^C NMR (100 MHz, CDCl_3_) *δ* 172.1, 160.3, 138.5, 128.6, 127.9, 127.43, 127.38, 119.9, 116.4, 108.8, 68.8, 68.3, 52.6, 52.1; LRMS (APCI) *m*/*z* 285 [M + H]^+^.

**Methyl (*S*)‐2‐(4‐Cyanobenzyl)‐1‐Oxo‐1,2,3,4‐Tetrahydropyrrolo[1,2‐*a*]pyrazine‐3‐Carboxylate** (**5c**). Compound **5c** was synthesized using the synthetic procedure outlined above, yield: 48%. ^1^H NMR (400 MHz, CDCl_3_) *δ* 7.56 (d, *J* = 8.5 Hz, 2H), 7.16 (d, *J* = 8.7 Hz, 2H), 6.87–6.85 (m, 2H), 6.23 (dd, *J* = 3.6, 2.9 Hz, 1H), 5.72 (ABq, *J* = 18.0, 16.1 Hz, 2H), 4.81 (dd, *J* = 10.3, 7.3 Hz, 1H), 4.46 (dd, *J* = 8.6, 7.3 Hz, 1H), 4.38 (dd, *J* = 10.3, 8.6 Hz, 1H), 3.70 (s, 3H); ^13^C NMR (100 MHz, CDCl_3_) *δ* 171.9, 160.0, 144.2, 132.4, 128.0, 127.7, 119.9, 118.9, 116.8, 111.2, 109.4, 68.8, 68.4, 52.7, 51.7; LRMS (APCI) *m*/*z* 310 [M + H]^+^.

**Methyl (*S*)‐1‐Oxo‐2‐(4‐(Trifluoromethyl)benzyl)−1,2,3,4‐Tetrahydropyrrolo[1,2‐*a*]pyrazine‐3‐Carboxylate** (**5d**). Compound **5d** was synthesized using the synthetic procedure outlined above, yield: 67%. ^1^H NMR (400 MHz, CDCl_3_) *δ* 7.53 (d, *J* = 8.1 Hz, 2H), 7.19 (d, *J* = 8.1 Hz, 2H), 6.87–6.85 (m, 2H), 6.22 (dd, *J* = 3.7, 2.8 Hz, 1H), 5.74 (ABq, *J* = 22.7, 15.7 Hz, 2H), 4.83 (dd, *J* = 10.3, 7.2 Hz, 1H), 4.48 (dd, *J* = 8.6, 7.3 Hz, 1H), 4.39 (dd, *J* = 10.3, 8.6 Hz, 1H), 3.69 (s, 3H); ^13^C NMR (100 MHz, CDCl_3_) *δ* 172.0, 160.1, 142.8, 129.9 (*J*_CF_ = 31.7 Hz), 128.0, 127.4, 125.5 (*J*_CF_ = 3.8 Hz), 124.2 (*J*_CF_ = 273 Hz), 120.0, 116.7, 109.2, 68.8, 68.4, 52.6, 51.7; LRMS (APCI) *m*/*z* 353 [M + H]^+^.

**Methyl (*S*)‐1‐Oxo‐2‐(4‐(Trifluoromethoxy)benzyl)‐1,2,3,4‐Tetrahydropyrrolo[1,2‐*a*]pyrazine‐3‐Carboxylate** (**5e**). Compound **5e** was synthesized using the synthetic procedure outlined above, yield: 42%. ^1^H NMR (400 MHz, CDCl_3_) *δ* 7.17–7.11 (m, 4H), 6.86–6.85 (m, 2H), 6.20 (dd, *J* = 3.5, 2.9 Hz, 1H), 5.69 (ABq, *J* = 28.1, 15.4 Hz, 2H), 4.86 (dd, *J* = 10.3, 7.3 Hz, 1H), 4.49 (dd, *J* = 8.5, 7.3 Hz, 1H), 4.40 (dd, *J* = 10.3, 8.6 Hz, 1H), 3.72 (s, 3H); ^13^C NMR (100 MHz, CDCl_3_) *δ* 172.0, 160.1, 148.5 (*J*_CF_ = 1 Hz), 137.4, 128.7, 127.9, 121.1, 120.6 (*J*_CF_ = 257 Hz), 119.9, 116.6, 109.1, 68.9, 68.3, 52.6, 51.3; LRMS (APCI) *m*/*z* 369 [M + H]^+^.

**Methyl (*S*)‐2‐(4‐Nitrobenzyl)‐1‐Oxo‐1,2,3,4‐Tetrahydropyrrolo[1,2‐*a*]pyrazine‐3‐Carboxylate** (**5f**). Compound **5f** was synthesized using the synthetic procedure outlined above, yield: 35%. ^1^H NMR (400 MHz, CDCl_3_) *δ* 8.13 (d, *J* = 8.8 Hz, 2H), 7.22 (d, *J* = 9.0 Hz, 2H), 6.89–6.86 (m, 2H), 6.25 (dd, *J* = 3.8, 2.7 Hz, 1H), 5.77 (ABq, *J* = 18.6, 16.1 Hz, 2H), 4.81 (dd, *J* = 10.3, 7.3 Hz, 1H), 4.46 (dd, *J* = 8.6, 7.3 Hz, 1H), 4.39 (dd, *J* = 10.3, 8.6 Hz, 1H), 3.70 (s, 3H); ^13^C NMR (100 MHz, CDCl_3_) *δ* 171.9, 160.0, 147.3, 146.2, 128.0, 127.8, 123.9, 119.9, 116.9, 109.5, 68.8, 68.4, 52.6, 51.6; LRMS (APCI) *m*/*z* 330 [M + H]^+^.

### 4.5 General Procedure of (*S*)‐2‐Substituted‐1‐Oxo‐1,2,3,4‐Tetrahydropyrrolo[1,2‐*a*]pyrazine‐3‐Carboxamide (A1–F5)

A mixture of methyl (*S*)‐1‐oxo‐1,2,3,4‐tetrahydropyrrolo[1,2‐*a*]pyrazine‐3‐carboxylate (**4**) or (*S*)‐2‐substitutedbenzyl‐1‐oxo‐1,2,3,4‐tetrahydropyrrolo[1,2‐*a*]pyrazine‐3‐carboxylate (**5b**–**5f**) (1 eq.) and absolute MeOH was stirred. An arylmethylamine (5 eq.) was added, and the mixture was heated at reflux conditions. After stirring for 18 h, the solvent evaporated, and purification via flash column chromatography provided compounds **A1**–**F5**.

**(*S*)‐*N*‐Benzyl‐1‐Oxo‐1,2,3,4‐Tetrahydropyrrolo[1,2‐*a*]pyrazine‐3‐Carboxamide** (**A1**). Compound **A1** was synthesized using the synthetic procedure outlined above, yield: 14%. ^1^H NMR (400 MHz, CDCl_3_) *δ* 9.34 (brs, 1H, NH), 7.34–7.23 (m, 5H), 7.00 (brs, 1H, NH), 6.93–6.92 (m, 1H), 6.81–6.79 (m, 1H), 6.28–6.26 (m, 1H), 4.81 (dd, *J* = 10.7, 8.1 Hz, 1H), 4.65 (dd, *J* = 10.7, 8.7 Hz, 1H), 4.59–4.50 (m, 2H), 4.33 (dd, *J* = 14.8, 5.4 Hz, 1H); ^13^C NMR (100 MHz, CDCl_3_) *δ* 171.9, 160.8, 137.9, 128.9, 127.9, 127.7, 122.7, 119.4, 114.0, 110.6, 70.7, 68.7, 43.3; HRMS (ESI‐QTOF) *m*/*z* calcd for C_15_H_15_N_3_O_2_^+^ [M + H]^+^ 270.1237, found 270.1234.

**(*S*)‐*N*‐(3‐Chlorobenzyl)‐1‐Oxo‐1,2,3,4‐Tetrahydropyrrolo[1,2‐*a*]pyrazine‐3‐Carboxamide** (**A2**). Compound **A2** was synthesized using the synthetic procedure outlined above, yield: 75%. ^1^H NMR (400 MHz, CDCl_3_) *δ* 9.22 (brs, 1H, NH), 7.25–7.22 (m, 3H), 7.15–7.11 (m, 1H), 7.04 (brs, 1H, NH), 6.94–6.92 (m, 1H), 6.83–6.80 (m, 1H), 6.29–6.26 (m, 1H), 4.82 (dd, *J* = 10.7, 8.1 Hz, 1H), 4.66 (dd, *J* = 10.7, 8.7 Hz, 1H), 4.58–4.51 (m, 2H), 4.29 (dd, *J* = 15.0, 5.5 Hz, 1H); ^13^C NMR (100 MHz, CDCl_3_) *δ* 172.1, 160.8, 140.0, 134.6, 130.1, 127.93, 127.87, 126.0, 122.7, 119.4, 114.1, 110.6, 70.7, 68.7, 42.7; HRMS (ESI‐QTOF) *m*/*z* calcd for C_15_H_15_ClN_3_O_2_^+^ [M + H]^+^ 304.0847, found 304.0845.

**(*S*)‐*N*‐(Furan‐2‐Ylmethyl)‐1‐Oxo‐1,2,3,4‐Tetrahydropyrrolo[1,2‐*a*]pyrazine‐3‐Carboxamide** (**A3**). Compound **A3** was synthesized using the synthetic procedure outlined above, yield: 32%. ^1^H NMR (400 MHz, CDCl_3_) *δ* 9.15 (s, 1H, NH), 7.35 (dd, *J* = 1.9, 0.8 Hz, 1H), 6.98 (brs, 1H, NH), 6.96–6.94 (m, 1H), 6.81 (ddd, *J* = 3.8, 2.4, 1.4 Hz, 1H), 6.31 (dd, *J* = 3.2, 1.9 Hz, 1H), 6.28 (dt, *J* = 3.7, 2.6 Hz, 1H), 6.23–6.21 (m, 1H), 4.80 (dd, *J* = 10.8, 8.1 Hz, 1H), 4.65 (dd, *J* = 10.8, 8.6 Hz, 1H), 4.55 (dd, *J* = 8.6, 8.1 Hz, 1H), 4.54 (dd, *J* = 15.5, 6.2 Hz, 1H), 4.37 (dd, *J* = 15.5, 5.4 Hz, 1H); ^13^C NMR (100 MHz, CDCl_3_) *δ* 171.8, 160.7, 151.0, 142.5, 122.6, 119.5, 113.9, 110.60, 110.56, 107.8, 70.6, 68.7, 36.3; HRMS (ESI‐QTOF) *m*/*z* calcd for C_13_H_14_N_3_O_3_^+^ [M + H]^+^ 260.1030, found 260.1018.

**(*S*)‐*N*‐((5‐Methylfuran‐2‐Yl)methyl)‐1‐Oxo‐1,2,3,4‐Tetrahydropyrrolo[1,2‐*a*]pyrazine‐3‐Carboxamide** (**A4**). Compound **A4** was synthesized using the synthetic procedure outlined above, yield: 31%. ^1^H NMR (400 MHz, CDCl_3_) *δ* 9.11 (brs, 1H, NH), 6.95 (td, *J* = 2.7, 1.4 Hz, 1H), 6.91 (brs, 1H, NH), 6.81 (ddd, *J* = 3.8, 2.4, 1.5 Hz, 1H), 6.28 (dt, *J* = 3.7, 2.6 Hz, 1H), 6.10 (d, *J* = 3.1 Hz, 1H), 5.89–5.87 (m, 1H), 4.80 (dd, *J* = 10.8, 8.0 Hz, 1H), 4.65 (dd, *J* = 10.8, 8.6 Hz, 1H), 4.55 (dd, *J* = 8.6, 8.1 Hz, 1H), 4.48 (dd, *J* = 15.5, 6.0 Hz, 1H), 4.30 (dd, *J* = 15.4, 5.2 Hz, 1H), 2.26 (d, *J* = 1.0 Hz, 3H); ^13^C NMR (100 MHz, CDCl_3_) *δ* 171.7, 160.7, 152.2, 149.0, 122.6, 119.5, 113.9, 110.5, 108.7, 106.4, 70.6, 68.7, 36.4, 13.7; HRMS (ESI‐QTOF) *m*/*z* calcd for C_14_H_16_N_3_O_3_^+^ [M + H]^+^ 274.1186, found 274.1181.

**(*S*)‐*N*‐(Thiophen‐2‐Ylmethyl)‐1‐Oxo‐1,2,3,4‐Tetrahydropyrrolo[1,2‐*a*]pyrazine‐3‐Carboxamide** (**A5**). Compound **A5** was synthesized using the synthetic procedure outlined above, yield: 55%. ^1^H NMR (400 MHz, CDCl_3_) *δ* 9.30 (brs, 1H, NH), 7.21 (dd, *J* = 4.5, 1.9 Hz, 1H), 7.07 (brs, 1H, NH), 6.95–6.92 (m, 3H), 6.81–6.79 (m, 1H), 6.27 (dt, *J* = 3.7, 2.5 Hz, 1H), 4.79 (dd, *J* = 10.8, 8.0 Hz, 1H), 4.69 (dd, *J* = 15.3, 6.2 Hz, 1H), 4.65 (dd, *J* = 10.8, 8.6 Hz, 1H), 4.56 (dd, *J* = 8.6, 8.1 Hz, 1H), 4.53 (dd, *J* = 15.3, 5.4 Hz, 1H); ^13^C NMR (100 MHz, CDCl_3_) *δ* 171.7, 160.8, 140.5, 127.1, 126.2, 125.5, 122.6, 119.4, 114.0, 110.6, 70.6, 68.6, 38.1; HRMS (ESI‐QTOF) *m*/*z* calcd for C_13_H_14_N_3_O_2_S^+^ [M + H]^+^ 276.0801, found 276.0805.

**(*S*)‐*N*‐2‐Dibenzyl‐1‐Oxo‐1,2,3,4‐Tetrahydropyrrolo[1,2‐*a*]pyrazine‐3‐Carboxamide** (**B1**). Compound **B1** was synthesized using the synthetic procedure outlined above, yield: 35%. ^1^H NMR (400 MHz, CDCl_3_) *δ* 7.32–7.25 (m, 3H), 7.12–7.07 (m, 5H), 6.93–6.90 (m, 2H), 6.89–6.86 (m, 2H), 6.26 (dd, *J* = 3.9, 2.7 Hz, 1H), 6.23 (brs, 1H, NH), 5.95 (d, *J* = 16.0 Hz, 1H), 5.33 (d, *J* = 16.0 Hz, 1H), 4.77 (dd, *J* = 10.9, 7.8 Hz, 1H), 4.53 (dd, *J* = 10.9, 8.7 Hz, 1H), 4.38 (dd, *J* = 8.6, 7.8 Hz, 1H), 4.30 (dd, *J* = 15.1, 6.9 Hz, 1H), 3.99 (dd, *J* = 15.1, 5.8 Hz, 1H); ^13^C NMR (100 MHz, CDCl_3_) *δ* 171.7, 159.9, 147.2, 146.5, 140.5, 128.7, 127.1, 126.6, 125.7, 125.3, 124.1, 119.8, 117.3, 109.7, 69.3, 69.2, 51.9, 38.0; HRMS (ESI‐QTOF) *m*/*z* calcd for C_22_H_22_N_3_O_2_^+^ [M + H]^+^ 360.1707, found 360.1710.

**(*S*)‐*N*‐(3‐Chlorobenzyl)‐2‐Benzyl‐1‐Oxo‐1,2,3,4‐Tetrahydropyrrolo[1,2‐*a*]pyrazine‐3‐Carboxamide** (**B2**). Compound **B2** was synthesized using the synthetic procedure outlined above, yield: 62%. ^1^H NMR (400 MHz, CDCl_3_) *δ* 7.23–7.21 (m, 2H), 7.16–7.08 (m, 3H), 7.04–7.03 (m, 1H), 6.97–6.91 (m, 3H), 6.89–6.85 (m, 2H), 6.27 (dd, *J* = 3.9, 2.6 Hz, 1H), 6.15 (t, *J* = 6.2 Hz, 1H, NH), 6.00 (d, *J* = 16.1 Hz, 1H), 5.31 (d, *J* = 16.1 Hz, 1H), 4.76 (dd, *J* = 10.9, 7.6 Hz, 1H), 4.52 (dd, *J* = 10.9, 8.7 Hz, 1H), 4.37 (dd, *J* = 8.7, 7.6 Hz, 1H), 4.25 (dd, *J* = 15.3, 7.1 Hz, 1H), 3.88 (dd, *J* = 15.3, 5.9 Hz, 1H); ^13^C NMR (100 MHz, CDCl_3_) *δ* 172.6, 159.9, 140.3, 139.4, 134.4, 129.9, 129.1, 128.8, 127.47, 127.45, 127.2, 125.47, 125.46, 119.6, 116.9, 108.9, 69.4, 69.2, 52.6, 42.2; HRMS (ESI‐QTOF) *m*/*z* calcd for C_22_H_21_ClN_3_O_2_^+^ [M + H]^+^ 394.1317, found 394.1319.

**(*S*)‐*N*‐(Furan‐2‐Ylmethyl)‐2‐Benzyl‐1‐Oxo‐1,2,3,4‐Tetrahydropyrrolo[1,2‐*a*]pyrazine‐3‐Carboxamide** (**B3**). Compound **B3** was synthesized using the synthetic procedure outlined above, yield: 29%. ^1^H NMR (400 MHz, CDCl_3_) *δ* 7.38–7.27 (m, 2H), 7.26–7.17 (m, 2H), 6.95–6.92 (m, 3H), 6.90 (dd, *J* = 3.9, 1.8 Hz, 1H), 6.30 (dd, *J* = 3.3, 1.8 Hz, 1H), 6.26 (dd, *J* = 3.9, 2.6 Hz, 1H), 6.21 (brs, 1H, NH), 6.08 (d, *J* = 3.3 Hz, 1H), 5.93 (d, *J* = 15.9 Hz, 1H), 5.37 (d, *J* = 15.9 Hz, 1H), 4.72 (dd, *J* = 11.0, 7.7 Hz, 1H), 4.50 (dd, *J* = 11.0, 8.6 Hz, 1H), 4.36 (t, *J* = 8.3 Hz, 1H), 4.28 (dd, *J* = 15.6, 6.8 Hz, 1H), 3.97 (dd, *J* = 15.6, 5.6 Hz, 1H); ^13^C NMR (100 MHz, CDCl_3_) *δ* 172.2, 159.9, 151.3, 142.0, 139.1, 128.92, 128.87, 127.3, 125.8, 119.7, 116.8, 110.5, 108.8, 107.2, 69.3, 69.2, 52.5, 36.0; HRMS (ESI‐QTOF) *m*/*z* calcd for C_20_H_20_N_3_O_3_^+^ [M + H]^+^ 350.1499, found 350.1488.

**(*S*)‐*N*‐((5‐Methylfuran‐2‐Yl)methyl)‐2‐Benzyl‐1‐Oxo‐1,2,3,4‐Tetrahydropyrrolo[1,2‐*a*]pyrazine‐3‐Carboxamide (B4)**. Compound **B4** was synthesized using the synthetic procedure outlined above, yield: 31%. ^1^H NMR (400 MHz, CDCl_3_) *δ* 7.28–7.19 (m, 3H), 6.95 (d, *J* = 6.7 Hz, 2H), 6.93–6.92 (m, 1H), 6.89 (dd, *J* = 3.9, 1.8 Hz, 1H), 6.25 (dd, *J* = 3.9, 2.6 Hz, 1H), 6.22 (brs, 1H, NH), 5.96 (d, *J* = 3.1 Hz, 1H), 5.91 (d, *J* = 15.9 Hz, 1H), 5.87–5.86 (m, 1H), 5.39 (d, *J* = 15.9 Hz, 1H), 4.73 (dd, *J* = 10.9, 7.9 Hz, 1H), 4.50 (dd, *J* = 10.9, 8.6 Hz, 1H), 4.36 (dd, *J* = 8.6, 7.9 Hz, 1H), 4.25 (dd, *J* = 15.5, 6.7 Hz, 1H), 3.93 (dd, *J* = 15.5, 5.4 Hz, 1H), 2.26 (s, 3H); ^13^C NMR (100 MHz, CDCl_3_) *δ* 172.1, 159.9, 151.7, 149.4, 139.1, 128.90, 128.85, 127.3, 125.9, 119.8, 116.8, 108.8, 108.1, 106.3, 69.30, 69.26, 52.5, 36.1, 13.7; HRMS (ESI‐QTOF) *m*/*z* calcd for C_21_H_22_N_3_O_3_^+^ [M + H]^+^ 364.1656, found 364.1657.

**(*S*)‐*N*‐(Thiophen‐2‐Ylmethyl)‐2‐Benzyl‐1‐Oxo‐1,2,3,4‐Tetrahydropyrrolo[1,2‐*a*]pyrazine‐3‐Carboxamide** (**B5**). Compound **B5** was synthesized using the synthetic procedure outlined above, yield: 55%. ^1^H NMR (400 MHz, CDCl_3_) *δ* 7.25–7.19 (m, 3H), 7.18 (dd, *J* = 5.1, 1.2 Hz, 1H), 6.94–6.89 (m, 5H), 6.82–6.81 (m, 1H), 6.26 (dd, *J* = 4.0, 2.6 Hz, 1H), 6.21 (brs, 1H, NH), 5.96 (d, *J* = 16.0 Hz, 1H), 5.33 (d, *J* = 16.0 Hz, 1H), 4.73 (dd, *J* = 10.9, 7.6 Hz, 1H), 4.50 (dd, *J* = 10.9, 8.6 Hz, 1H), 4.39 (dd, *J* = 15.4, 6.8 Hz, 1H), 4.36 (dd, *J* = 8.6, 7.6 Hz, 1H), 4.12 (dd, *J* = 15.4, 5.8 Hz, 1H); ^13^C NMR (100 MHz, CDCl_3_) *δ* 172.2, 159.9, 141.2, 139.3, 129.0, 128.9, 127.2, 126.8, 125.7, 125.6, 124.9, 119.7, 116.8, 108.8, 69.3, 69.2, 52.6, 37.8; HRMS (ESI‐QTOF) *m*/*z* calcd for C_20_H_20_N_3_O_2_S^+^ [M + H]^+^ 366.1271, found 366.1271.

**(*S*)‐*N*‐Benzyl‐2‐(4‐Cyanobenzyl)‐1‐Oxo‐1,2,3,4‐Tetrahydropyrrolo[1,2‐*a*]pyrazine‐3‐Carboxamide** (**C1**). Compound **C1** was synthesized using the synthetic procedure outlined above, yield: 78%. ^1^H NMR (400 MHz, CDCl_3_) *δ* 7.38–7.31 (m, 3H), 7.28 (d, *J* = 8.3 Hz, 2H), 7.15–7.12 (m, 2H), 6.93–6.91 (m, 3H), 6.89 (dd, *J* = 2.6, 1.8 Hz, 1H), 6.28 (dd, *J* = 3.8, 2.7 Hz, 1H), 6.18 (brs, 1H, NH), 5.96 (d, *J* = 16.6 Hz, 1H), 5.38 (d, *J* = 16.6 Hz, 1H), 4.77 (dd, *J* = 10.9, 8.1 Hz, 1H), 4.56 (dd, *J* = 10.9, 8.7 Hz, 1H), 4.43–4.37 (m, 2H), 4.07 (dd, *J* = 15.1, 5.5 Hz, 1H); ^13^C NMR (100 MHz, CDCl_3_) *δ* 172.0, 159.9, 144.5, 137.8, 132.6, 128.9, 128.7, 127.8, 127.4, 126.4, 119.8, 118.5, 117.2, 111.2, 109.6, 69.5, 69.3, 52.0, 42.9; HRMS (ESI‐QTOF) *m*/*z* calcd for C_23_H_21_N_4_O_2_^+^ [M + H]^+^ 385.1659, found 385.1659.

**(*S*)‐*N*‐(3‐Chlorobenzyl)‐2‐(4‐Cyanobenzyl)‐1‐Oxo‐1,2,3,4‐Tetrahydropyrrolo[1,2‐*a*]pyrazine‐3‐Carboxamide** (**C2**). Compound **C2** was synthesized using the synthetic procedure outlined above, yield: 48%. ^1^H NMR (400 MHz, CDCl_3_) *δ* 7.37 (d, *J* = 8.2 Hz, 2H), 7.30–7.27 (m, 2H), 7.08 (s, 1H), 7.05–7.01 (m, 1H), 6.97–6.93 (m, 3H), 6.90 (t, *J* = 2.2 Hz, 1H), 6.29 (dd, *J* = 4.0, 2.6 Hz, 1H), 6.17 (t, *J* = 6.3 Hz, 1H, NH), 5.96 (d, *J* = 16.7 Hz, 1H), 5.43 (d, *J* = 16.7 Hz, 1H), 4.77 (dd, *J* = 10.9, 8.0 Hz, 1H), 4.55 (dd, *J* = 10.9, 8.7 Hz, 1H), 4.43–4.36 (m, 2H), 4.00 (dd, *J* = 15.5, 5.5 Hz, 1H); ^13^C NMR (100 MHz, CDCl_3_) *δ* 172.1, 156.0, 144.7, 139.9, 134.7, 132.6, 130.2, 128.8, 127.9, 127.2, 126.4, 125.6, 119.7, 118.4, 117.4, 111.2, 109.6, 69.4, 69.2, 52.1, 42.2; HRMS (ESI‐QTOF) *m*/*z* calcd for C_23_H_20_ClN_4_O_2_^+^ [M + H]^+^ 419.1269, found 419.1268.

**(*S*)‐*N*‐(Furan‐2‐Ylmethyl)‐2‐(4‐Cyanobenzyl)‐1‐Oxo‐1,2,3,4‐Tetrahydropyrrolo[1,2‐*a*]pyrazine‐3‐Carboxamide (C3)**. Compound **C3** was synthesized using the synthetic procedure outlined above, yield: 43%. ^1^H NMR (400 MHz, CDCl_3_) *δ* 7.53 (d, *J* = 8.1 Hz, 2H), 7.33–7.32 (m, 1H), 7.05 (d, *J* = 8.1 Hz, 2H), 6.93–6.90 (m, 2H), 6.31–6.27 (m, 3H), 6.10 (d, *J* = 3.3 Hz, 1H), 5.99 (d, *J* = 16.2 Hz, 1H), 5.45 (d, *J* = 16.2 Hz, 1H), 4.74 (dd, *J* = 11.0, 8.1 Hz, 1H), 4.53 (dd, *J* = 11.0, 8.6 Hz, 1H), 4.39–4.31 (m, 2H), 4.00 (dd, *J* = 15.6, 5.3 Hz, 1H); ^13^C NMR (100 MHz, CDCl_3_) *δ* 171.7, 159.8, 150.8, 144.5, 142.4, 132.7, 128.7, 126.6, 119.8, 118.6, 117.2, 111.3, 110.7, 109.5, 107.7, 69.28, 69.25, 52.0, 36.0; HRMS (ESI‐QTOF) *m*/*z* calcd for C_21_H_19_N_4_O_3_^+^ [M + H]^+^ 375.1452, found 375.1449.

**(*S*)‐*N*‐((5‐Methylfuran‐2‐Yl)methyl)‐2‐(4‐Cyanobenzyl)‐1‐Oxo‐1,2,3,4‐Tetrahydropyrrolo[1,2‐*a*]pyrazine‐3‐Carboxamide** (**C4**). Compound **C4** was synthesized using the synthetic procedure outlined above, yield: 56%. ^1^H NMR (400 MHz, CDCl_3_) *δ* 7.51 (d, *J* = 8.0 Hz, 2H), 7.03 (d, *J* = 7.9 Hz, 2H), 6.91 (d, *J* = 3.6 Hz, 2H), 6.28 (t, *J* = 3.7 Hz, 1H), 6.17 (t, *J* = 5.9 Hz, 1H, NH), 6.01 (d, *J* = 3.1 Hz, 1H), 5.95–5.91 (m, 2H), 5.46 (d, *J* = 16.5 Hz, 1H), 4.72 (dd, *J* = 11.0, 8.2 Hz, 1H), 4.52 (dd, *J* = 10.9, 8.7 Hz, 1H), 4.37 (t, *J* = 8.8 Hz, 1H), 4.30 (dd, *J* = 15.6, 6.4 Hz, 1H), 4.04 (dd, *J* = 15.5, 5.3 Hz, 1H), 2.28 (s, 3H); ^13^C NMR (100 MHz, CDCl_3_) *δ* 171.6, 159.8, 152.1, 149.0, 144.5, 132.7 (2C), 128.6, 126.6 (2C), 119.8, 118.6, 117.2, 111.4, 109.5, 108.4, 106.5, 69.3 (2C), 52.0, 36.2, 13.7; HRMS (ESI‐QTOF) *m*/*z* calcd for C_22_H_21_N_4_O_3_^+^ [M + H]^+^ 389.1608, found 389.1608.

**(*S*)‐*N*‐(Thiophen‐2‐Ylmethyl)‐2‐(4‐Cyanobenzyl)‐1‐Oxo‐1,2,3,4‐Tetrahydropyrrolo[1,2‐*a*]pyrazine‐3‐Carboxamide** (**C5**). Compound **C5** was synthesized using the synthetic procedure outlined above, yield: 62%. ^1^H NMR (400 MHz, CDCl_3_) *δ* 7.39 (d, *J* = 8.3 Hz, 2H), 7.24 (dd, *J* = 5.1, 1.3 Hz, 1H), 7.00–6.96 (m, 3H), 6.92–6.88 (m, 3H), 6.28 (dd, *J* = 4.0, 2.6 Hz, 1H), 6.21 (t, *J* = 5.9 Hz, 1H, NH), 5.97 (d, *J* = 16.6 Hz, 1H), 5.40 (d, *J* = 16.6 Hz, 1H), 4.74 (dd, *J* = 10.9, 8.1 Hz, 1H), 4.54 (dd, *J* = 10.9, 8.7 Hz, 1H), 4.49 (dd, *J* = 15.5, 6.5 Hz, 1H), 4.38 (t, *J* = 8.4 Hz, 1H), 4.29 (dd, *J* = 15.4, 5.6 Hz, 1H); ^13^C NMR (100 MHz, CDCl_3_) *δ* 171.7, 159.9, 144.6, 140.7, 132.6, 128.7, 127.2, 126.5, 125.9, 125.4, 119.7, 118.6, 117.2, 111.2, 109.5, 69.3, 69.2, 52.0, 38.0; HRMS (ESI‐QTOF) *m*/*z* calcd for C_21_H_19_N_4_O_2_S^+^ [M + H]^+^ 391.1223, found 391.1225.

**(*S*)‐*N*‐Benzyl‐1‐Oxo‐2‐(4‐(Trifluoromethyl)benzyl)‐1,2,3,4‐Tetrahydropyrrolo[1,2‐*a*]pyrazine‐3‐Carboxamide** (**D1**). Compound **D1** was synthesized using the synthetic procedure outlined above, yield: 50%. ^1^H NMR (400 MHz, CDCl_3_) *δ* 7.38 (d, *J* = 8.1 Hz, 2H), 7.34–7.26 (m, 3H), 7.14–7.11 (m, 2H), 6.98 (d, *J* = 8.0 Hz, 2H), 6.92–6.89 (m, 2H), 6.34 (brs, 1H, NH), 6.28 (dd, *J* = 3.9, 2.7 Hz, 1H), 5.97 (d, *J* = 16.3 Hz, 1H), 5.42 (d, *J* = 16.3 Hz, 1H), 4.78 (dd, *J* = 10.9, 8.1 Hz, 1H), 4.55 (dd, *J* = 10.9, 8.7 Hz, 1H), 4.44–4.38 (m, 2H), 3.99 (dd, *J* = 15.2, 5.4 Hz, 1H); ^13^C NMR (100 MHz, CDCl_3_) *δ* 172.1, 159.9, 143.2, 138.0, 129.6 (*J*_CF_ = 32.5 Hz), 128.8, 128.7, 127.5, 127.3, 126.2, 125.8 (*J*_CF_ = 3.6 Hz), 124.0 (*J*_CF_ = 273 Hz), 119.8, 117.1, 109.3, 69.4, 69.3, 51.9, 42.7; HRMS (ESI‐QTOF) *m*/*z* calcd for C_23_H_21_F_3_N_3_O_2_^+^ [M + H]^+^ 428.1580, found 428.1586.

**(*S*)‐*N*‐(3‐Chlorobenzyl)‐1‐Oxo‐2‐(4‐(Trifluoromethyl)benzyl)‐1,2,3,4‐Tetrahydropyrrolo[1,2‐*a*]pyrazine‐3‐Carboxamide** (**D2**). Compound **D2** was synthesized using the synthetic procedure outlined above, yield: 63%. ^1^H NMR (400 MHz, CDCl_3_) *δ* 7.42 (d, *J* = 8.0 Hz, 2H), 7.25–7.21 (m, 2H), 7.08–7.07 (m, 1H), 7.02–6.96 (m, 3H), 6.95–6.90 (m, 2H), 6.29 (dd, *J* = 3.9, 2.7 Hz, 1H), 6.28 (brs, 1H, NH), 6.01 (d, *J* = 16.3 Hz, 1H), 5.43 (d, *J* = 16.4 Hz, 1H), 4.78 (dd, *J* = 10.9, 8.0 Hz, 1H), 4.55 (dd, *J* = 10.9, 8.7 Hz, 1H), 4.39 (dd, *J* = 8.7, 8.0 Hz, 1H), 4.38 (dd, *J* = 15.5, 7.4 Hz, 1H), 3.88 (dd, *J* = 15.5, 5.5 Hz, 1H); ^13^C NMR (100 MHz, CDCl_3_) *δ* 172.2, 160.0, 143.4 (*J*_CF_ = 1.2 Hz), 140.1, 134.6, 130.0, 129.6 (*J*_CF_ = 32.5 Hz), 128.9, 127.7, 127.2, 126.1, 125.8 (*J*_CF_ = 3.8 Hz), 125.4, 124.0 (*J*_CF_ = 272 Hz), 119.7, 117.2, 109.4, 69.4, 69.2, 52.0, 42.1; HRMS (ESI‐QTOF) *m*/*z* calcd for C_23_H_20_ClF_3_N_3_O_2_^+^ [M + H]^+^ 462.1191, found 462.1192.

**(*S*)‐*N*‐(Furan‐2‐Ylmethyl)‐1‐Oxo‐2‐(4‐(Trifluoromethyl)benzyl)‐1,2,3,4‐Tetrahydropyrrolo[1,2‐*a*]pyrazine‐3‐Carboxamide** (**D3**). Compound **D3** was synthesized using the synthetic procedure outlined above, yield: 50%. ^1^H NMR (400 MHz, CDCl_3_) *δ* 7.52 (d, *J* = 8.2 Hz, 2H), 7.33–7.32 (m, 1H), 7.05 (d, *J* = 8.0 Hz, 2H), 6.92–6.90 (m, 2H), 6.33–6.27 (m, 3H), 6.10 (d, *J* = 3.2 Hz, 1H), 5.98 (d, *J* = 16.3 Hz, 1H), 5.45 (d, *J* = 16.3 Hz, 1H), 4.73 (dd, *J* = 11.0, 8.1 Hz, 1H), 4.52 (dd, *J* = 10.9, 8.7 Hz, 1H), 4.29–4.31 (m, 2H), 4.00 (dd, *J* = 15.6, 5.4 Hz, 1H); ^13^C NMR (100 MHz, CDCl_3_) *δ* 171.9, 159.9, 151.1, 143.2, 142.3, 129.7 (*J*_CF_ = 32.4 Hz), 128.7, 126.3, 125.9 (*J*_CF_ = 3.6 Hz), 124.1 (*J*_CF_ = 272 Hz), 119.8, 117.1, 110.5, 109.4, 107.4, 69.2 (2C), 52.0, 35.9; HRMS (ESI‐QTOF) *m*/*z* calcd for C_21_H_19_F_3_N_3_O_3_^+^ [M + H]^+^ 418.1373, found 418.1368.

**(*S*)‐*N*‐((5‐Methylfuran‐2‐Yl)methyl)‐1‐2‐(4‐(Trifluoromethyl)benzyl)‐Oxo‐1,2,3,4‐Tetrahydropyrrolo[1,2‐*a*]pyrazine‐3‐Carboxamide** (**D4**). Compound **D4** was synthesized using the synthetic procedure outlined above, yield: 29%. ^1^H NMR (400 MHz, CDCl_3_) *δ* 7.53 (d, *J* = 8.0 Hz, 2H), 7.06 (d, *J* = 7.8 Hz, 2H), 6.92–6.90 (m, 2H), 6.30–6.26 (m, 2H), 5.99–5.93 (m, 2H), 5.88–5.86 (m, 1H), 5.47 (d, *J* = 16.2 Hz, 1H), 4.73 (dd, *J* = 10.9, 8.1 Hz, 1H), 4.52 (dd, *J* = 11.0, 8.6 Hz, 1H), 4.38 (t, *J* = 8.4 Hz, 1H), 4.30 (dd, *J* = 15.5, 6.8 Hz, 1H), 3.94 (dd, *J* = 15.6, 5.3 Hz, 1H), 2.25 (s, 3H); ^13^C NMR (100 MHz, CDCl_3_) *δ* 171.7, 159.9, 152.0, 149.2, 143.2, 129.7 (*J*_CF_ = 32.8 Hz), 128.7, 126.4, 125.9 (*J*_CF_ = 3.7 Hz), 124.1 (*J*_CF_ = 272 Hz), 119.9, 117.1, 109.4, 108.2, 106.3, 69.30, 69.26, 52.0, 36.0, 13.6; HRMS (ESI‐QTOF) *m*/*z* calcd for C_22_H_21_F_3_N_3_O_3_^+^ [M + H]^+^ 432.1530, found 432.1524.

**(*S*)‐*N*‐(Thiophen‐2‐Ylmethyl)‐1‐Oxo‐2‐(4‐(Trifluoromethyl)benzyl)‐1,2,3,4‐Tetrahydropyrrolo[1,2‐*a*]pyrazine‐3‐Carboxamide** (**D5**). Compound **D5** was synthesized using the synthetic procedure outlined above, yield: 33%. ^1^H NMR (400 MHz, CDCl_3_) *δ* 7.47 (d, *J* = 8.3 Hz, 2H), 7.21–7.19 (m, 1H), 7.02 (d, *J* = 7.8 Hz, 2H), 6.94–6.91 (m, 3H), 6.84 (d, *J* = 3.5 Hz, 1H), 6.33 (brs, 1H, NH), 6.28 (t, *J* = 3.5 Hz, 1H), 5.99 (d, *J* = 16.3 Hz, 1H), 5.42 (d, *J* = 16.3 Hz, 1H), 4.75 (dd, *J* = 10.9, 8.0 Hz, 1H), 4.54 (dd, *J* = 11.0, 8.5 Hz, 1H), 4.49 (dd, *J* = 15.4, 6.7 Hz, 1H), 4.38 (t, *J* = 8.4 Hz, 1H), 4.17 (dd, *J* = 15.4, 5.5 Hz, 1H); ^13^C NMR (100 MHz, CDCl_3_) *δ* 171.8, 159.9, 143.3, 140.9, 129.6 (*J*_CF_ = 32.6 Hz), 128.7, 127.0, 126.3, 125.9 (*J*_CF_ = 3.7 Hz), 125.7, 125.2, 124.1 (*J*_CF_ = 272 Hz), 119.8, 117.1, 109.4, 69.3, 69.2, 52.0, 37.8; HRMS (ESI‐QTOF) *m*/*z* calcd for C_21_H_19_F_3_N_3_O_2_S^+^ [M + H]^+^ 434.1145, found 434.1145.

**(*S*)‐*N*‐Benzyl‐1‐Oxo‐2‐(4‐(Trifluoromethoxy)Benzyl)‐1,2,3,4‐Tetrahydropyrrolo[1,2‐*a*]pyrazine‐3‐Carboxamide** (**E1**). Compound **E1** was synthesized using the synthetic procedure outlined above, yield: 75%. ^1^H NMR (400 MHz, CDCl_3_) *δ* 7.34–7.27 (m, 3H), 7.16–7.13 (m, 2H), 6.97–6.88 (m, 6H), 6.40 (t, *J* = 6.3 Hz, 1H, NH), 6.26 (dd, *J* = 3.9, 2.7 Hz, 1H), 5.89 (d, *J* = 15.9 Hz, 1H), 5.38 (d, *J* = 15.9 Hz, 1H), 4.80 (dd, *J* = 10.9, 8.1 Hz, 1H), 4.56 (dd, *J* = 10.9, 8.7 Hz, 1H), 4.45 – 4.39 (m, 2H), 4.06 (dd, *J* = 15.1, 5.5 Hz, 1H); ^13^C NMR (100 MHz, CDCl_3_) *δ* 172.1, 160.0, 148.3 (*J*_CF_ = 1.3 Hz), 138.1, 137.7, 128.8, 128.6, 127.6, 127.44, 127.38, 121.3, 120.5 (*J*_CF_ = 257 Hz), 119.7, 117.0, 109.3, 69.37, 69.36, 51.6, 42.9; HRMS (ESI‐QTOF) *m*/*z* calcd for C_23_H_21_F_3_N_3_O_3_^+^ [M + H]^+^ 444.1530, found 444.1542.

**(*S*)‐*N*‐(3‐Chlorobenzyl)‐1‐Oxo‐2‐(4‐(Trifluoromethoxy)Benzyl)‐1,2,3,4‐Tetrahydropyrrolo[1,2‐*a*]pyrazine‐3‐Carboxamide** (**E2**). Compound **E2** was synthesized using the synthetic procedure outlined above, yield: 68%. ^1^H NMR (400 MHz, CDCl_3_) *δ* 7.25–7.24 (m, 2H), 7.11 (s, 1H), 7.02–6.98 (m, 3H), 6.93–6.90 (m, 4H), 6.32 (t, *J* = 6.2 Hz, 1H, NH), 6.27 (dd, *J* = 3.9, 2.6 Hz, 1H), 5.92 (d, *J* = 16.1 Hz, 1H), 5.38 (d, *J* = 16.1 Hz, 1H), 4.79 (dd, *J* = 11.0, 7.9 Hz, 1H), 4.55 (dd, *J* = 10.9, 8.7 Hz, 1H), 4.40 (t, *J* = 8.3 Hz, 1H), 4.39 (dd, *J* = 15.4, 7.1 Hz, 1H), 3.95 (dd, *J* = 15.5, 5.5 Hz, 1H); ^13^C NMR (100 MHz, CDCl_3_) *δ* 172.3, 160.0, 148.3 (*J* = 1.1 Hz), 140.1, 137.9, 134.6, 130.0, 128.8, 127.7, 127.4, 127.3, 125.5, 121.3, 120.5 (*J*_CF_ = 258 Hz), 119.6, 117.1, 109.3, 69.4, 69.2, 51.7, 42.2; HRMS (ESI‐QTOF) *m*/*z* calcd for C_23_H_20_ClF_3_N_3_O_3_^+^ [M + H]^+^ 478.1140, found 478.1154.

**(*S*)‐*N*‐(Furan‐2‐Ylmethyl)‐1‐Oxo‐2‐(4‐(Trifluoromethoxy)Benzyl)‐1,2,3,4‐Tetrahydropyrrolo[1,2‐*a*]pyrazine‐3‐Carboxamide** (**E3**). Compound **E3** was synthesized using the synthetic procedure outlined above, yield: 52%. ^1^H NMR (400 MHz, CDCl_3_) *δ* 7.34–7.33 (m, 1H), 7.11 (d, *J* = 8.3 Hz, 2H), 6.99 (d, *J* = 8.6 Hz, 2H), 6.91–6.89 (m, 2H), 6.38 (brs, 1H, NH), 6.31 –6.30 (m, 1H), 6.26 (dd, *J* = 3.9, 2.6 Hz, 1H), 6.13 (d, *J* = 3.2 Hz, 1H), 5.90 (d, *J* = 15.9 Hz, 1H), 5.41 (d, *J* = 16.0 Hz, 1H), 4.75 (dd, *J* = 11.0, 8.1 Hz, 1H), 4.53 (dd, *J* = 10.9, 8.7 Hz, 1H), 4.40–4.34 (m, 2H), 4.07 (dd, *J* = 15.6, 5.4 Hz, 1H); ^13^C NMR (100 MHz, CDCl_3_) *δ* 171.9, 160.0, 151.1, 148.4 (*J*_CF_ = 1.8 Hz), 142.3, 137.7, 128.6, 127.5, 121.4, 120.5 (*J*_CF_ = 257 Hz), 119.8, 117.0, 110.5, 109.3, 107.4, 69.3, 69.2, 51.6, 35.9; HRMS (ESI‐QTOF) *m*/*z* calcd for C_21_H_19_F_3_N_3_O_4_^+^ [M + H]^+^ 434.1322, found 434.1318.

**(*S*)‐*N*‐((5‐Methylfuran‐2‐Yl)methyl)‐1‐Oxo‐2‐(4‐(Trifluoromethoxy)Benzyl)‐1,2,3,4‐Tetrahydropyrrolo[1,2‐*a*]pyrazine‐3‐Carboxamide** (**E4**). Compound **E4** was synthesized using the synthetic procedure outlined above, yield: 60%. ^1^H NMR (400 MHz, CDCl_3_) *δ* 7.11 (d, *J* = 8.2 Hz, 2H), 7.00 (d, *J* = 8.6 Hz, 2H), 6.91–6.88 (m, 2H), 6.37 (brs, 1H, NH), 6.26 (dd, *J* = 3.9, 2.7 Hz, 1H), 6.00 (d, *J* = 3.1 Hz, 1H), 5.88 (d, *J* = 15.8 Hz, 1H), 5.88 (d, *J* = 2.0 Hz, 1H), 5.43 (d, *J* = 15.9 Hz, 1H), 4.75 (dd, *J* = 10.9, 8.1 Hz, 1H), 4.53 (dd, *J* = 11.0, 8.7 Hz, 1H), 4.39 (t, *J* = 8.4 Hz, 1H), 4.33 (dd, *J* = 15.5, 6.6 Hz, 1H), 4.01 (dd, *J* = 15.5, 5.3 Hz, 1H), 2.26 (s, 3H); ^13^C NMR (100 MHz, CDCl_3_) *δ* 171.9, 160.0, 152.0, 149.2, 148.4 (*J*_CF_ = 1.9 Hz), 137.6, 128.6, 127.6, 121.4 (*J*_CF_ = 1.0 Hz), 120.5 (*J*_CF_ = 257 Hz), 119.7, 117.0, 109.2, 108.2, 106.4, 69.3, 69.2, 51.6, 36.1, 13.6; HRMS (ESI‐QTOF) *m*/*z* calcd for C_22_H_21_F_3_N_3_O_4_^+^ [M + H]^+^ 448.1479, found 448.1482.

**(*S*)‐*N*‐(Thiophen‐2‐Ylmethyl)‐1‐Oxo‐2‐(4‐(Trifluoromethoxy)Benzyl)‐1,2,3,4‐Tetrahydropyrrolo[1,2‐*a*]pyrazine‐3‐Carboxamide** (**E5**). Compound **E5** was synthesized using the synthetic procedure outlined above, yield: 86%. ^1^H NMR (400 MHz, CDCl_3_) *δ* 7.21 (dd, J = 5.1, 1.3 Hz, 1H), 7.05 (d, J = 7.8 Hz, 2H), 6.98–6.92 (m, 3H), 6.90 (d, J = 3.2 Hz, 2H), 6.86 (dd, J = 3.4, 1.1 Hz, 1H), 6.39 (brs, 1H, NH), 6.26 (t, J = 3.4 Hz, 1H), 5.90 (d, J = 16.0 Hz, 1H), 5.39 (d, J = 16.0 Hz, 1H), 4.76 (dd, J = 11.0, 8.0 Hz, 1H), 4.58–4.48 (m, 2H), 4.39 (dd, J = 8.7, 8.0 Hz, 1H), 4.24 (dd, J = 15.3, 5.7 Hz, 1H); ^13^C NMR (100 MHz, CDCl_3_) *δ* 171.9, 160.0, 148.3 (J = 1.8 Hz), 140.9, 137.8, 128.7, 127.4, 126.9, 125.8, 125.2, 121.3 (J_CF_ = 1.0 Hz), 120.5 (J_CF_ = 257 Hz), 119.7, 117.0, 109.2, 69.27, 69.26, 51.6, 37.8; HRMS (ESI‐QTOF) *m*/*z* calcd for C_21_H_18_F_3_N_3_O_3_S [M + H]^+^: 450.1099, found 450.1097.

(***S*)‐*N*‐Benzyl‐2‐(4‐Nitrobenzyl)‐1‐Oxo‐1,2,3,4‐Tetrahydropyrrolo[1,2‐*a*]pyrazine‐3‐Carboxamide** (**F1**). Compound **F1** was synthesized using the synthetic procedure outlined above, yield: 66%. ^1^H NMR (400 MHz, CDCl_3_) *δ* 7.92 (d, *J* = 8.7 Hz, 2H), 7.30–7.26 (m, 3H), 7.06–7.03 (m, 2H), 6.98 (d, *J* = 8.7 Hz, 2H), 6.93 (dd, *J* = 4.0, 1.8 Hz, 1H), 6.90 (dd, *J* = 2.7, 1.7 Hz, 1H), 6.30 (dd, *J* = 3.9, 2.6 Hz, 1H), 6.22 (brs, 1H, NH), 6.01 (d, *J* = 16.7 Hz, 1H), 5.44 (d, *J* = 16.6 Hz, 1H), 4.78 (dd, *J* = 10.9, 8.2 Hz, 1H), 4.57 (dd, *J* = 11.0, 8.7 Hz, 1H), 4.41 (t, *J* = 8.5 Hz, 1H), 4.35 (dd, *J* = 15.1, 6.6 Hz, 1H), 4.09 (dd, *J* = 15.1, 5.6 Hz, 1H); ^13^C NMR (100 MHz, CDCl_3_) *δ* 171.9, 159.9, 147.1, 146.5, 137.6, 128.8, 128.7, 127.7, 127.1, 126.5, 124.0, 119.8, 117.3, 109.7, 69.4, 69.3, 51.8, 42.8; HRMS (ESI‐QTOF) *m*/*z* calcd for C_22_H_21_N_4_O_4_^+^ [M + H]^+^ 405.1557, found 405.1559.

**(*S*)‐*N*‐(3‐Chlorobenzyl)‐2‐(4‐Nitrobenzyl)‐1‐Oxo‐1,2,3,4‐Tetrahydropyrrolo[1,2‐*a*]pyrazine‐3‐Carboxamide** (**F2**). Compound **F2** was synthesized using the synthetic procedure outlined above, yield: 60%. ^1^H NMR (400 MHz, CDCl_3_) *δ* 7.96 (d, *J* = 8.7 Hz, 2H), 7.25–7.19 (m, 2H), 7.02–7.00 (m, 3H), 6.95 (dd, *J* = 3.9, 1.7 Hz, 1H), 6.93–6.91 (m, 2H), 6.31 (dd, *J* = 3.9, 2.6 Hz, 1H), 6.21 (t, *J* = 6.3 Hz, 1H, NH), 6.01 (d, *J* = 16.7 Hz, 1H), 5.48 (d, *J* = 16.7 Hz, 1H), 4.77 (dd, *J* = 10.9, 8.0 Hz, 1H), 4.56 (dd, *J* = 11.0, 8.7 Hz, 1H), 4.40 (t, *J* = 8.4 Hz, 1H), 4.35 (dd, *J* = 15.5, 7.0 Hz, 1H), 4.01 (dd, *J* = 15.5, 5.6 Hz, 1H); ^13^C NMR (100 MHz, CDCl_3_) *δ* 172.1, 160.0, 147.1, 146.6, 139.8, 134.7, 130.1, 128.8, 127.9, 127.0, 126.5, 125.3, 124.0, 119.7, 117.4, 109.7, 69.4, 69.2, 51.9, 42.2; HRMS (ESI‐QTOF) *m*/*z* calcd for C_22_H_20_ClN_4_O_4_^+^ [M + H]^+^ 439.1168, found 439.1167.

**(*S*)‐*N*‐(Furan‐2‐Ylmethyl)‐2‐(4‐Nitrobenzyl)‐1‐Oxo‐1,2,3,4‐Tetrahydropyrrolo[1,2‐*a*]pyrazine‐3‐Carboxamide** (**F3**). Compound **F3** was synthesized using the synthetic procedure outlined above, yield: 64%. ^1^H NMR (400 MHz, CDCl_3_) *δ* 8.10 (d, *J* = 8.7 Hz, 2H), 7.30–7.29 (m, 1H), 7.06 (d, *J* = 8.4 Hz, 2H), 6.94–6.91 (m, 2H), 6.30 (dd, *J* = 3.8, 2.6 Hz, 1H), 6.27 (dd, *J* = 3.2, 1.9 Hz, 1H), 6.17 (t, *J* = 6.1 Hz, 1H, NH), 6.06 (d, *J* = 3.3 Hz, 1H), 6.01 (d, *J* = 16.7 Hz, 1H), 5.48 (d, *J* = 16.7 Hz, 1H), 4.72 (dd, *J* = 11.0, 8.2 Hz, 1H), 4.52 (dd, *J* = 11.1, 8.6 Hz, 1H), 4.36 (t, *J* = 8.5 Hz, 1H), 4.27 (dd, *J* = 15.6, 6.3 Hz, 1H), 4.10 (dd, *J* = 15.5, 5.7 Hz, 1H); ^13^C NMR (100 MHz, CDCl_3_) *δ* 171.7, 159.8, 150.7, 147.3, 146.5, 142.4, 128.7, 126.6, 124.1, 119.8, 117.3, 110.5, 109.6, 107.5, 69.3, 69.2, 51.9, 35.9; HRMS (ESI‐QTOF) *m*/*z* calcd for C_20_H_19_N_4_O_5_^+^ [M + H]^+^ 395.1350, found 395.1351.

**(*S*)‐*N*‐((5‐Methylfuran‐2‐Yl)methyl)‐2‐(4‐Nitrobenzyl)‐1‐Oxo‐1,2,3,4‐Tetrahydropyrrolo[1,2‐*a*]pyrazine‐3‐Carboxamide (F4**). Compound **F4** was synthesized using the synthetic procedure outlined above, yield: 68%. ^1^H NMR (400 MHz, CDCl_3_) *δ* 8.10 (d, *J* = 8.7 Hz, 2H), 7.08 (d, *J* = 8.4 Hz, 2H), 6.93–6.91 (m, 2H), 6.30 (t, *J* = 3.3 Hz, 1H), 6.16 (t, *J* = 6.1 Hz, 1H, NH), 5.99 (d, *J* = 16.6 Hz, 1H), 5.92 (d, *J* = 3.2 Hz, 1H), 5.85–5.83 (m, 1H), 5.50 (d, *J* = 16.6 Hz, 1H), 4.71 (dd, *J* = 11.0, 8.1 Hz, 1H), 4.52 (dd, *J* = 10.9, 8.6 Hz, 1H), 4.37 (t, *J* = 8.4 Hz, 1H), 4.24 (dd, *J* = 15.5, 6.3 Hz, 1H), 4.02 (dd, *J* = 15.5, 5.5 Hz, 1H), 2.24 (s, 3H); ^13^C NMR (100 MHz, CDCl_3_) *δ* 171.6, 159.8, 152.1, 148.9, 147.3, 146.5, 128.7, 126.6, 124.1, 119.8, 117.2, 109.6, 108.3, 106.4, 69.30, 69.25, 51.9, 36.1, 13.6; HRMS (ESI‐QTOF) *m*/*z* calcd for C_21_H_21_N_4_O_5_^+^ [M + H]^+^ 409.1506, found 409.1511.

**(*S*)‐*N*‐(Thiophen‐2‐Ylmethyl)‐2‐(4‐Nitrobenzyl)‐1‐Oxo‐1,2,3,4‐Tetrahydropyrrolo[1,2‐*a*]pyrazine‐3‐Carboxamide** (**F5**). Compound **F5** was synthesized using the synthetic procedure outlined above, yield: 64%. ^1^H NMR (400 MHz, CDCl_3_) *δ* 8.01 (d, *J* = 8.7 Hz, 2H), 7.19 (d, *J* = 5.0 Hz, 1H), 7.02 (d, *J* = 8.5 Hz, 2H), 6.94–6.90 (m, 3H), 6.77 (d, *J* = 3.4 Hz, 1H), 6.30 (dd, *J* = 3.8, 2.7 Hz, 1H), 6.25 (t, *J* = 5.9 Hz, 1H, NH), 6.02 (d, *J* = 16.7 Hz, 1H), 5.45 (d, *J* = 16.7 Hz, 1H), 4.74 (dd, *J* = 10.9, 8.2 Hz, 1H), 4.55 (dd, *J* = 10.9, 8.7 Hz, 1H), 4.45 (dd, *J* = 15.4, 6.3 Hz, 1H), 4.39 (t, *J* = 8.4 Hz, 1H), 4.29 (dd, *J* = 15.4, 5.7 Hz, 1H); ^13^C NMR (100 MHz, CDCl_3_) *δ* 171.7, 159.9, 147.2, 146.5, 140.5, 128.7, 127.1, 126.6, 125.7, 125.3, 124.1, 119.8, 117.3, 109.7, 69.3, 69.2, 51.9, 38.0; HRMS (ESI‐QTOF) *m*/*z* calcd for C_20_H_19_N_4_O_4_S^+^ [M + H]^+^ 411.1122, found 411.1118.

### 4.6 Cell Culture

The human breast cancer cell lines, MCF‐7 and MDA‐MB‐468, were purchased from the American Type Culture Collection (ATCC, Manassas, VA, USA). Cells were cultured in RPMI1640 (Capricorn Scientific, Ebsdorfergrund, Germany) supplemented with 10% fetal bovine serum (FBS, Hyclone, Logan, UT, USA), 100 U/mL penicillin, and 100 μg/mL streptomycin (GenDEPOT, Barker, TX, USA) at 37 °C in a humidified incubator with 5% CO_2_.

### 4.7 Cell Viability Assays

Cells were seeded into 96‐well plates at a density of 7 × 10^3^ cells per well for MCF‐7 and 9 × 10^3^ cells per well for MDA‐MB‐468, then incubated for 24 h at 37 °C in a CO_2_ incubator. Following incubation, cells were treated with various concentrations of each compound, including gefitinib (MedKoo Biosciences, Chapel Hill, NC, USA), and further incubated for 48 h. Cell viability was determined by adding 3‐(4,5‐dimethylthiazol‐2‐yl)−2,5‐diphenyltetrazolium bromide (MTT, USB, Cleveland, Ohio, USA) solution to each well, followed by incubation at 37 °C for 1 h. After cell dissolution in dimethyl sulfoxide (DMSO), absorbance per well was measured at 540 nm using the MULTISKAN GO reader (Thermo Scientific, Waltham, MA, USA). The results are expressed as a percentage (%) of control cell viability.

### 4.8 Protein Extraction and Western Blotting Analysis

Cells were seeded in a 6‐well plate and treated with 10 µM of each compound. After treatment, the cells were lysed with ice‐cold RIPA buffer (Thermo Scientific, Waltham, MA, USA), and the protein concentration was quantified using BCA reagents (Thermo Scientific, Sunnyvale, CA, USA). Equal amounts of protein samples were separated by SDS‐PAGE using a 10% polyacrylamide gel and then transferred onto a polyvinylidene difluoride (PVDF) membrane (Millipore, Billerica, MA, USA). The membranes were subsequently incubated with primary antibodies (diluted at 1:5000): anti‐phospho‐ERK, anti‐ERK, anti‐phospho‐Akt (Ser473), anti‐phospho‐Akt (Thr308), anti‐Akt (Cell Signaling, Danvers, MA, USA); and anti‐GAPDH (Santa Cruz Biotechnology, Dallas, TX, USA). Antigen‐antibody complexes were detected using a Western Bright ECL HRP substrate kit (Advansta, Menlo Park, CA, USA).

### 4.9 Flow Cytometric Analysis With Annexin V and Propidium Iodide

Apoptotic and live cells were determined by fluorescence‐activated cell sorting (FACS) analysis following the instructions of the annexin V‐FITC Apoptosis Detection Kit (BD Biosciences, Bedford, MA, USA). After treating with gefitinib and compounds **B1** and **B3** for 24 h, cells were harvested, trypsinized, washed with cold PBS, and resuspended in 1X binding buffer. The stained cells with annexin V‐FITC and propidium iodide (PI) were analyzed using a Becton Dickinson FACSscan flow cytometer (BD Biosciences, San Jose, CA, USA) and BD FACSDiva software (BD Biosciences, San Jose, CA, USA).

## Author Contributions

**Narges Hosseininasab**: writing – review and editing, methodology, formal analysis, validation, investigation, data curation, conceptualization. **Sou Hyun Kim**: methodology, formal analysis, data curation, conceptualization. **Ye‐Mi Kwon**: methodology, formal analysis, writing, validation, investigation, data curation, conceptualization. **Jungjin Park**, **Chanhyun Jung**, **Kwanghee Lee**, **Beom‐Jun Wi**, and **Su‐Jin Yoo**: data curation, conceptualization. **Soonsil Hyun**: validation. **Young‐Suk Jung**: writing – review and editing, visualization, validation, investigation, data curation, conceptualization. **Jae‐Hwan Kwak**: supervision, project administration, funding acquisition, writing – review and editing, visualization, validation, investigation, data curation, conceptualization.

## Funding

This work was supported by the research grant of Chungbuk National University in 2022 and by the National Research Foundation of Korea (NRF) grant funded by the Korea government (MSIT) (RS‐2023‐NR076457).

## Conflicts of Interest

Y.‐M. Kwon, Y.‐S. Jung, and J.‐H. Kwak are inventors on Korean patent No. 10‐2271062−0000, which covers the pyrrolo[1,2‐*a*]pyrazine‐carboxamide analogs described in this study. Two additional inventors, Y.‐J. Kim and S.‐B. Lim, are not authors of this paper. The authors declare no other competing financial interests or personal relationships that could have appeared to influence the work reported in this paper.

## Supporting information

The ^1^H NMR and ^13^C NMR spectra of all synthesized compounds are provided in the Supporting Information.

## Data Availability

The data that supports the findings of this study are available in the supporting information of this article.
