# Supplementary material for: (S)‐Pyrrolo[1,2‐a]pyrazine‐3‐carboxamide Analogs as Dual Akt/JNK Modulators With Antiproliferative Effects in MDA‐MB‐468 Cells
Source: ChemMedChem. 2026 Aug 27;21(17):e70463. doi: 10.1002/cmdc.70463 (PMC13521716; doi:10.1002/cmdc.70463)
Supplement: Supplementary file 1 [file CMDC-21-e70463-s001.pdf]

(*S*)-Pyrrolo[1,2-*a*]pyrazine-3-carboxamide Analogues as Dual Akt/JNK Modulators with Antiproliferative Effects in MDA-MB-468 Cells

Narges Hosseininasab[a,†], Sou Hyun Kim[b,†], Ye-Mi Kwon[c,†], Jungjin Park[a], Chanhyun Jung[a], Kwanghee Lee[a], Beom-Jun Wi[a], Su-Jin Yoo[a], Soonsil Hyun[a], Young-Suk Jung[b,*], and Jae-Hwan Kwak[a,*]

[a]College of Pharmacy, Chungbuk National University, Cheongju, Chungbuk 28160, Republic of Korea

[b]College of Pharmacy, Research Institute for Drug Development, Pusan National University, Busan 46241, Republic of Korea

[c]Development Center (NDDC), Daegu-Gyeongbuk Medical Innovation Foundation (KMEDIhub), Daegu 41061, Republic of Korea

[†]These authors contributed equally to this work.

*Corresponding authors:

Prof. Young-Suk Jung, College of Pharmacy, Research Institute for Drug Development, Pusan National University, Busan 46241, Republic of Korea. Email: youngjung@pusan.ac.kr;

Prof. Jae-Hwan Kwak, College of Pharmacy, Chungbuk National University, Cheongju, Chungbuk 28160, Republic of Korea. Email: jhkwak@chungbuk.ac.kr

Table 1. Cell viability at 10 and 30 µM, GI_{50} value, and selectivity index (SI) of (*S*)-1-oxo-1,2,3,4-tetrahydropyrrolo[1,2-*a*]pyrazine-3-carboxamide analogs (**A1**–**F5**) against the MCF-7 and MDA-MB-468 cell lines.

Compounds	% Cell viability at 10 µM		% Cell viability at 30 µM		GI_{50} value[a] (µM)		
	MCF-7	MDA-MB-468	MCF-7	MDA-MB-468	MCF-7	MDA-MB-468	SI
**Ctrl**	100±.3.2	100±1.3	100±2.8	100±3.2	-	-	-
**A1**	92.3±4.6	94.6±0.8	89.8±1.6	92.2±1.4	> 30	> 30	-
**A2**	90.5±3.3	93.7±0.7	89.4±4.6	87.7±5.4	> 30	> 30	-
**A3**	96.3±0.8	93.7±6.5	95.0±2.7	92.1±4.6	> 30	> 30	-
**A4**	89.6±5.8	92.1±1.5	86.3±3.2	81.1±9.8	> 30	> 30	-
**A5**	93.2±2.2	95.9±4.7	90.6±8.0	93.4±5.0	> 30	> 30	-
**B1**	68.8±2.0	22.3±0.1	62.6±2.4	19.0±0.8	> 30	2.8	> 10.7
**B2**	70.9±3.4	22.4±1.1	72.6±2.5	22.5±1.3	> 30	7.1	> 4.2
**B3**	70.6±5.5	27.5±1.1	61.4±5.1	19.4±0.2	> 30	4.7	> 6.4
**B4**	76.6±2.5	37.4±1.2	78.9±2.6	24.1±1.0	> 30	8.4	> 3.6
**B5**	69.5±2.3	25.6±0.5	56.5±2.3	17.1±0.5	> 30	6.0	> 5.0
**C1**	81.7±9.1	70.4±4.9	80.3±4.0	27.4±0.9	> 30	19.5	> 1.5
**C2**	90.2±0.9	78.0±1.8	71.6±3.3	40.9±15.0	> 30	25	> 1.2
**C3**	88.3±4.8	86.2±1.7	85.2±1.3	45.0±4.7	> 30	27.6	> 1.1
**C4**	93.2±4.4	86.3±2.7	92.3±3.0	65.2±7.5	> 30	> 30	-
**C5**	87.8±1.1	76.5±4.9	77.8±5.2	36.4±1.3	> 30	23.2	> 1.3
**D1**	71.4±4.0	65.3±1.9	44.1±4.0	10.0±1.2	25.6	15.5	1.7
**D2**	77.4±4.3	63.7±4.1	45.7±2.1	21.1±1.1	27.3	16.4	1.7
**D3**	85.0±4.2	81.9±2.6	75.5±3.2	35.5±1.8	> 30	23.7	> 1.3
**D4**	83.4±5.6	82.1±2.8	64.6±2.9	33.1±3.5	> 30	23.1	> 1.3
**D5**	85.8±4.3	84.6±3.2	76.2±1.9	68.2±2.2	> 30	> 30	-
**E1**	69.4±4.4	60.8±0.8	48.7±2.2	13.1±2.5	28.7	14.5	2.0
**E2**	74.0±1.0	71.9±3.1	48.6±3.0	32.7±2.7	28.8	21.2	1.4
**E3**	83.2±6.3	75.9±1.3	70.9±1.0	30.4±4.1	> 30	21.3	> 1.4
**E4**	78.3±5.1	72.3±5.8	54.6±3.8	19.0±2.6	> 30	25.1	> 1.2
**E5**	73.4±3.6	60.8±7.4	48.5±6.0	15.2±2.4	28.8	14.8	2.0
**F1**	85.1±4.6	72.6±1.3	67.6±4.7	34.3±1.5	> 30	21.8	> 1.4
**F2**	83.0±5.7	69.3±2.4	77.4±4.4	44.6±3.0	> 30	25.6	> 1.2
**F3**	94.8±6.9	89.8±4.0	92.9±2.6	92.1±7.6	> 30	> 30	-
**F4**	89.6±1.4	84.6±5.9	81.4±4.7	42.2±4.4	> 30	26.3	> 1.1
**F5**	77.8±1.0	77.3±1.8	76.4±8.5	41.5±2.0	> 30	25.2	> 1.2
**Ref 2**	47.1±2.7	28.0±0.8	30.7±1.7	20.7±0.8	9.6	7.6	1.26
**Gefitinib**	81.7±6.8	69.5±1.3	79.1±4.9	9.7±1.1	> 30	16.5	> 1.8

[a] GI_{50} values are reported as the mean of four experiments and correspond to the agent's concentration that causes a 50% decrease in net cell growth.

1. ^{1}H and ^{13}C NMR spectra

^{1}H and ^{13}C NMR: *Methyl (1H-pyrrole-2-carbonyl)-L-serinate* (**3**)

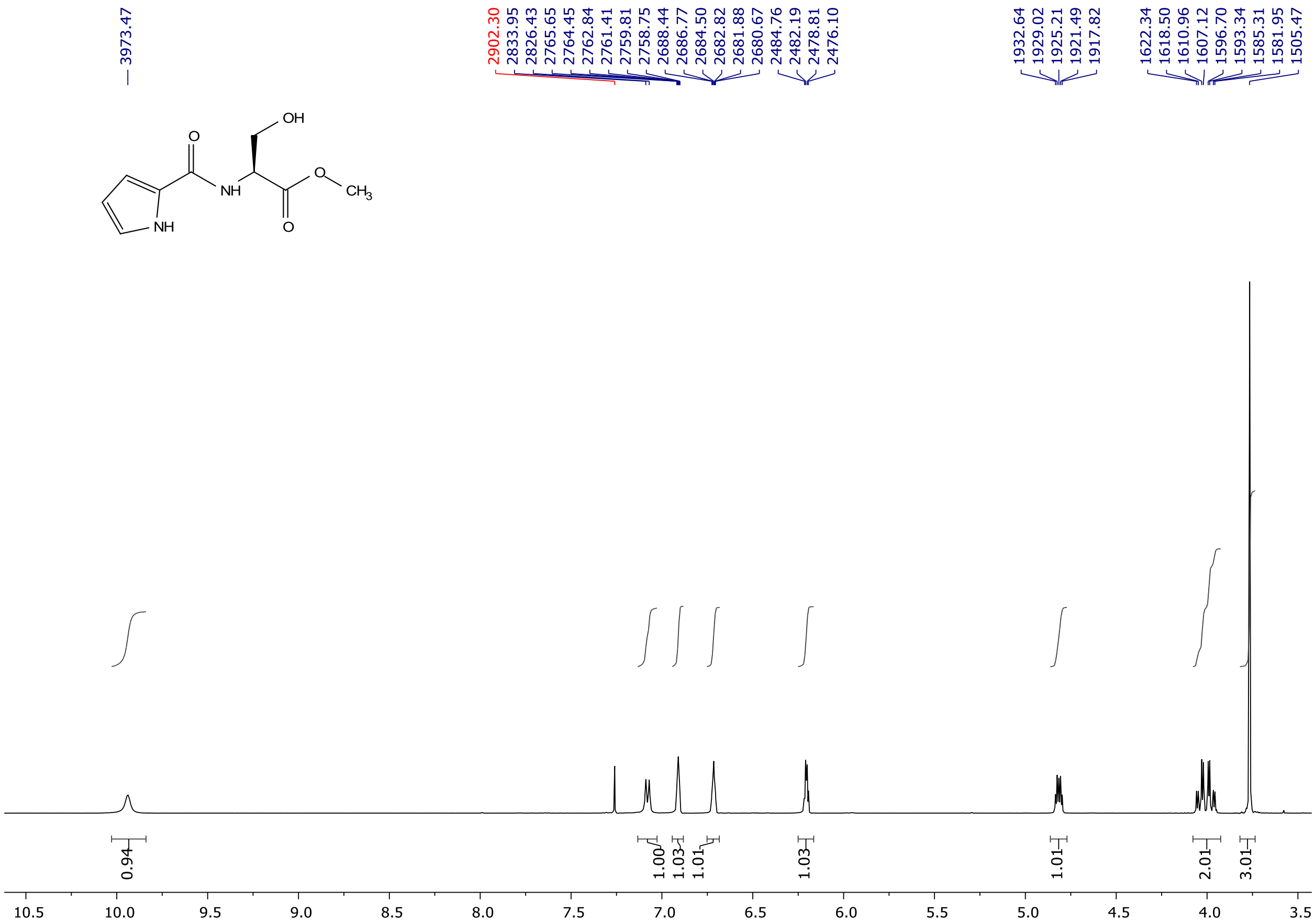

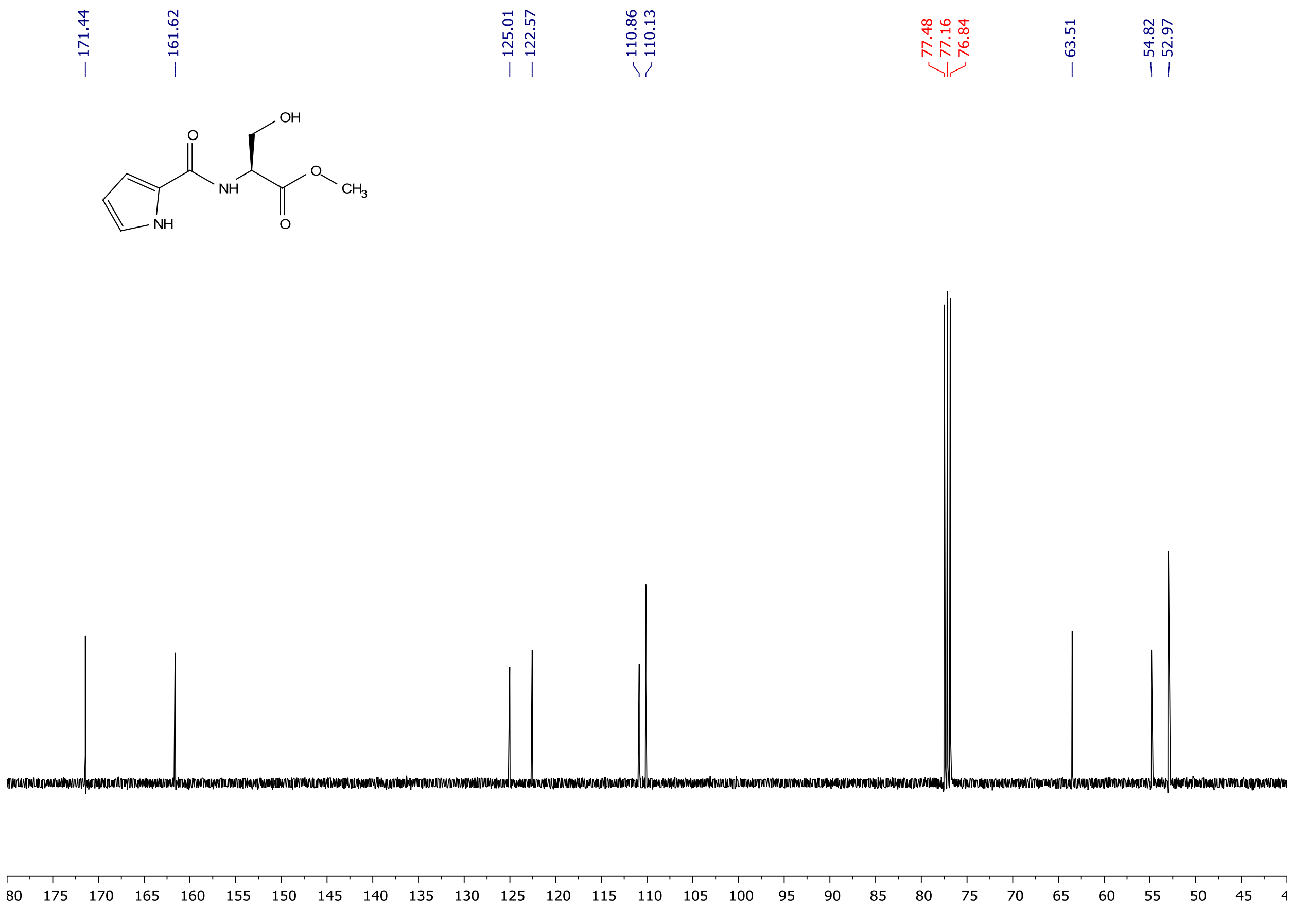

^{1}H and ^{13}C NMR: *Methyl (S)-1-oxo-1,2,3,4-tetrahydropyrrolo[1,2-a]pyrazine-3-carboxylate* (**4**)

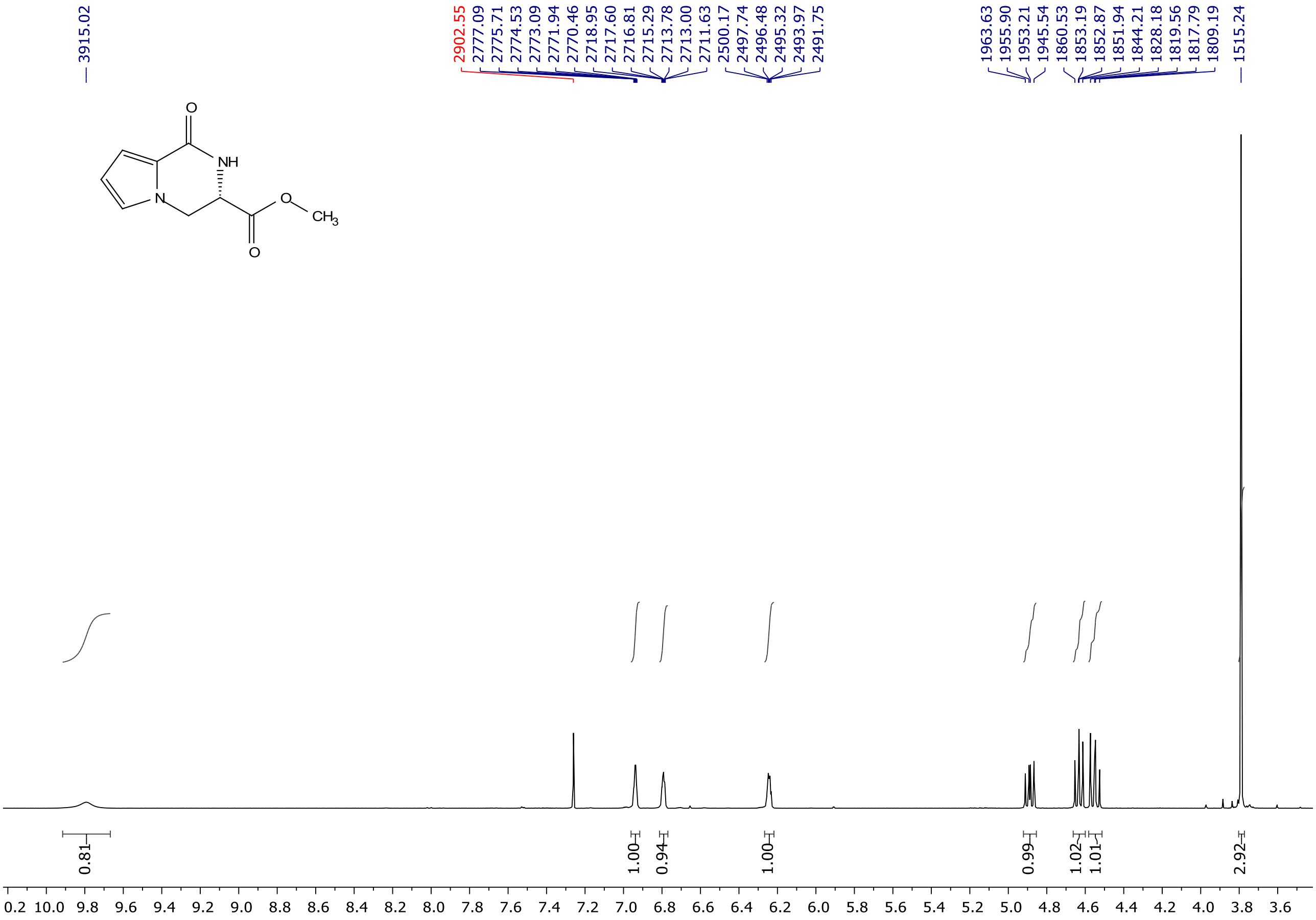

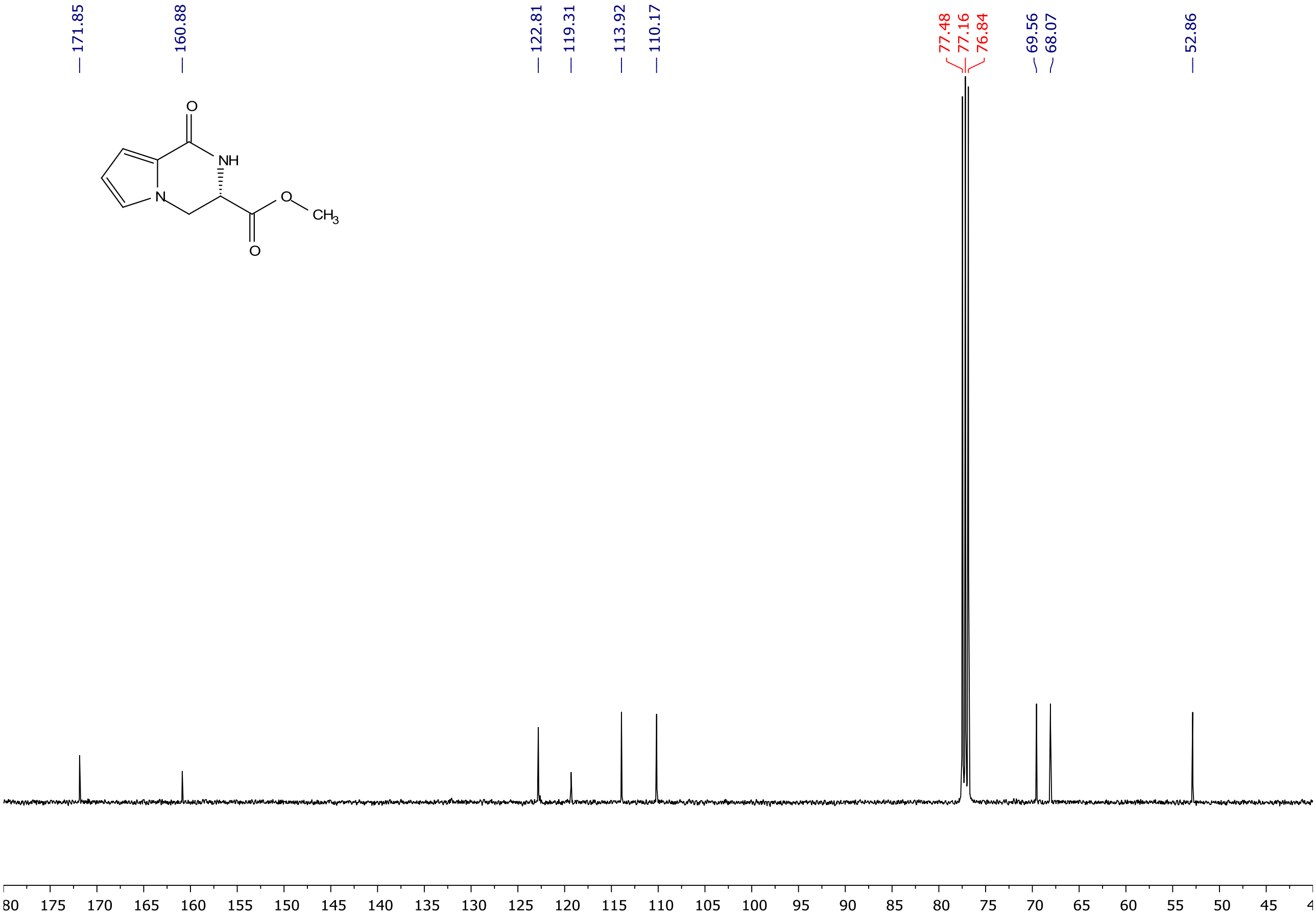

^{1}H and ^{13}C NMR: *Methyl (S)-2-benzyl-1-oxo-1,2,3,4-tetrahydropyrrolo[1,2-a]pyrazine -3-carboxylate (**5b**)*

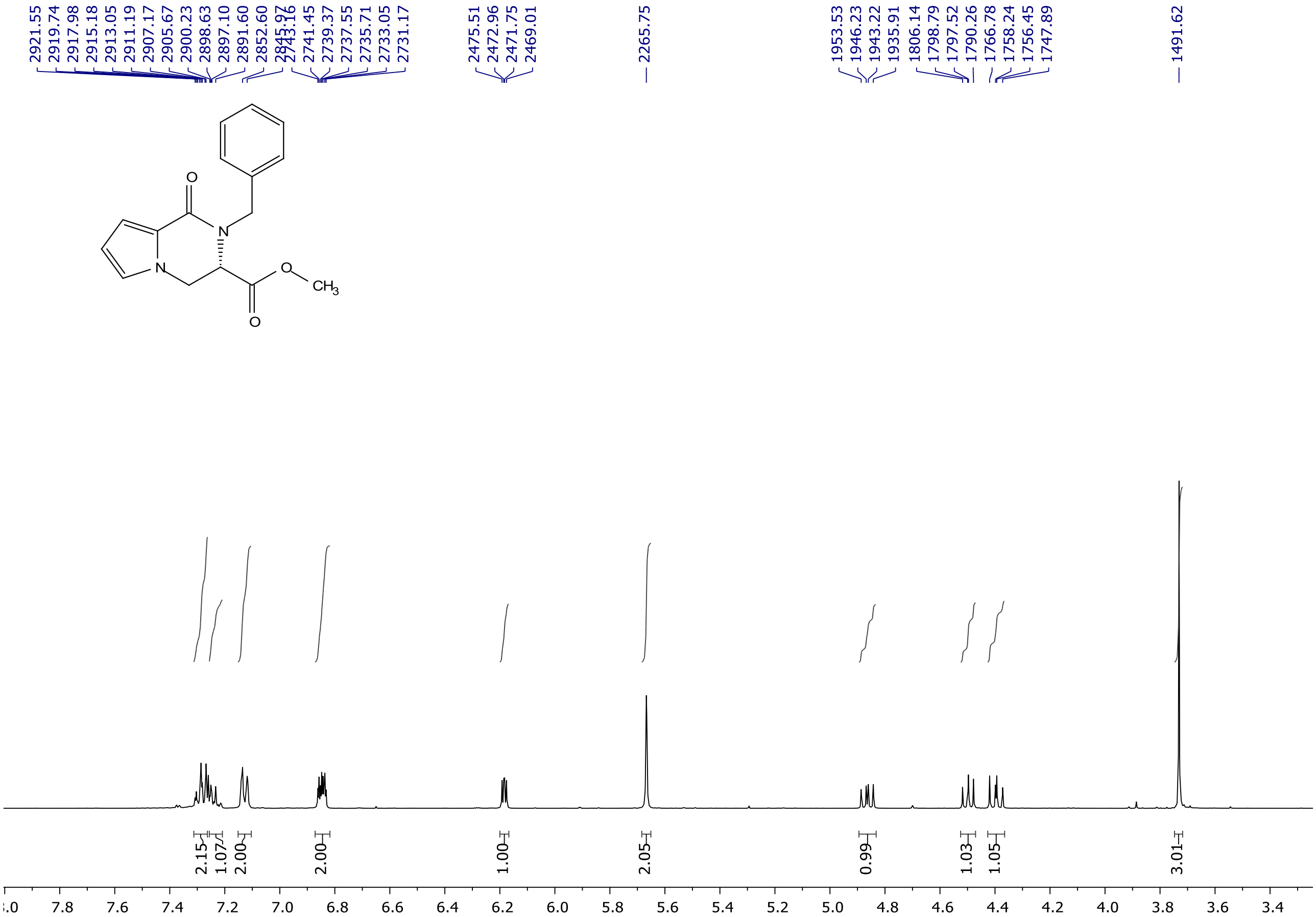

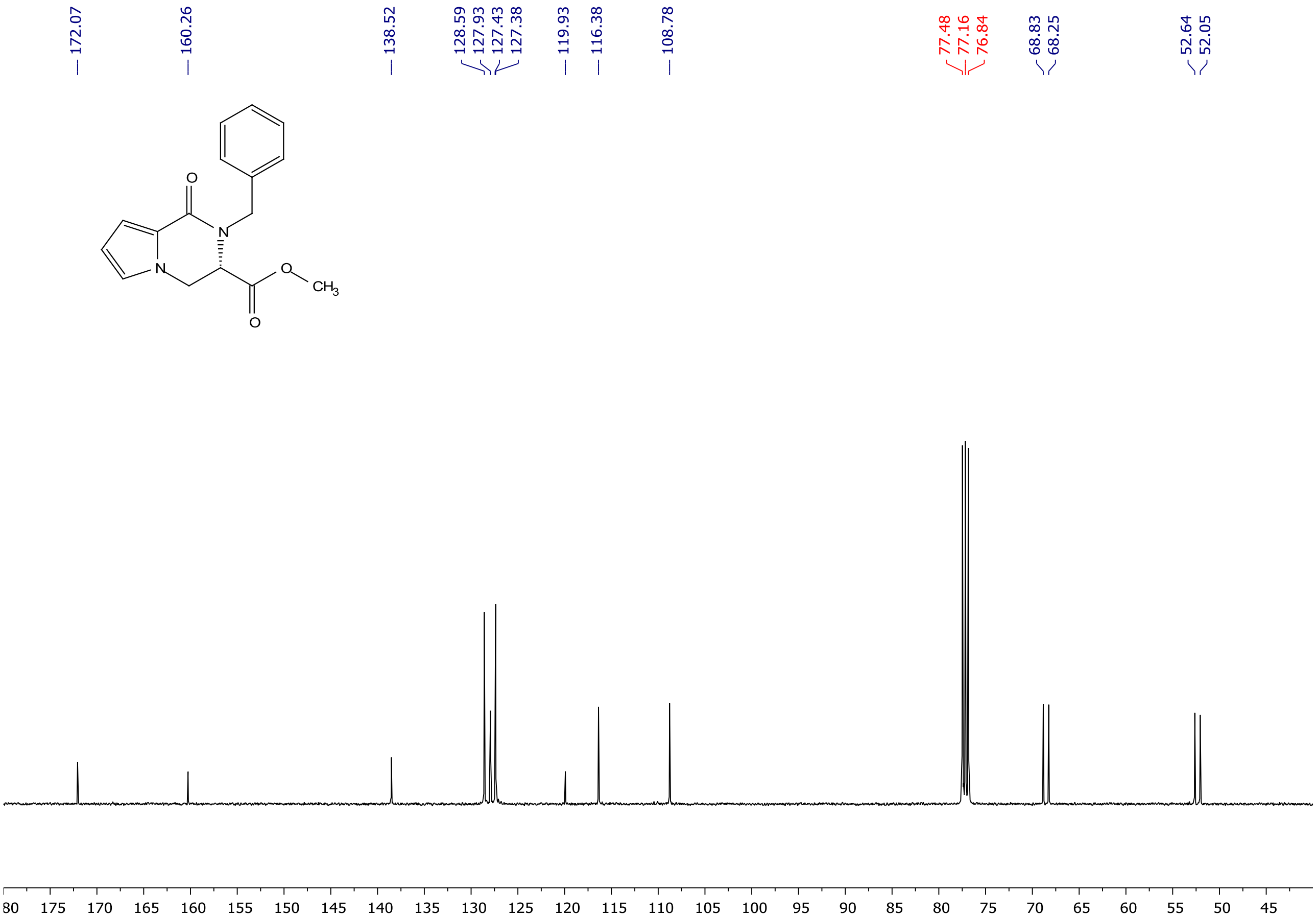

^{1}H and ^{13}C NMR: *Methyl (S)-2-(4-cyanobenzyl)-1-oxo-1,2,3,4-tetrahydropyrrolo[1,2-a]pyrazine -3-carboxylate* (***5c***)

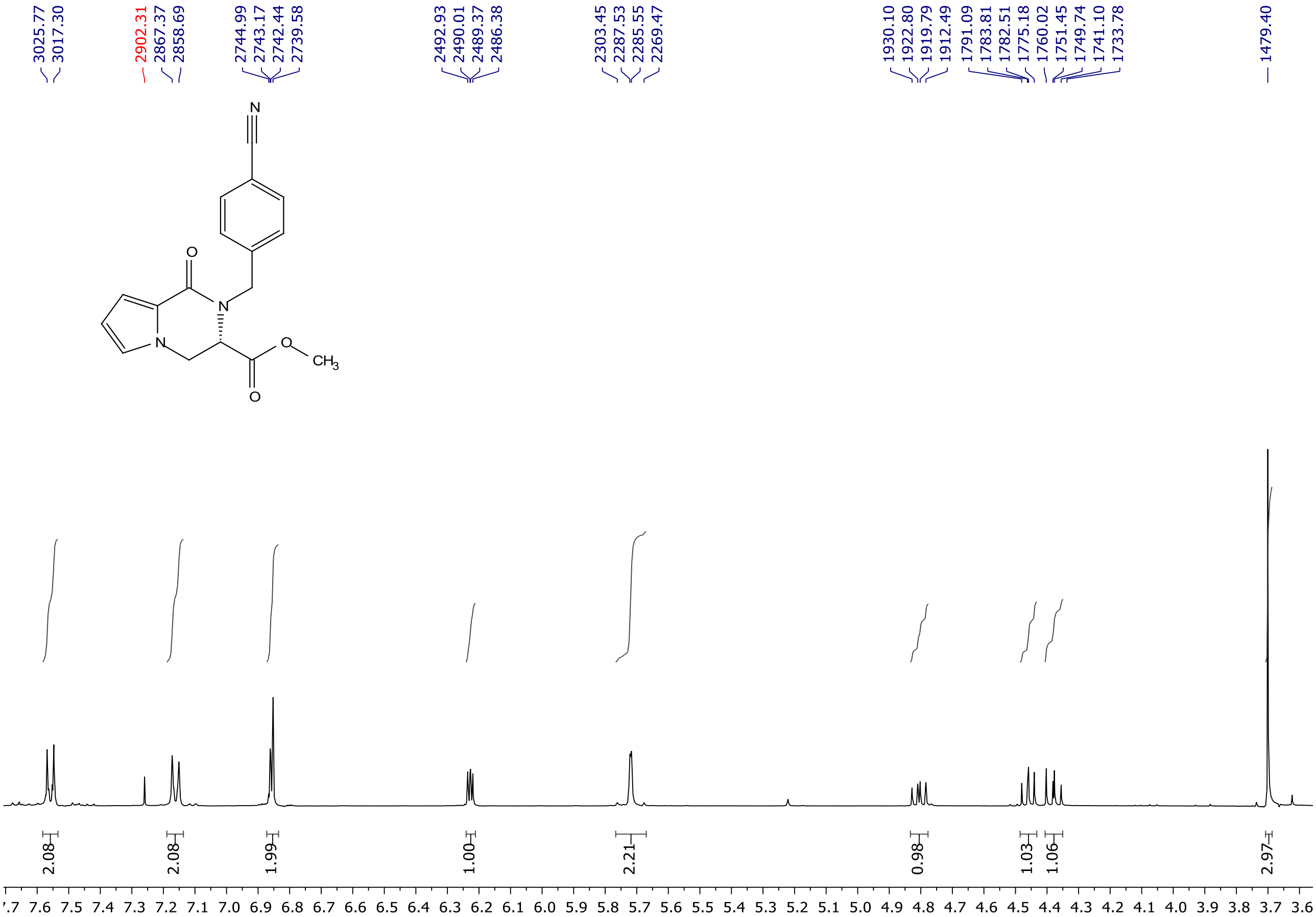

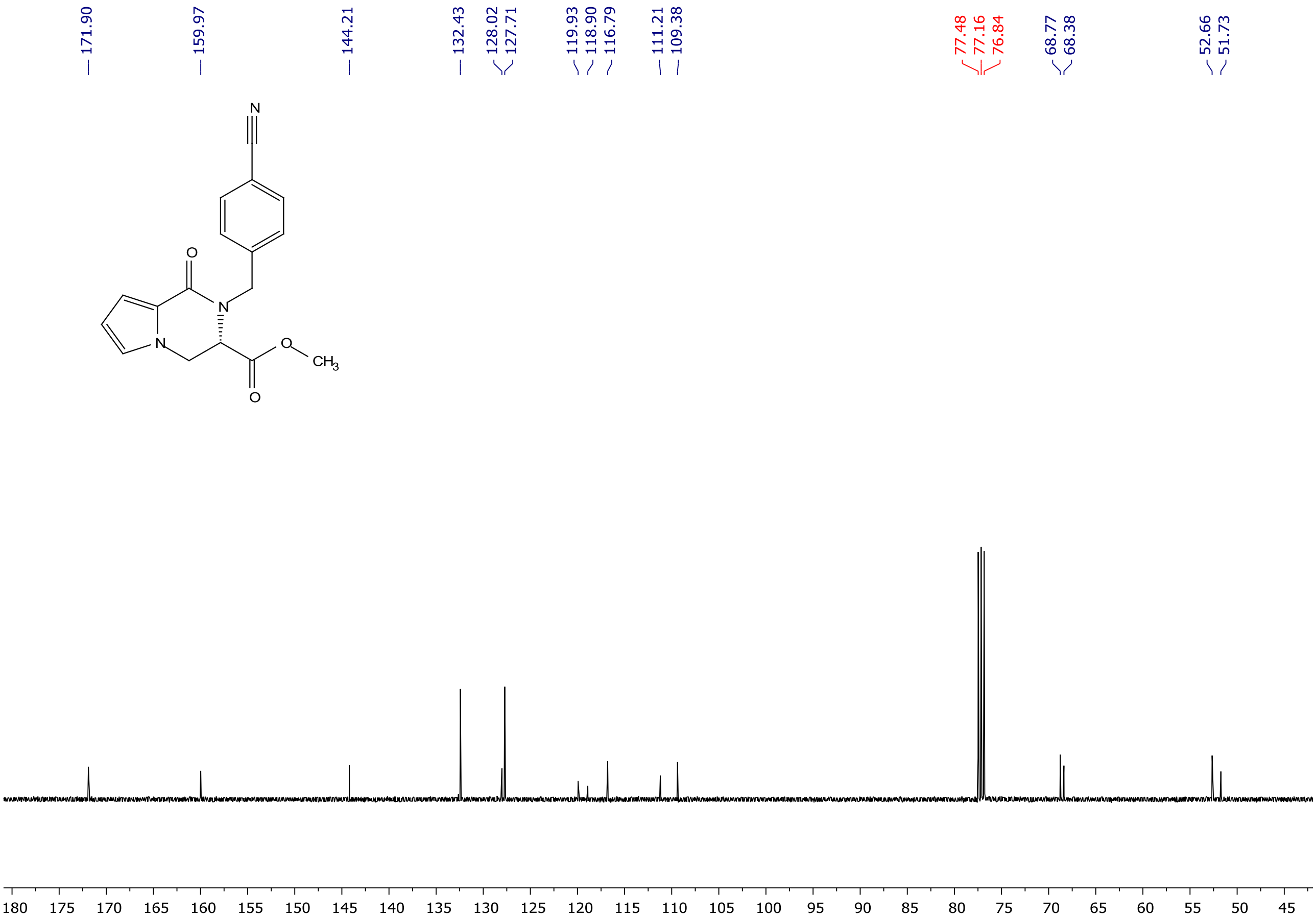

^{1}H and ^{13}C NMR: *Methyl (S)-1-oxo-2-(4-(trifluoromethyl)benzyl)-1,2,3,4-tetrahydropyrrolo[1,2-a]pyrazine -3-carboxylate* (***5d***)

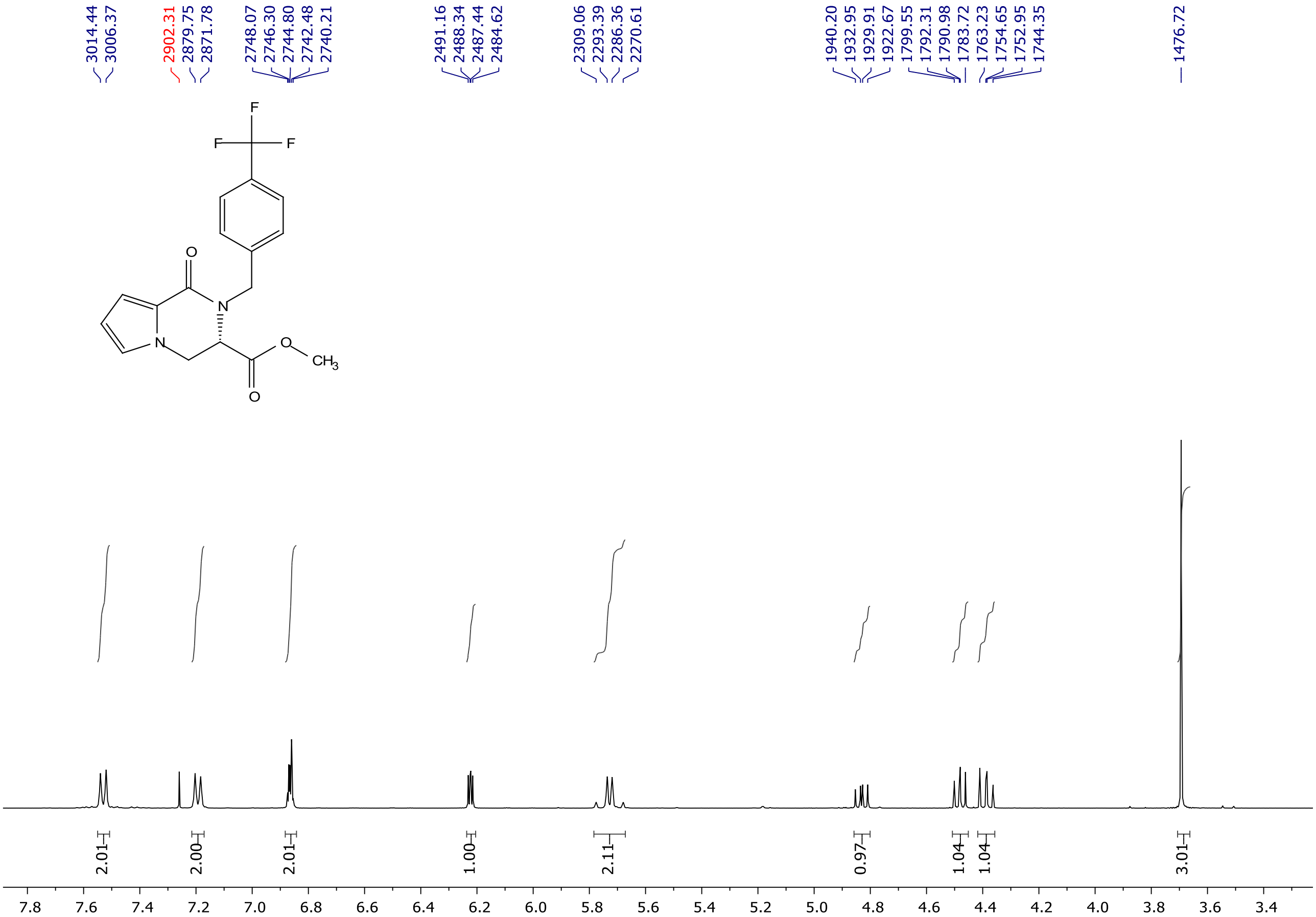

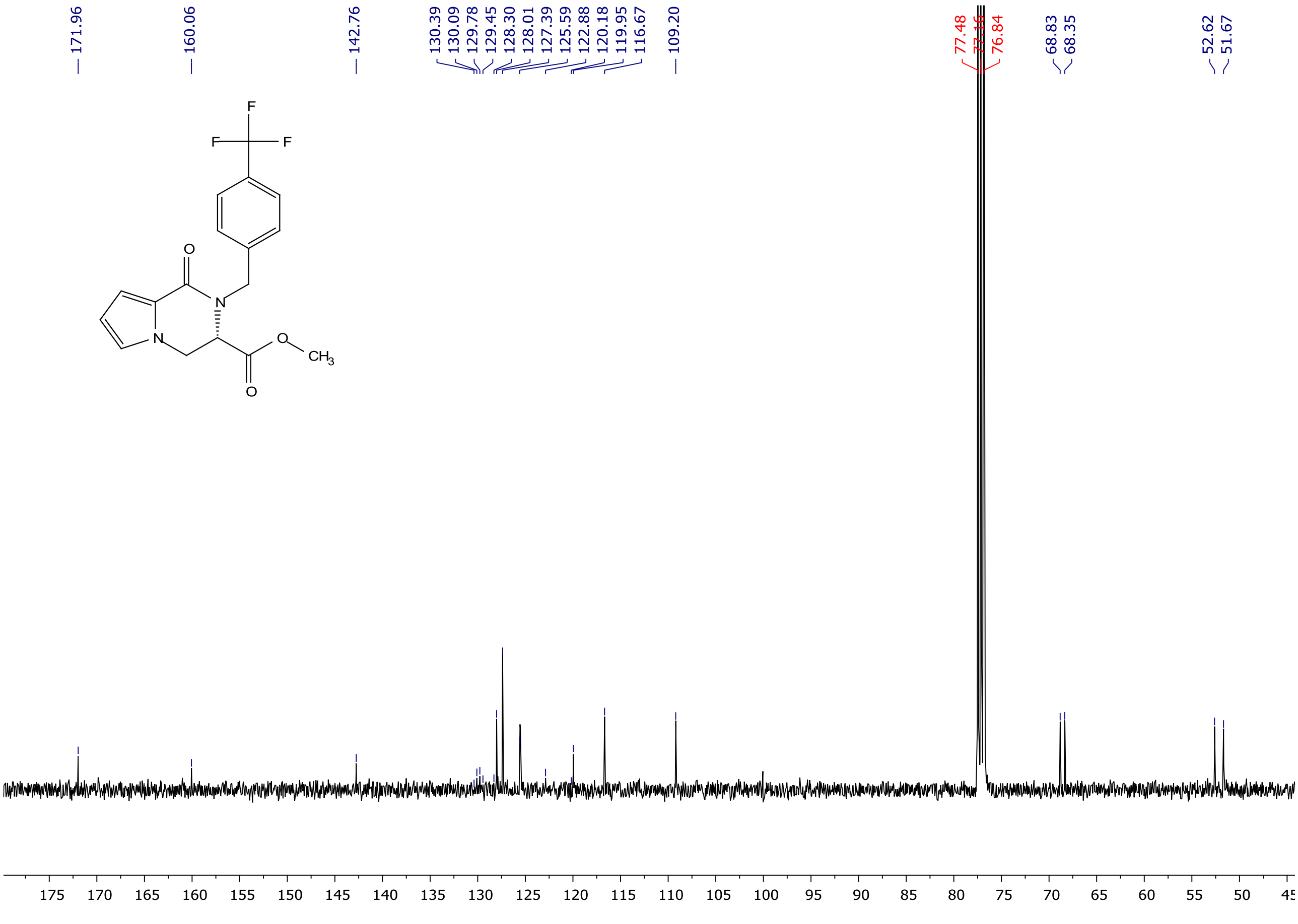

^{1}H and ^{13}C NMR: *Methyl (S)-1-oxo-2-(4-(trifluoromethoxy)benzyl)-1,2,3,4-tetrahydropyrrolo[1,2-a]pyrazine -3-carboxylate (**5e**)*

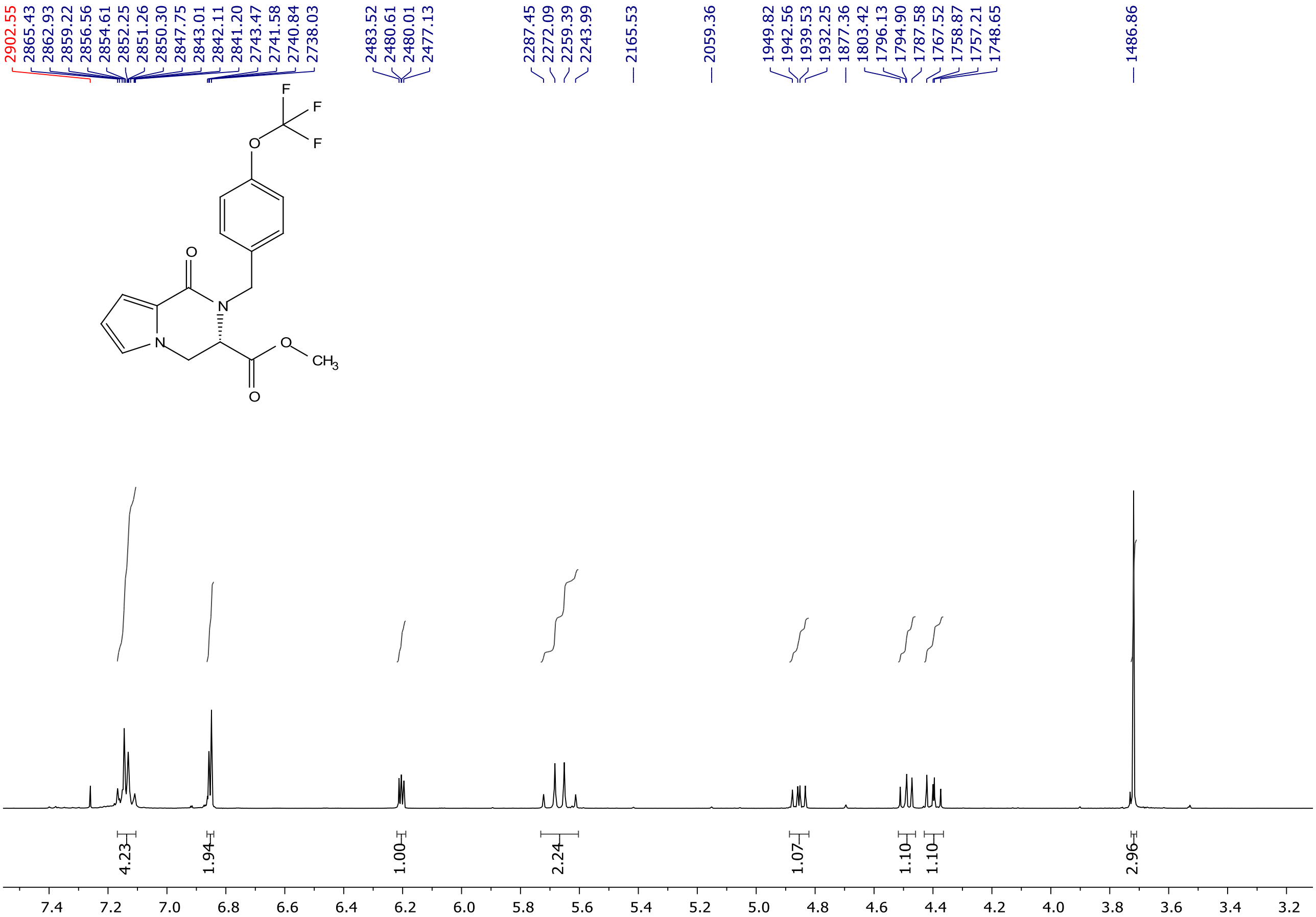

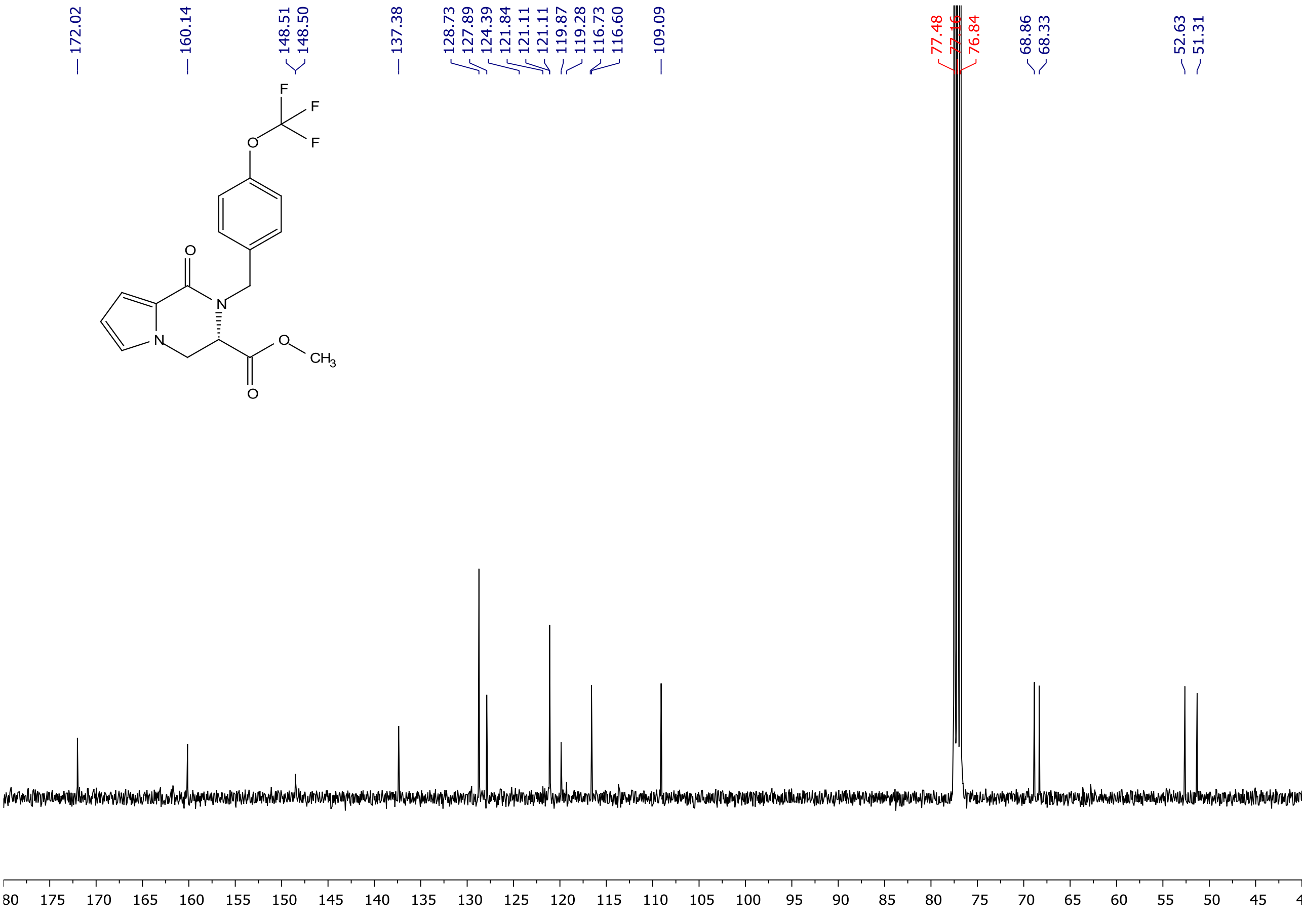

^{1}H and ^{13}C NMR: *Methyl (S)-2-(4-nitrobenzyl)-2-benzyl-1-oxo-1,2,3,4-tetrahydropyrrolo[1,2-a]pyrazine -3-carboxylate (**5f**)*

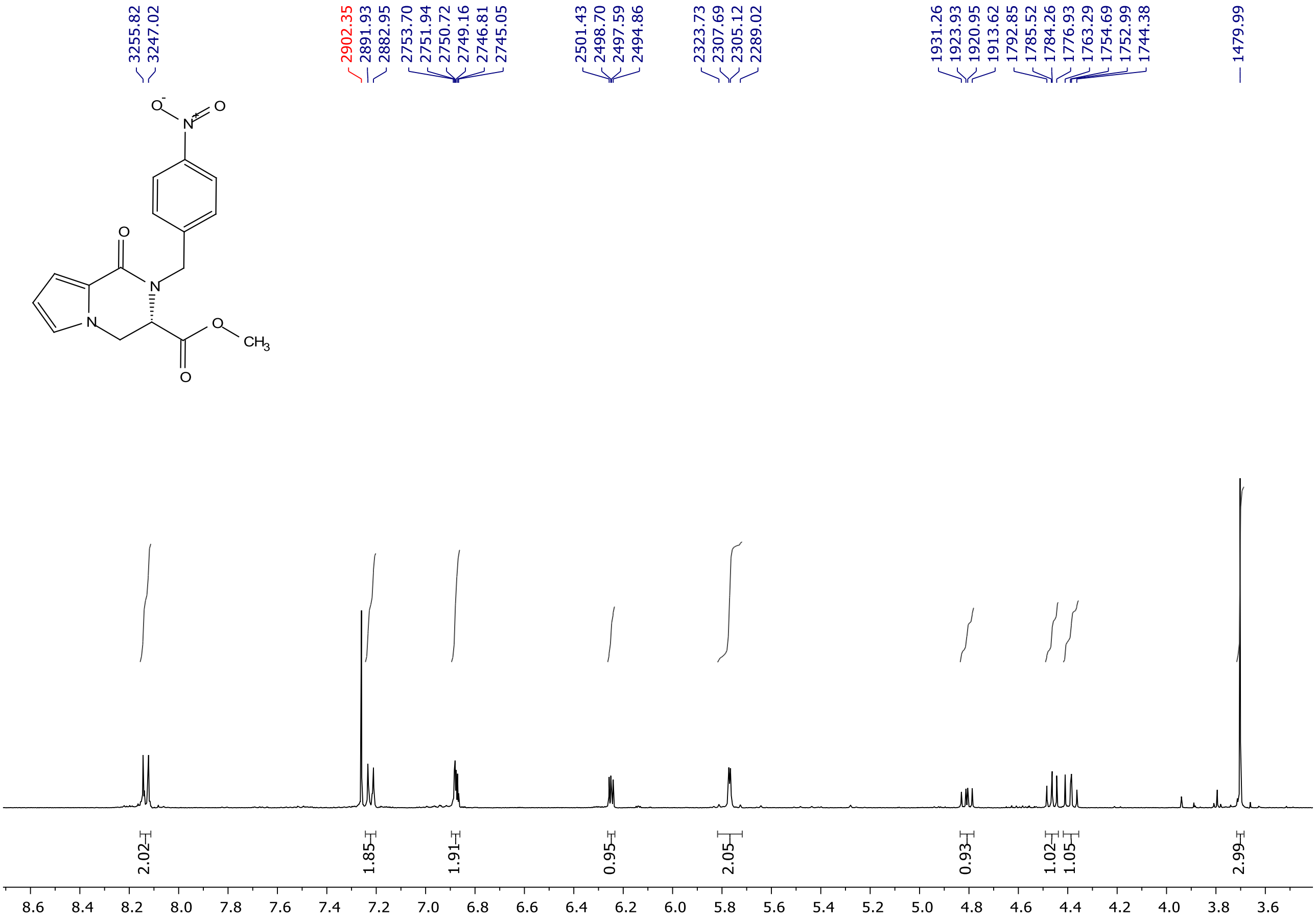

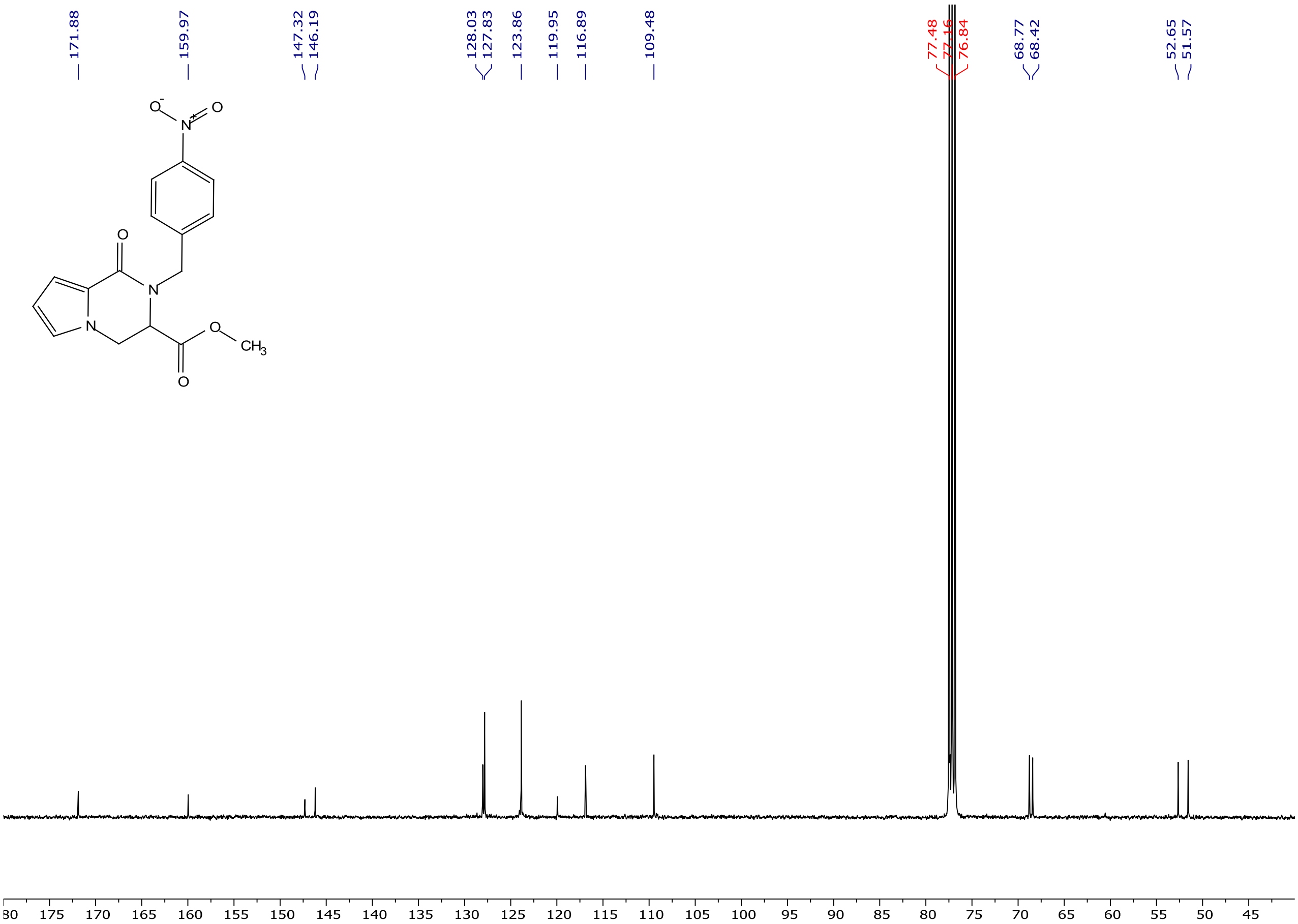

^{1}H and ^{13}C NMR: *(S)-N-Benzyl-1-oxo-1,2,3,4-tetrahydropyrrolo[1,2-a]pyrazine-3-carboxamide* (***A1***)

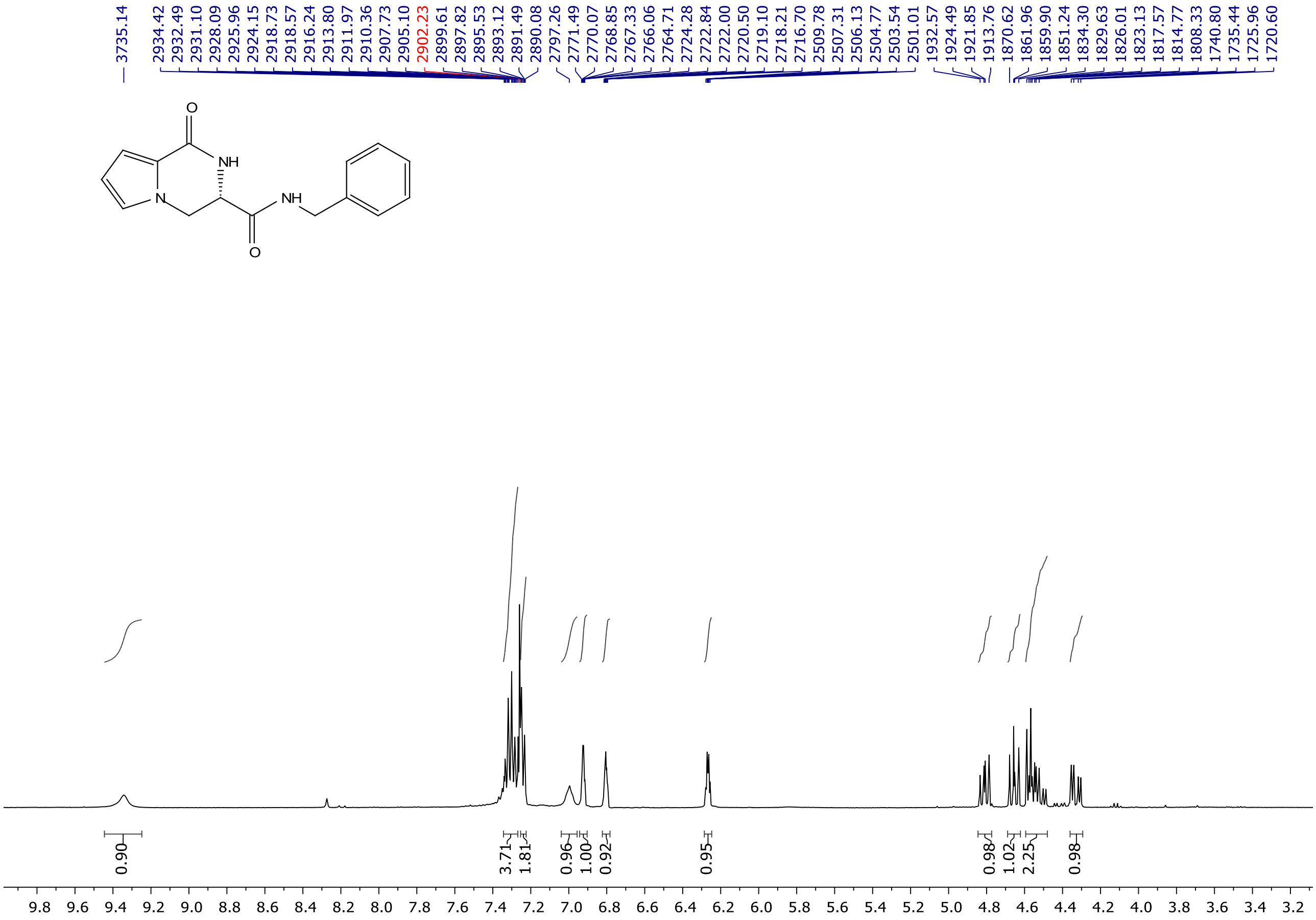

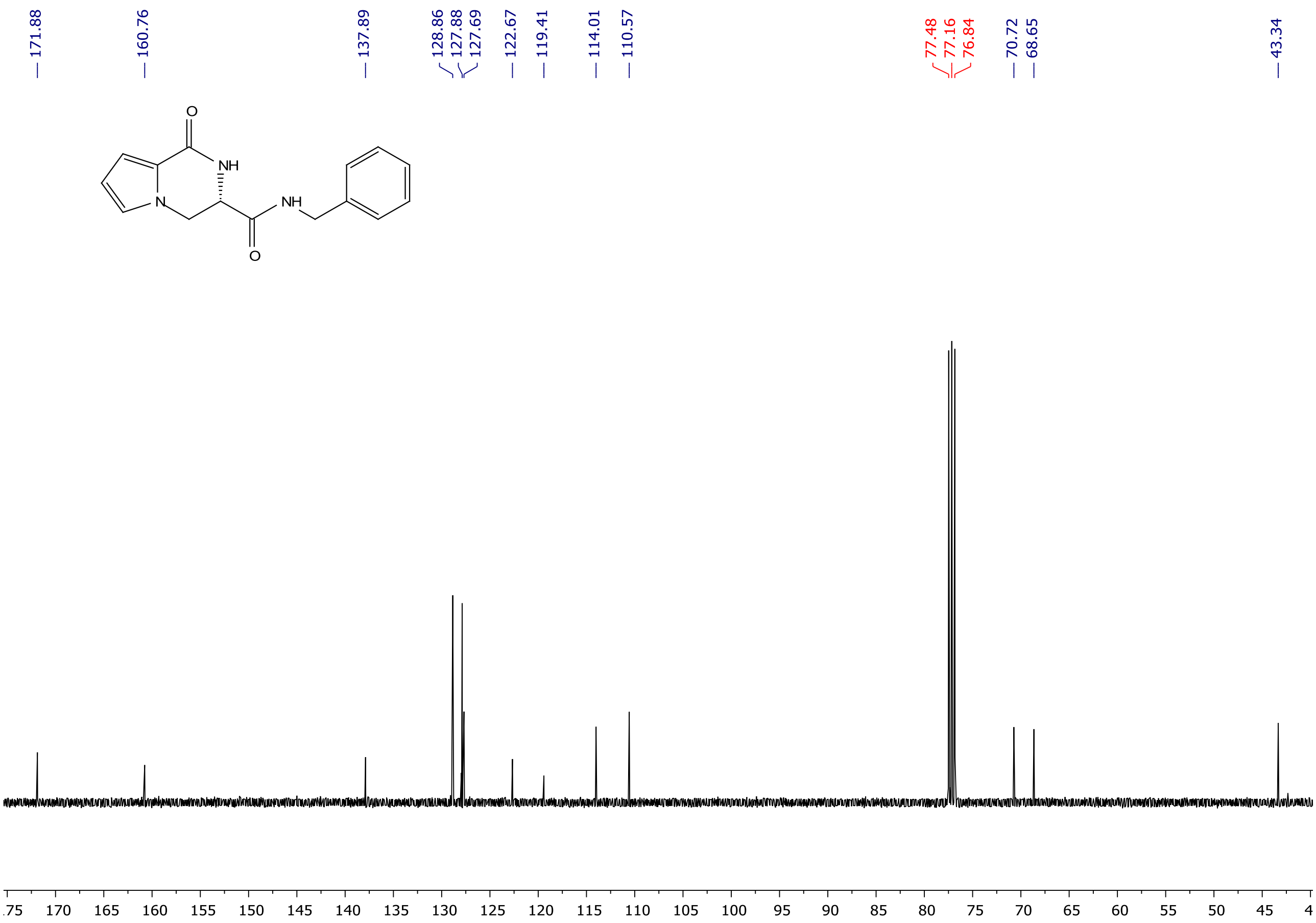

^{1}H and ^{13}C NMR: *(S)-N-(3-Chlorobenzyl)-1-oxo-1,2,3,4-tetrahydropyrrolo[1,2-a]pyrazine-3-carboxamide* (***A2***)

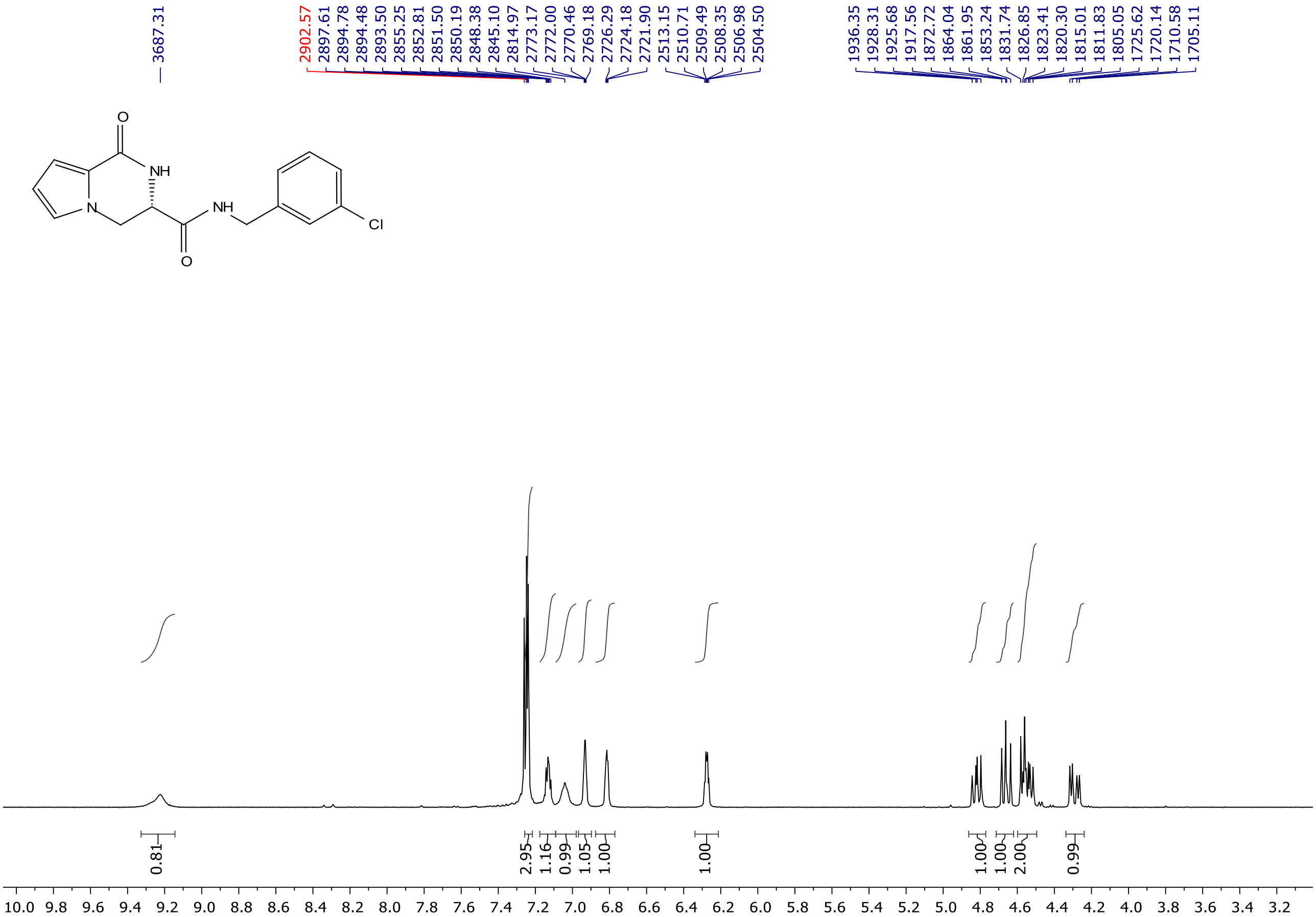

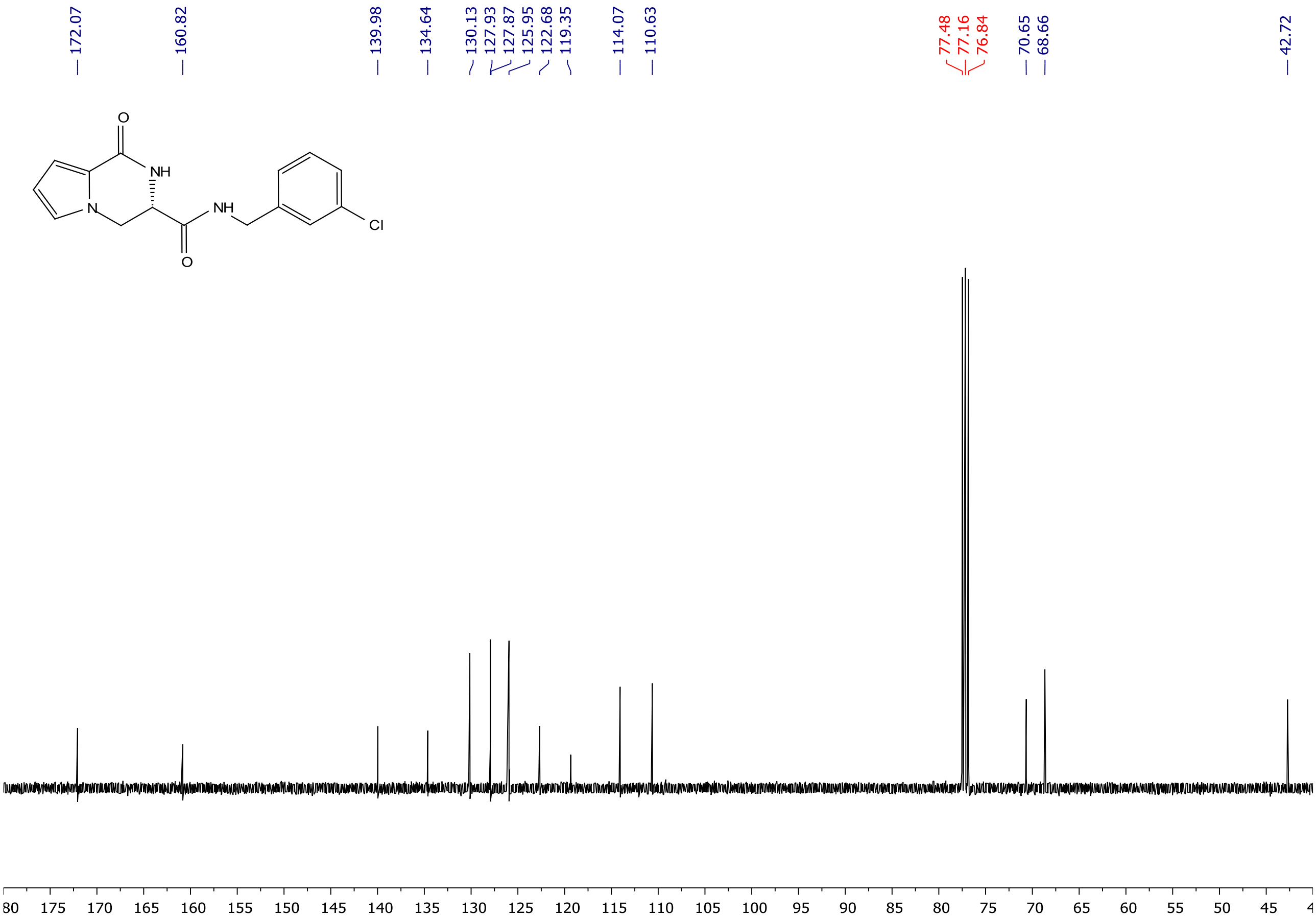

^{1}H and ^{13}C NMR: *(S)-N-(Furan-2-ylmethyl)-1-oxo-1,2,3,4-tetrahydropyrrolo[1,2-a]pyrazine-3-carboxamide (**A3**)*

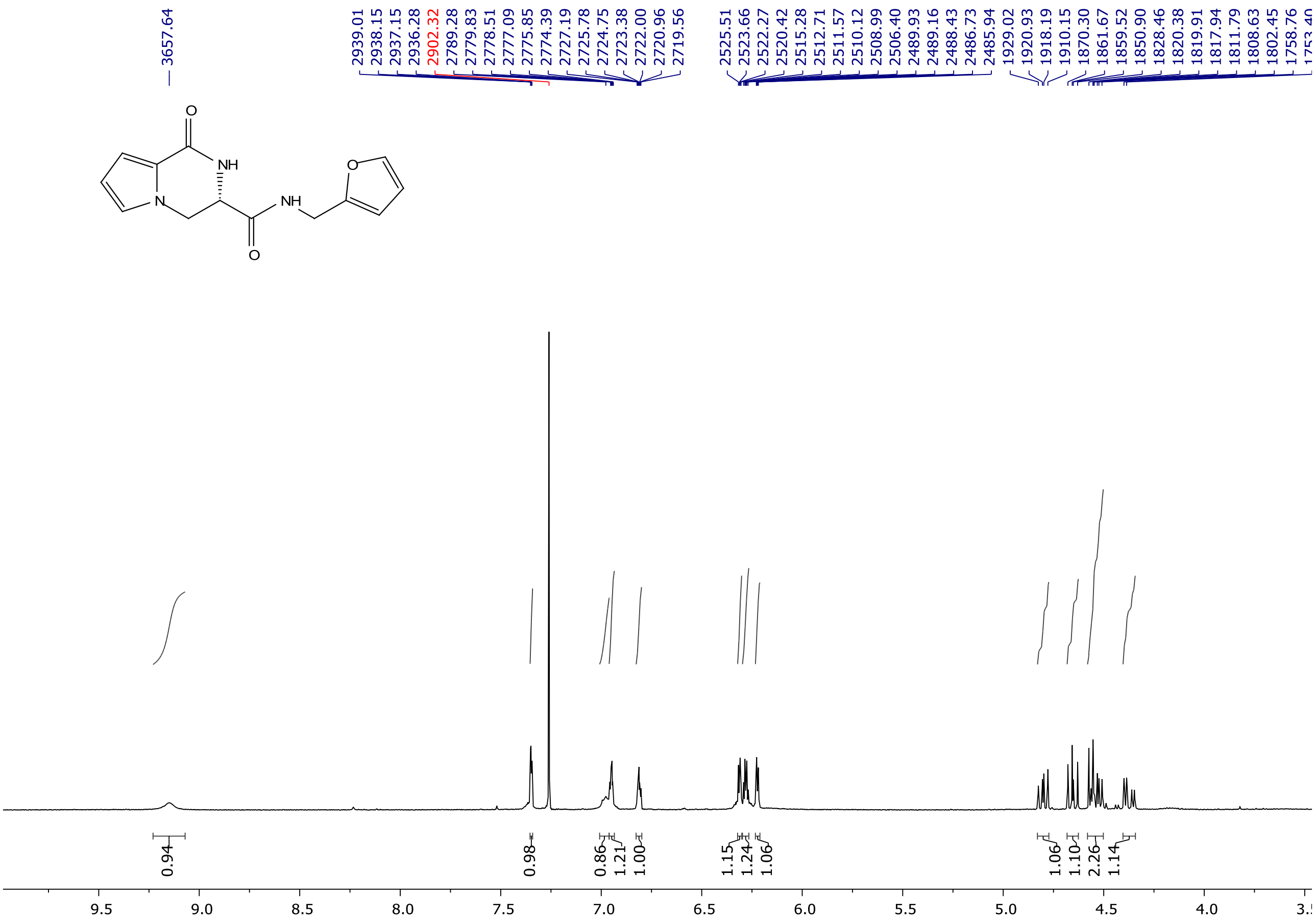

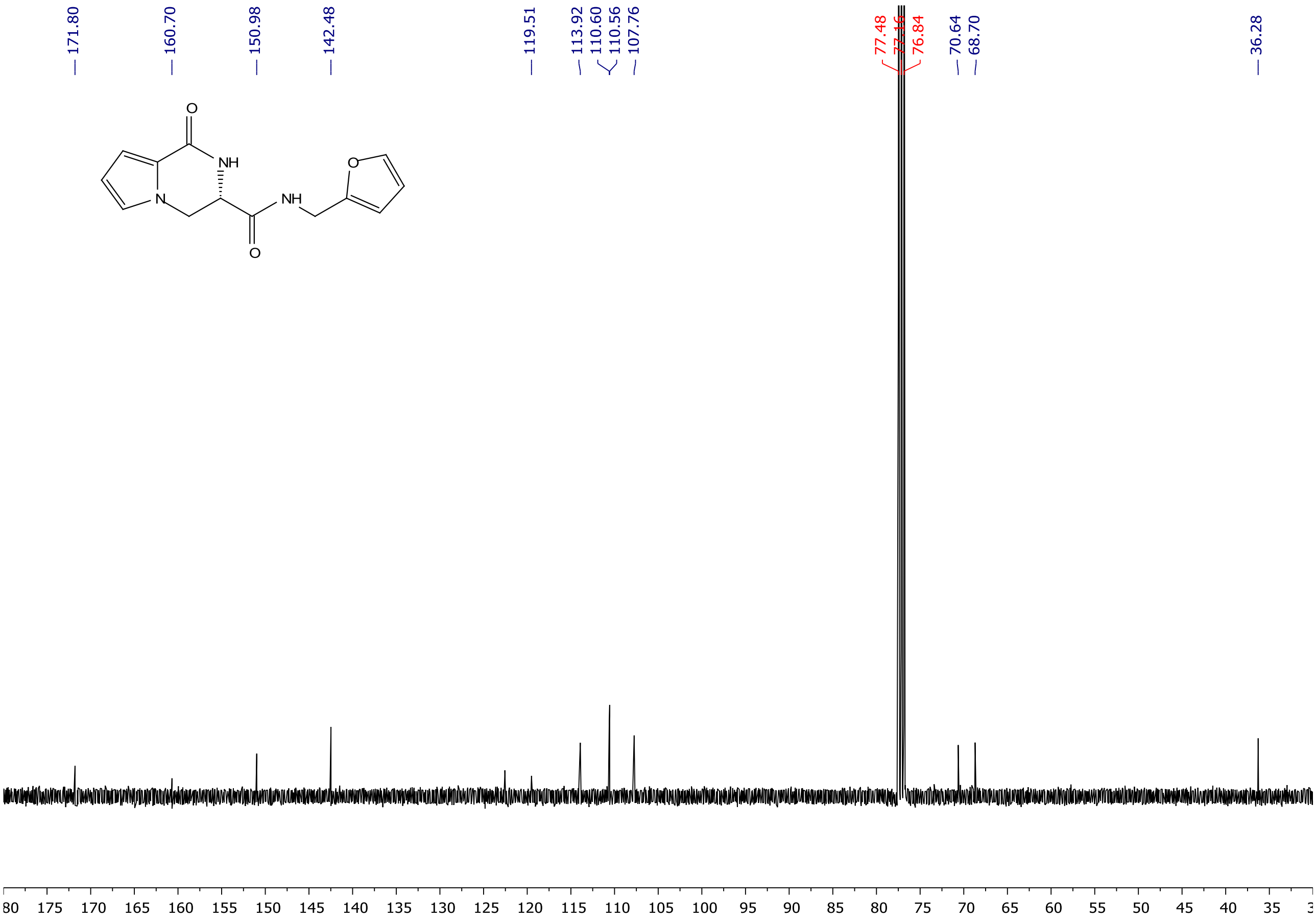

^{1}H and ^{13}C NMR: *(S)-N-((5-Methylfuran-2-yl)methyl)-1-oxo-1,2,3,4-tetrahydropyrrolo[1,2-a]pyrazine-3-carboxamide* (***A4***)

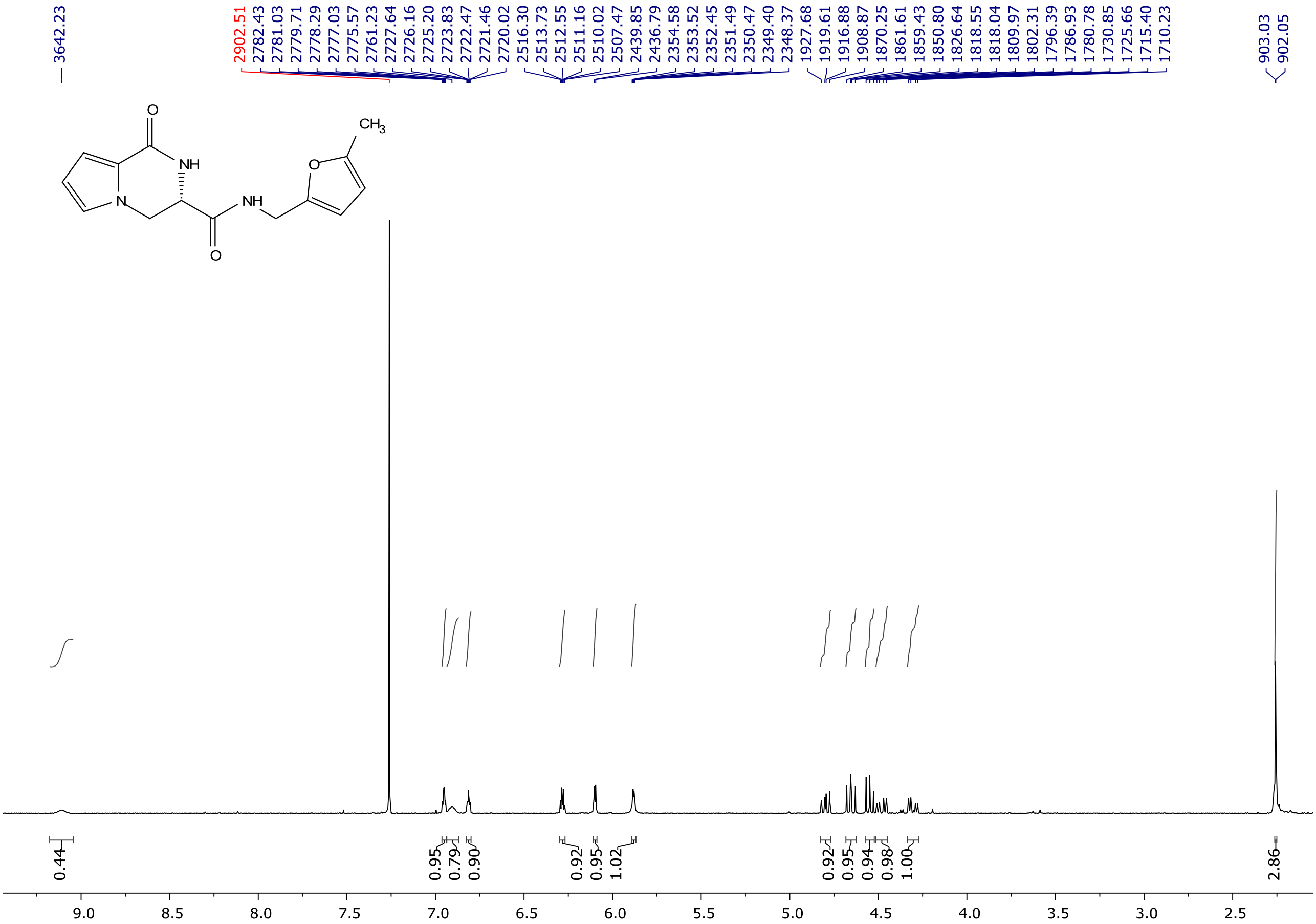

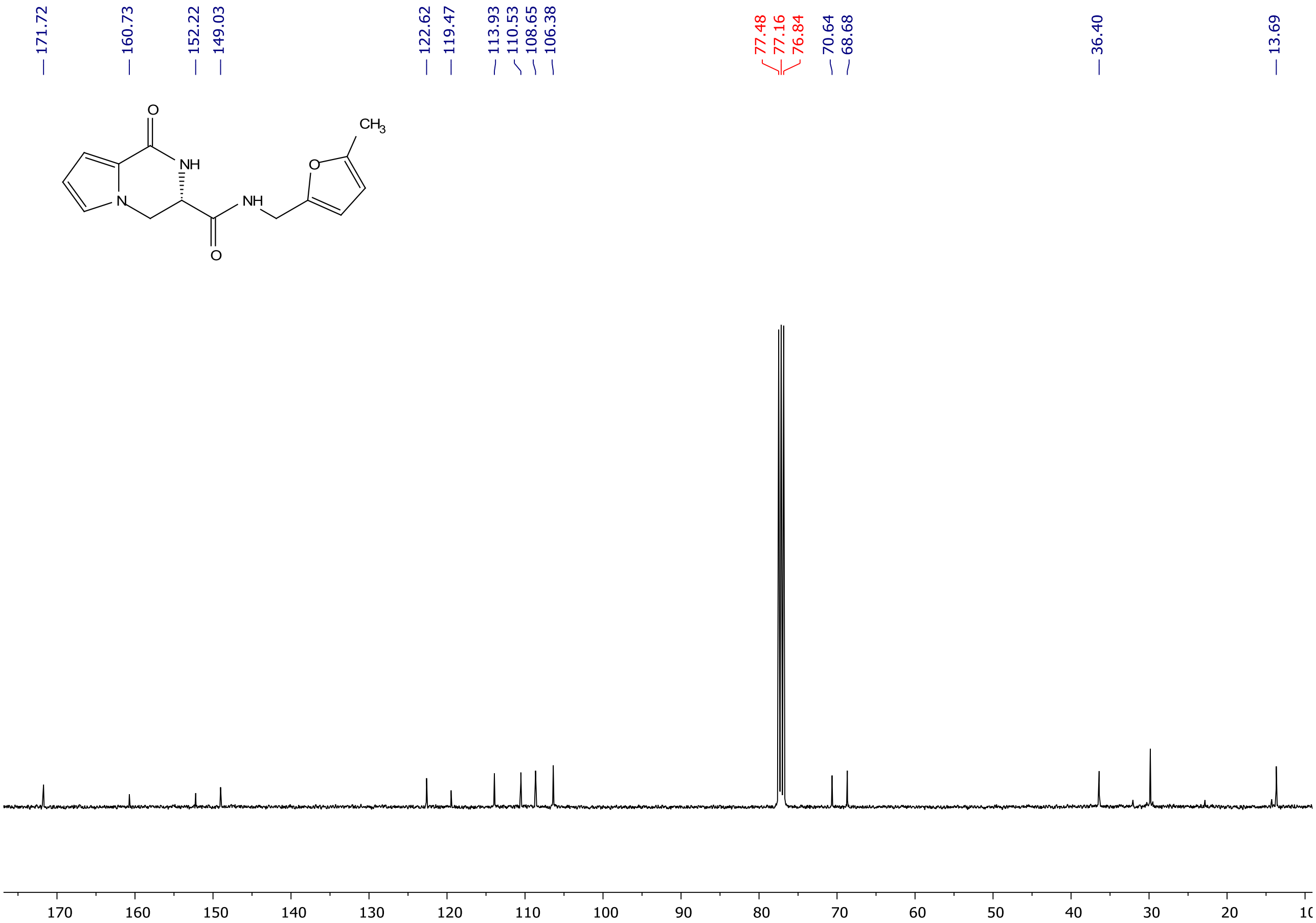

^{1}H and ^{13}C NMR: *(S)-N-(Thiophen-2-ylmethyl)-1-oxo-1,2,3,4-tetrahydropyrrolo[1,2-a]pyrazine-3-carboxamide* (***A5***)

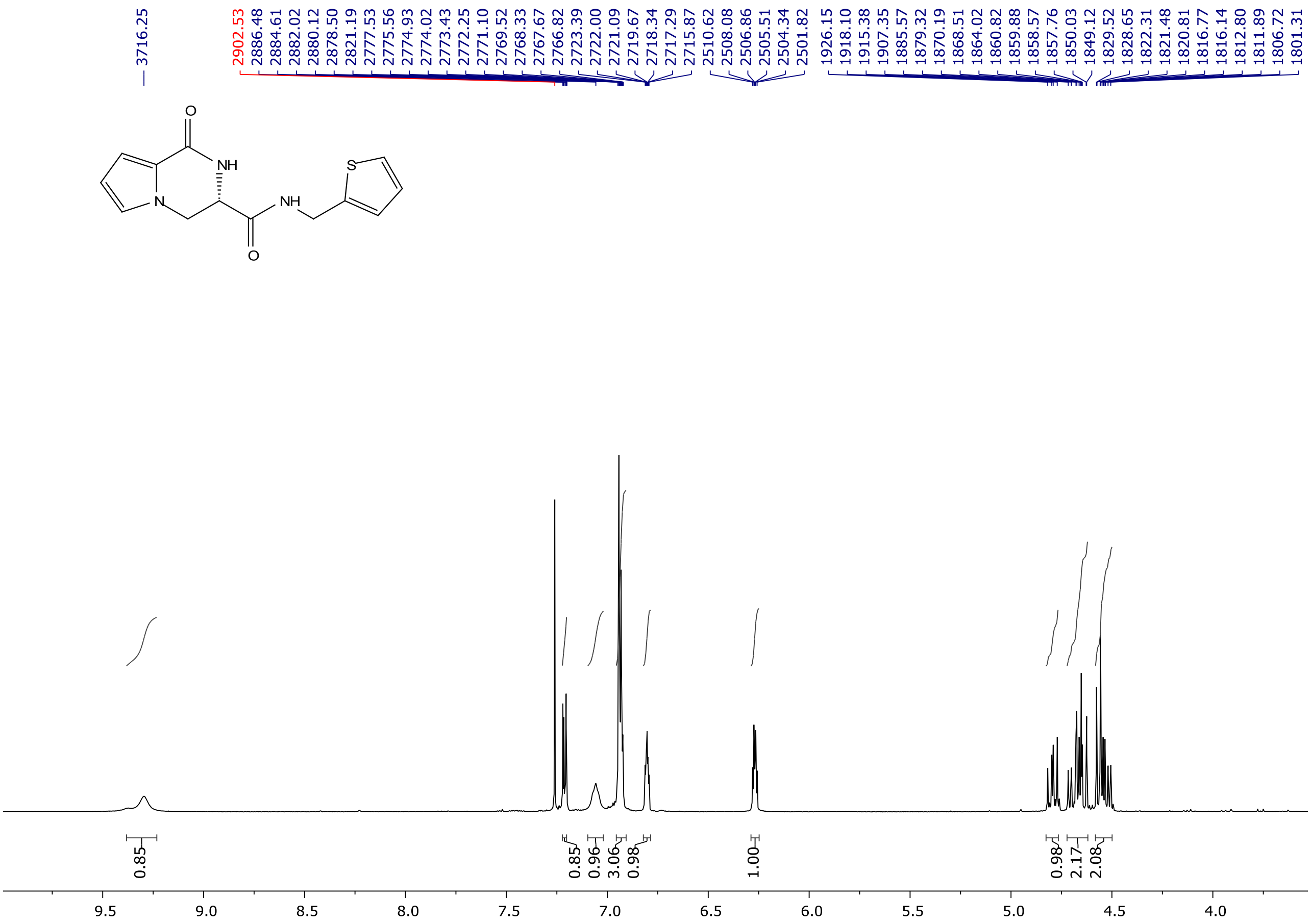

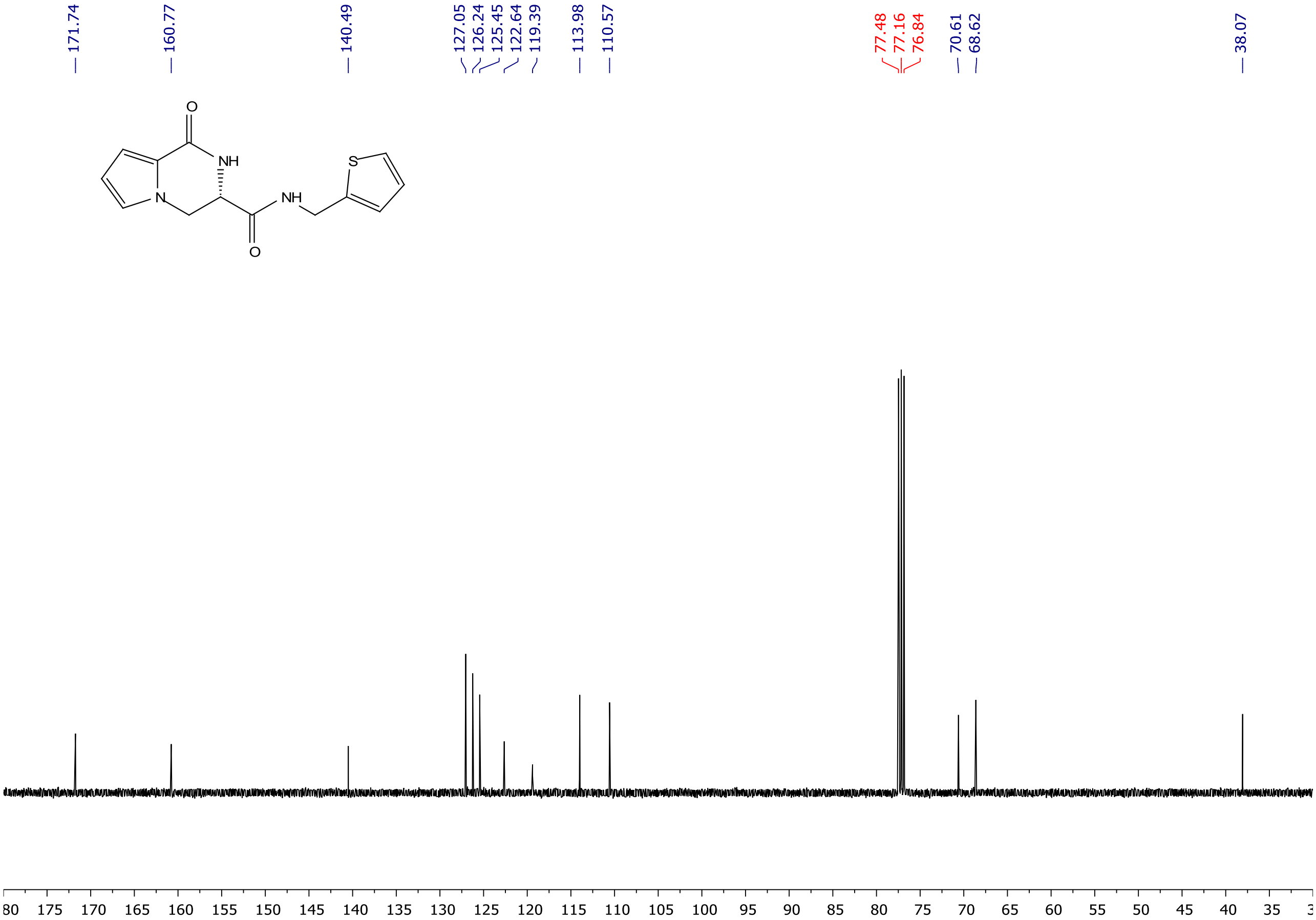

^{1}H and ^{13}C NMR: *(S)-N,2-Dibenzyl-1-oxo-1,2,3,4-tetrahydropyrrolo[1,2-a]pyrazine-3-carboxamide* (***B1***)

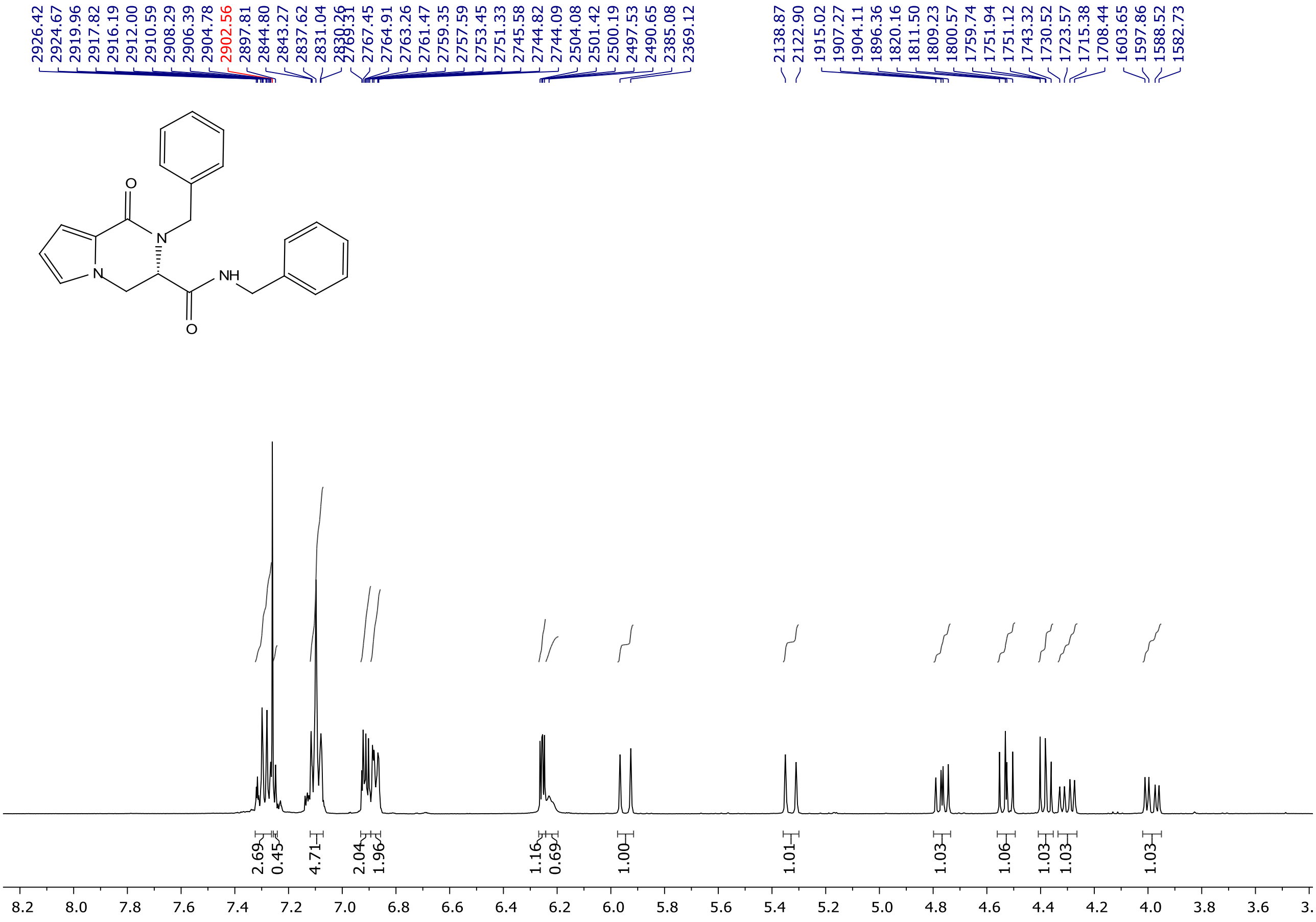

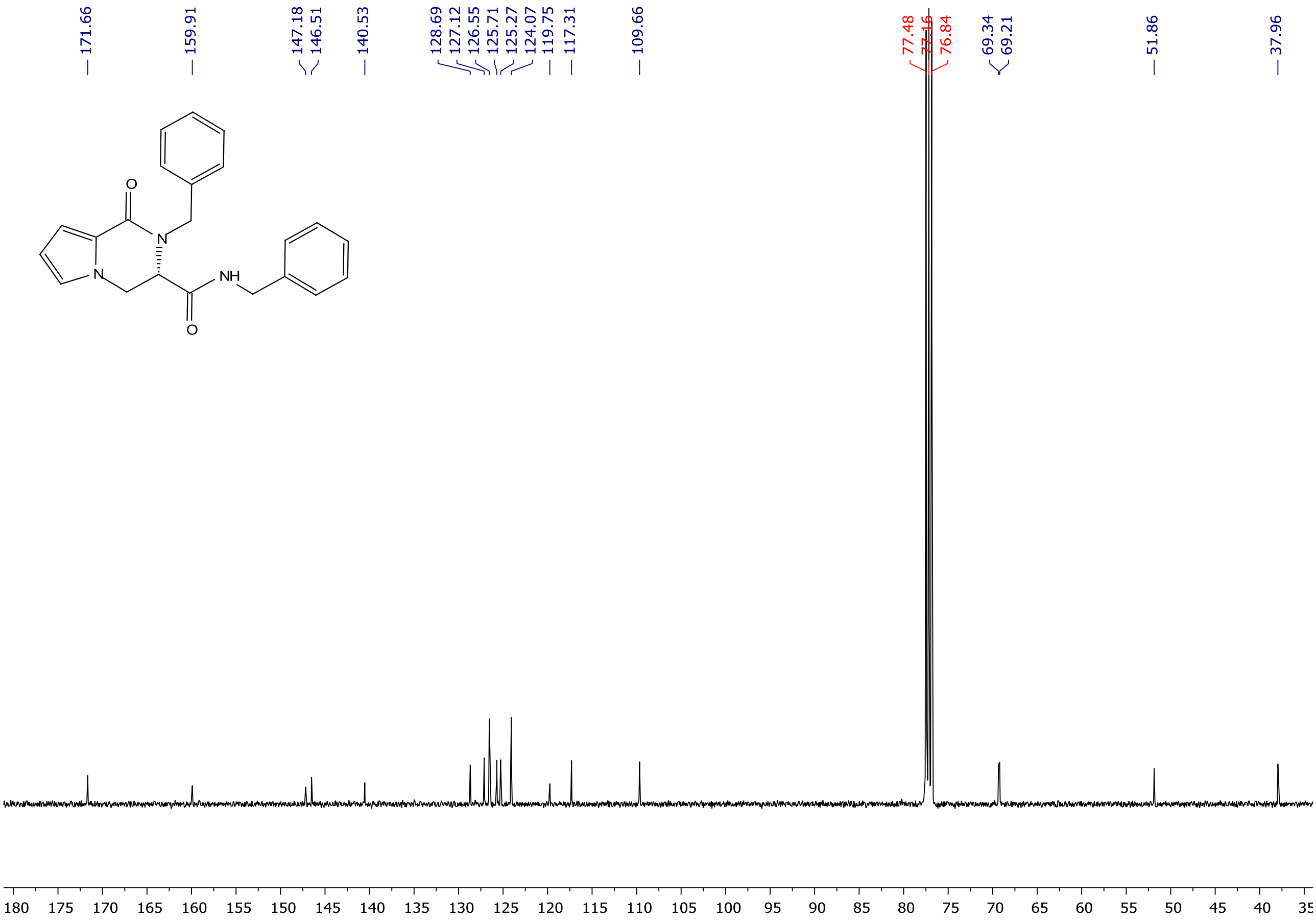

^{1}H and ^{13}C NMR: *(S)-N-(3-Chlorobenzyl)-2-benzyl-1-oxo-1,2,3,4-tetrahydropyrrolo[1,2-a]pyrazine-3-carboxamide (**B2**)*

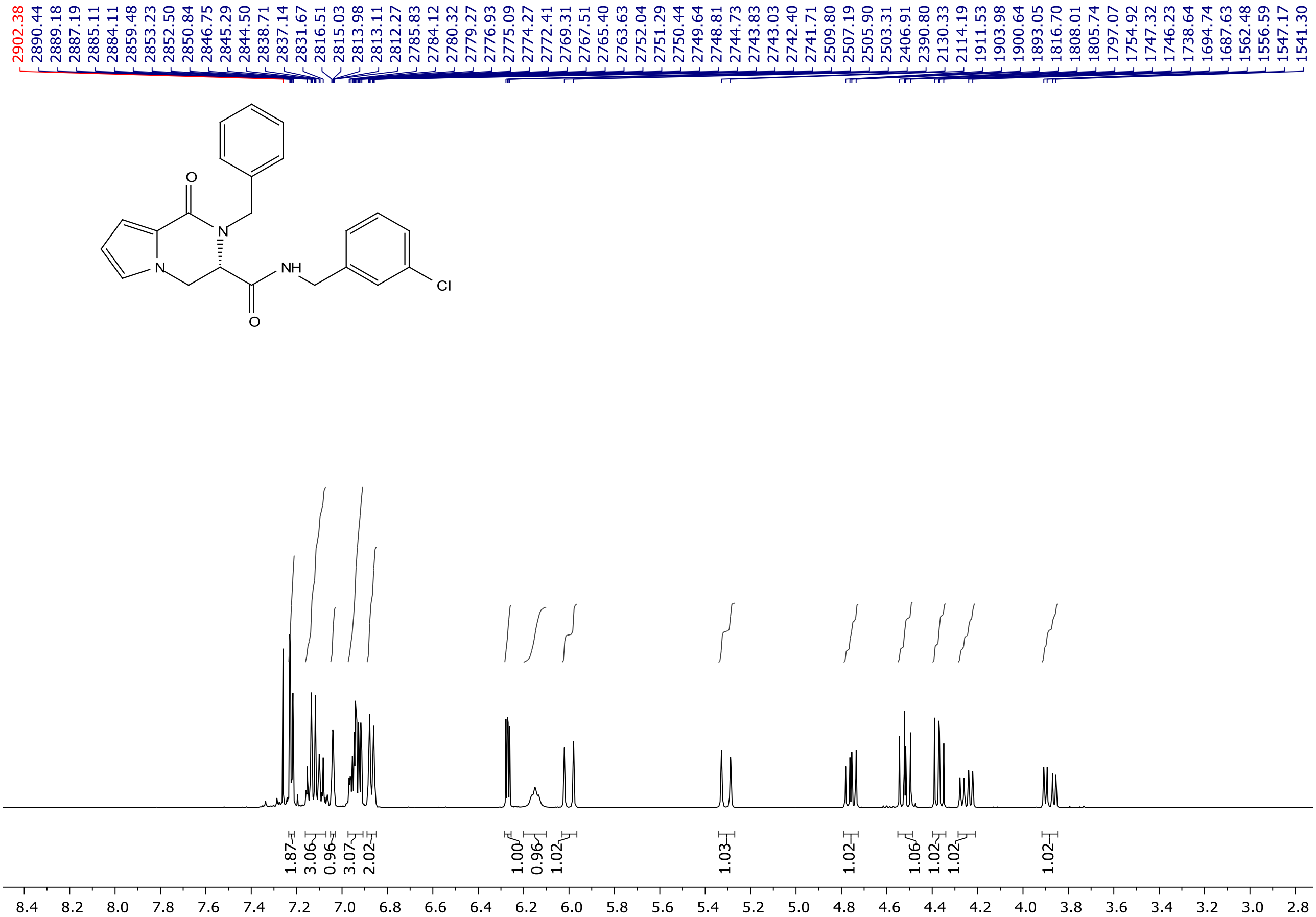

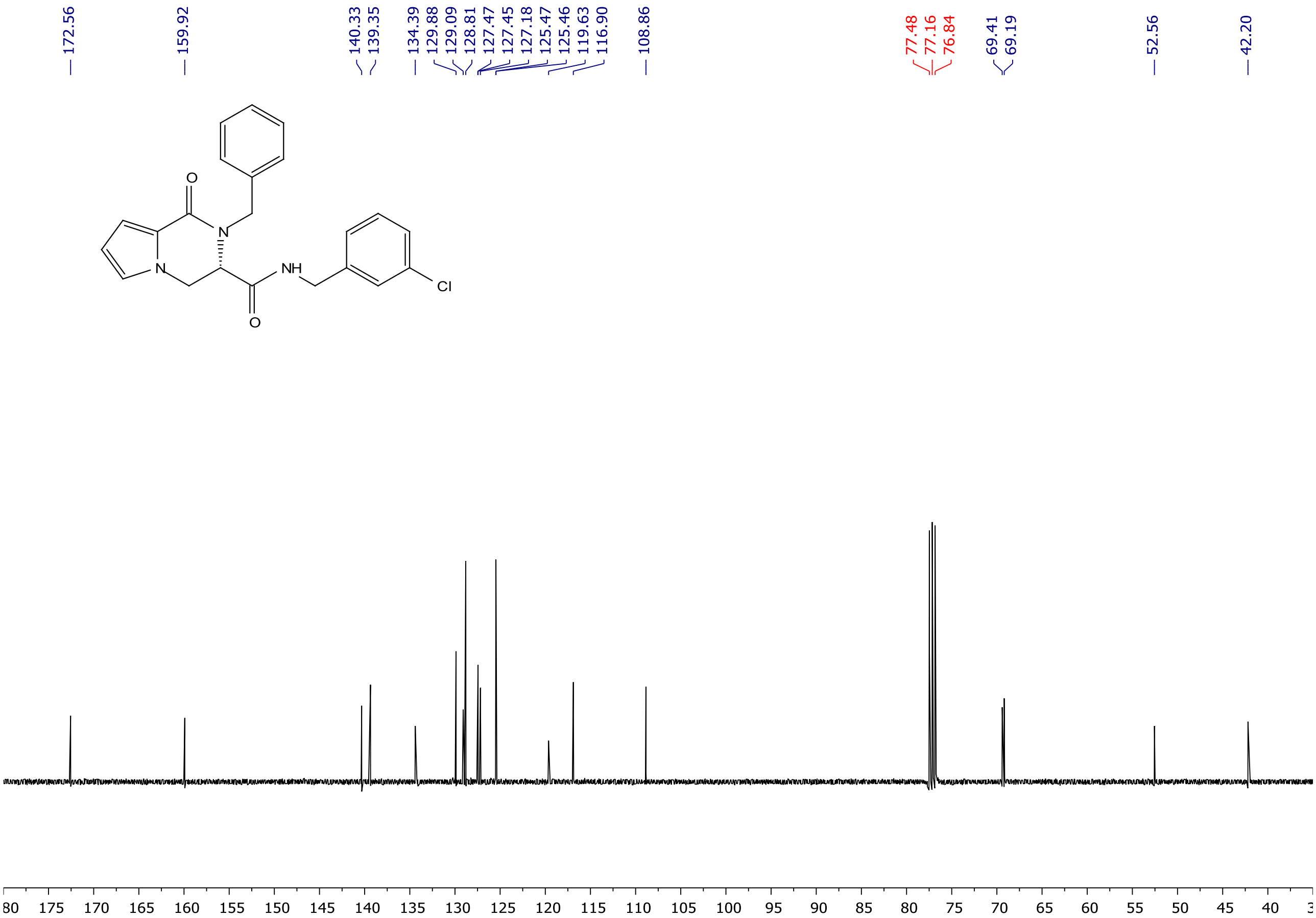

^{1}H and ^{13}C NMR: *(S)-N-(Furan-2-ylmethyl)-2-benzyl-1-oxo-1,2,3,4-tetrahydropyrrolo[1,2-a]pyrazine-3-carboxamide* (***B3***)

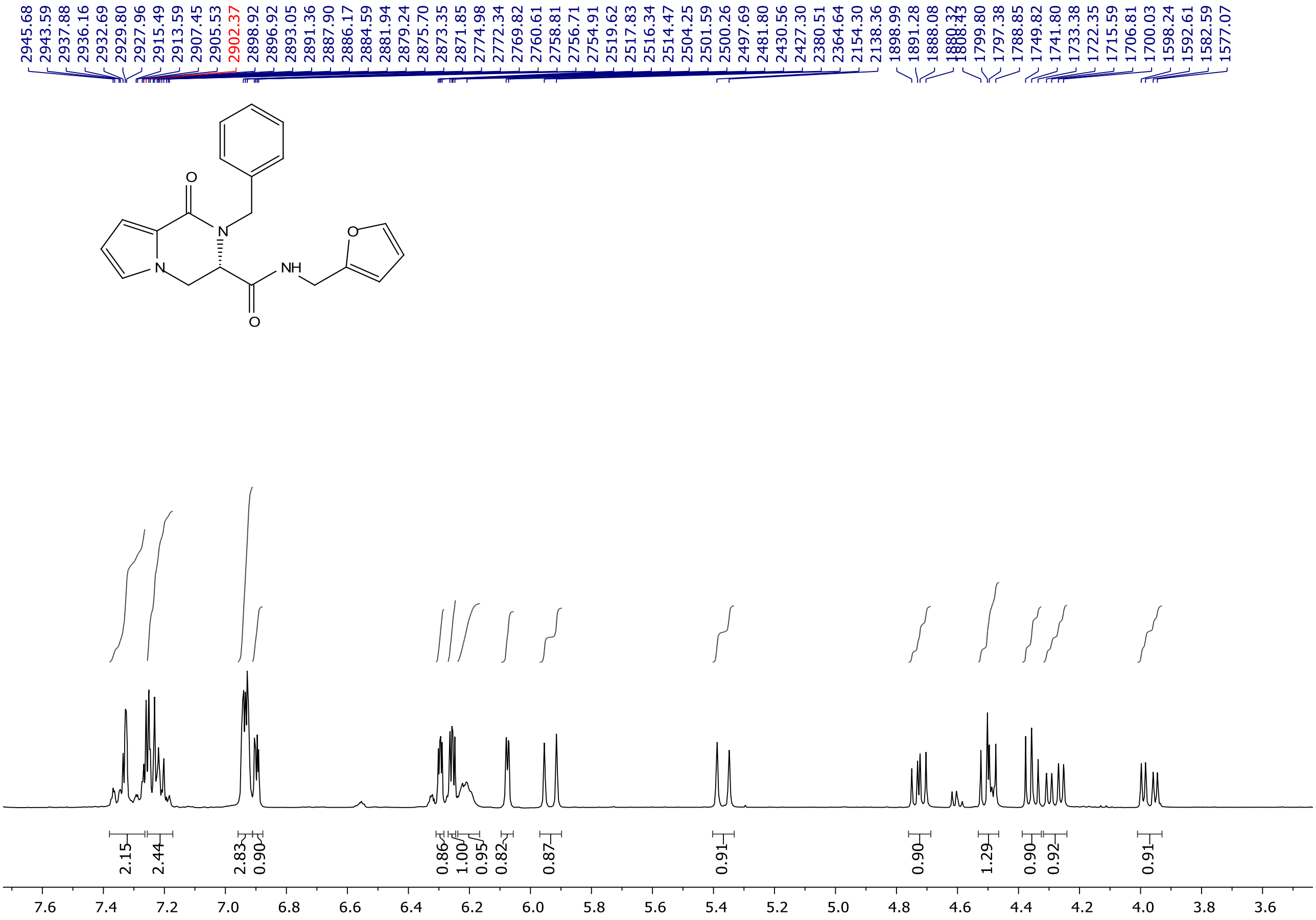

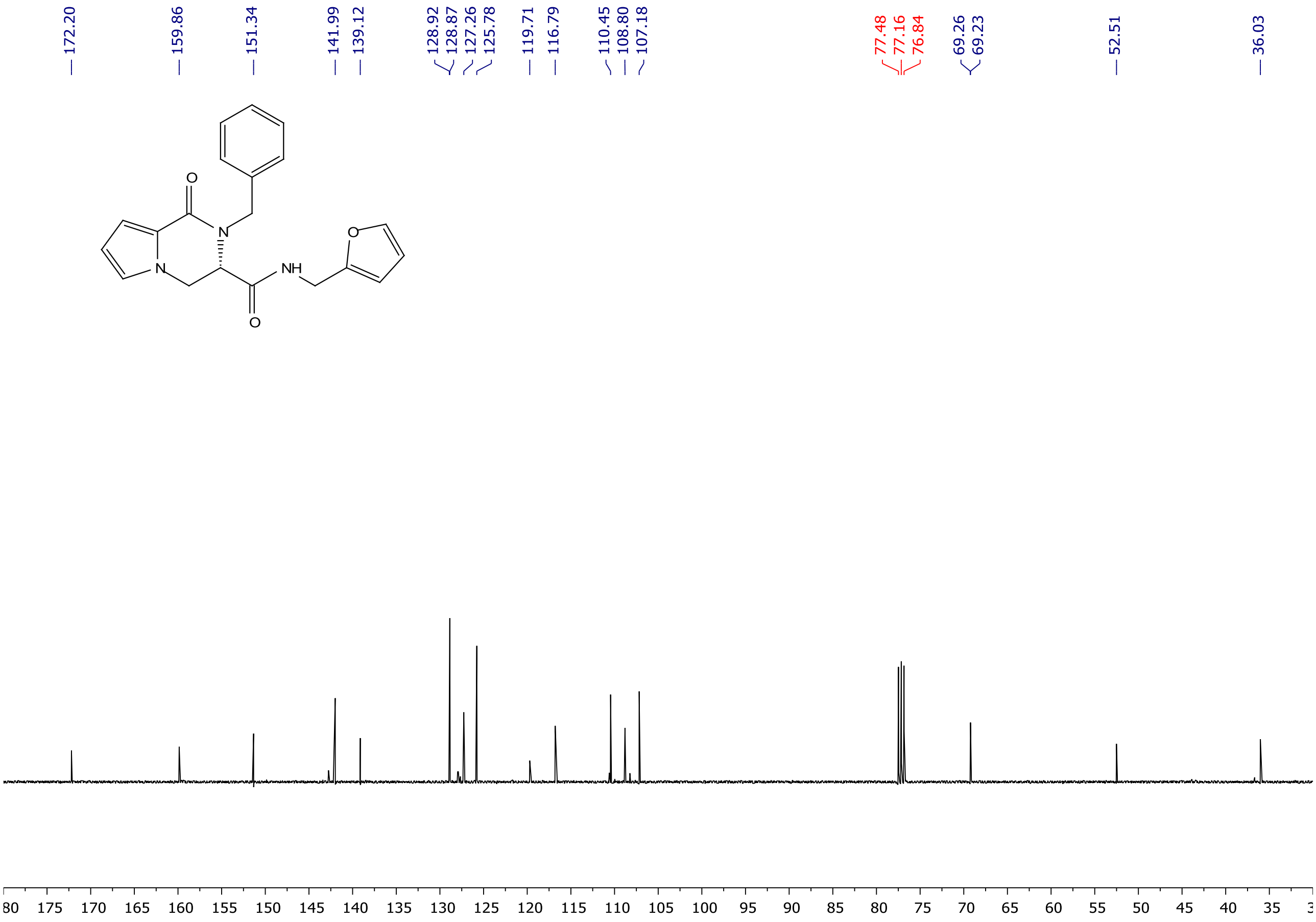

^{1}H and ^{13}C NMR: *(S)-N-((5-Methylfuran-2-yl)methyl)-2-benzyl-1-oxo-1,2,3,4-tetrahydropyrrolo[1,2-a]pyrazine-3-carboxamide (**B4**)*

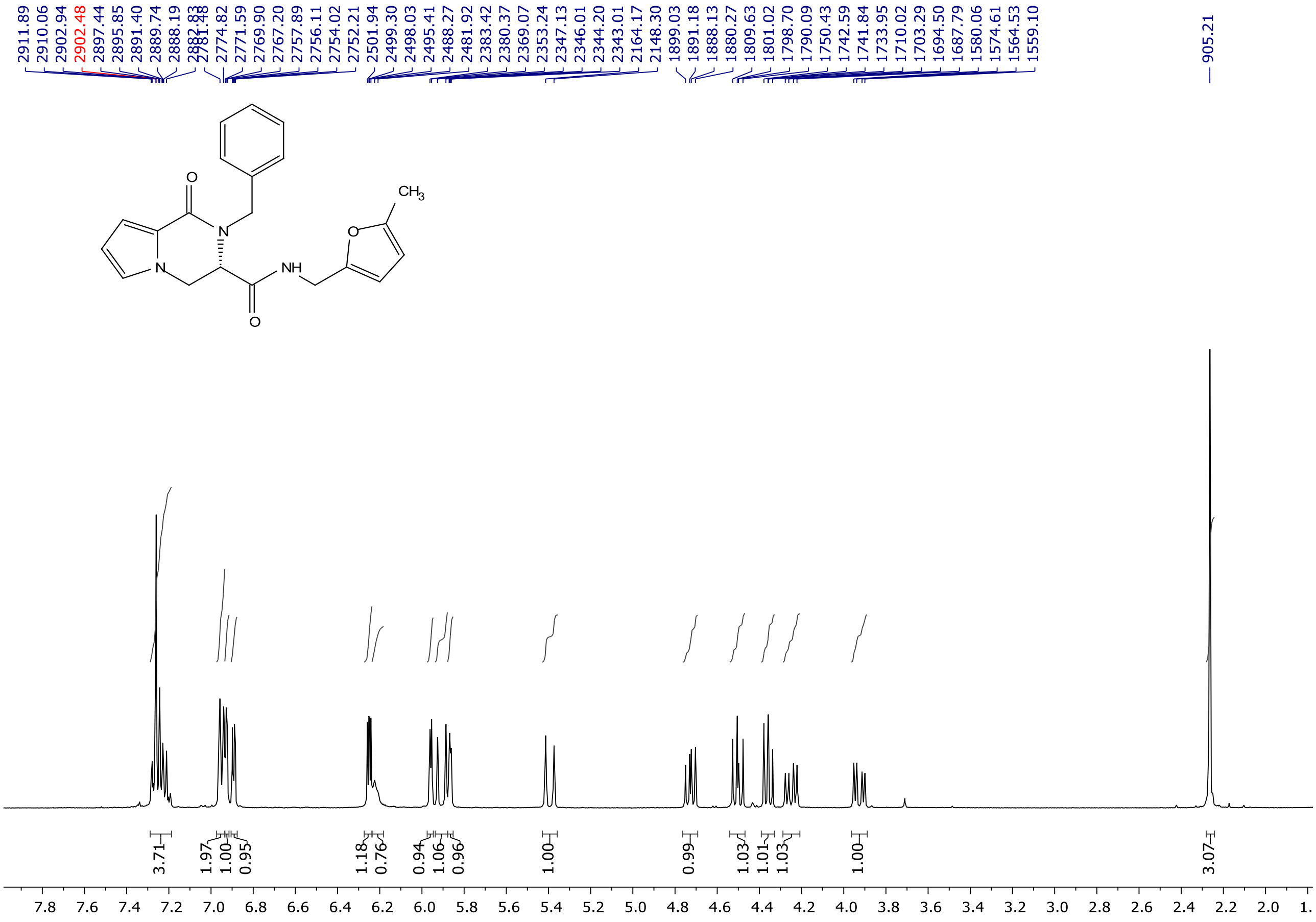

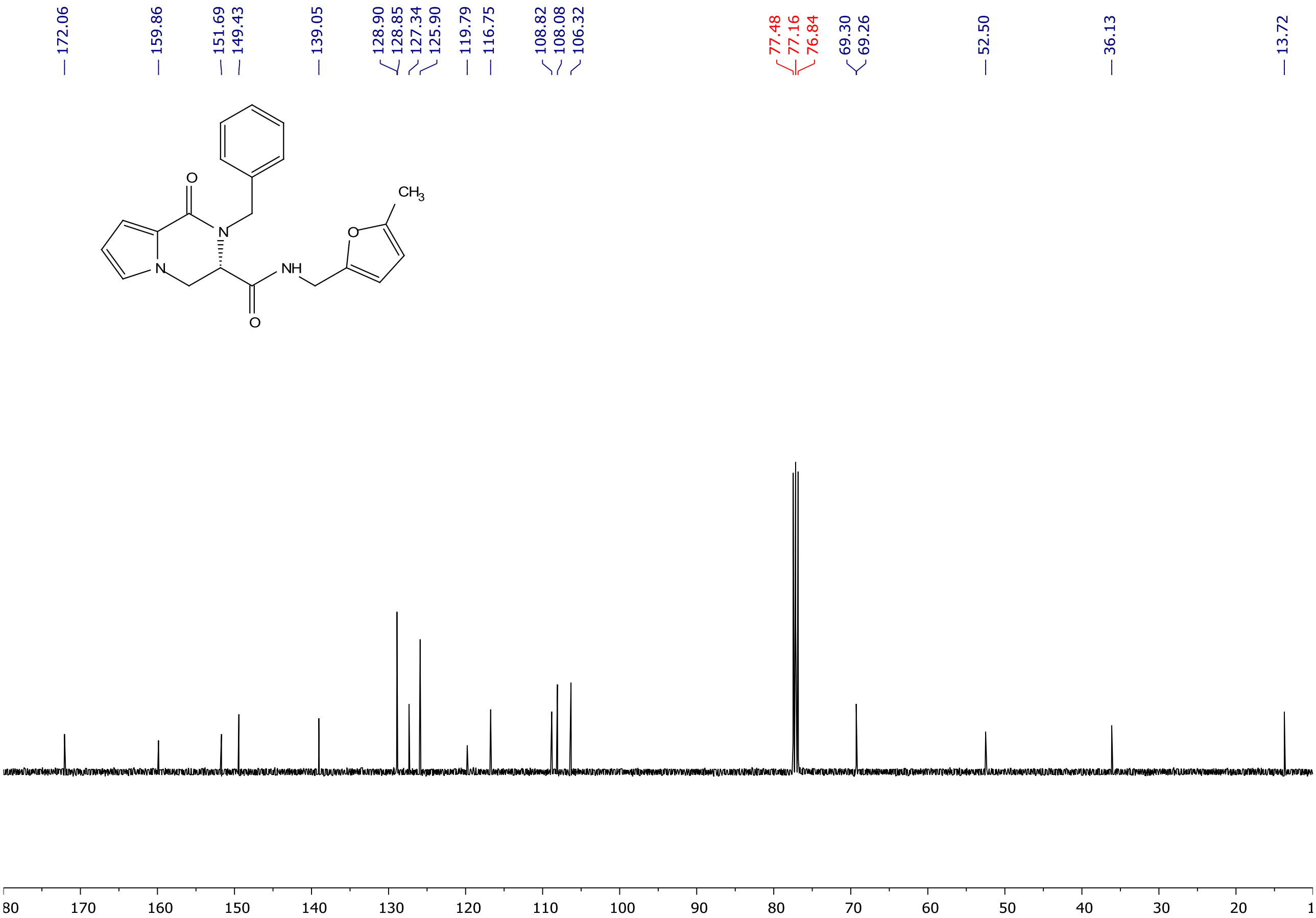

^{1}H and ^{13}C NMR: *(S)-N-(Thiophen-2-ylmethyl)-2-benzyl-1-oxo-1,2,3,4-tetrahydropyrrolo[1,2-a]pyrazine-3-carboxamide* (***B5***)

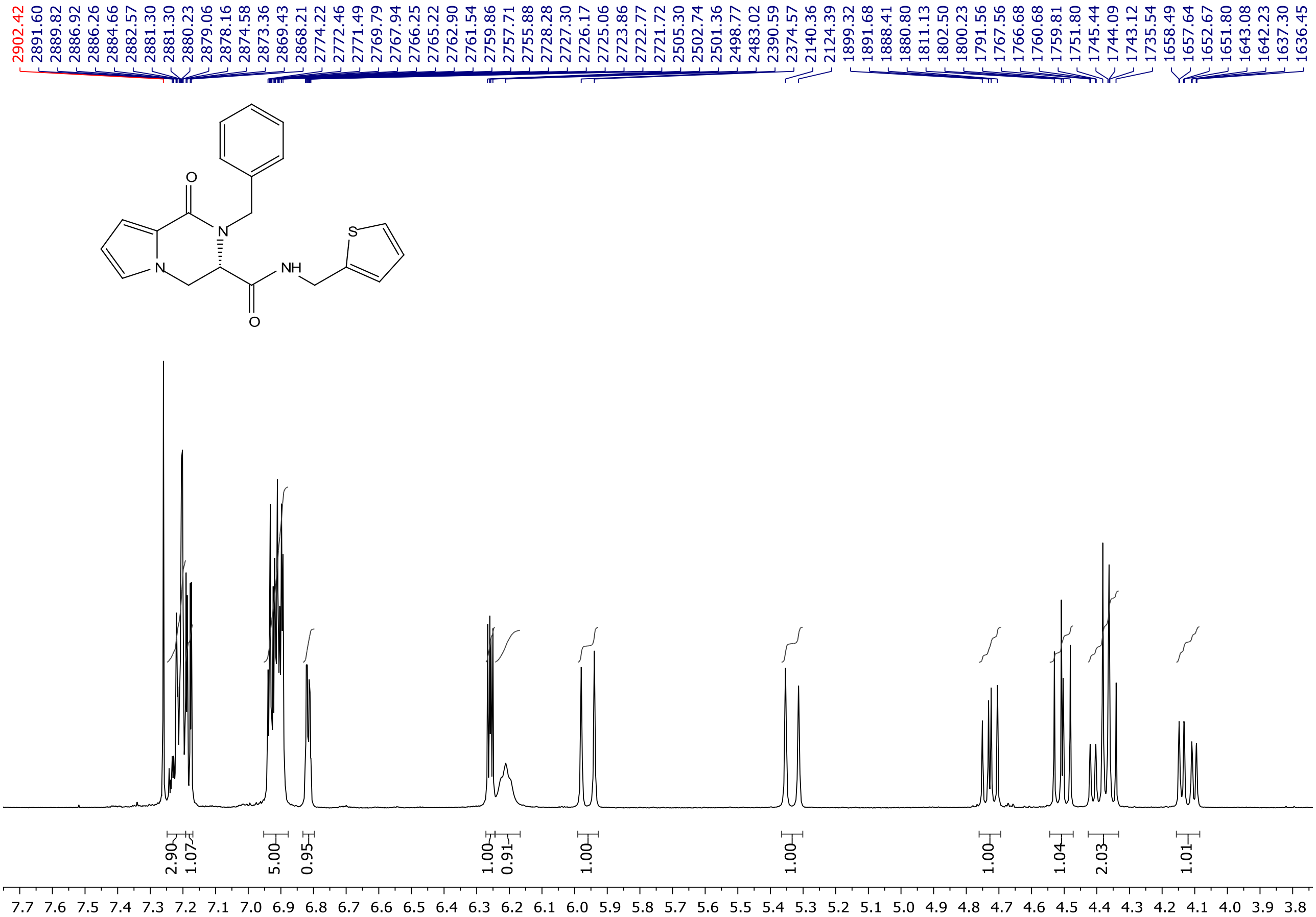

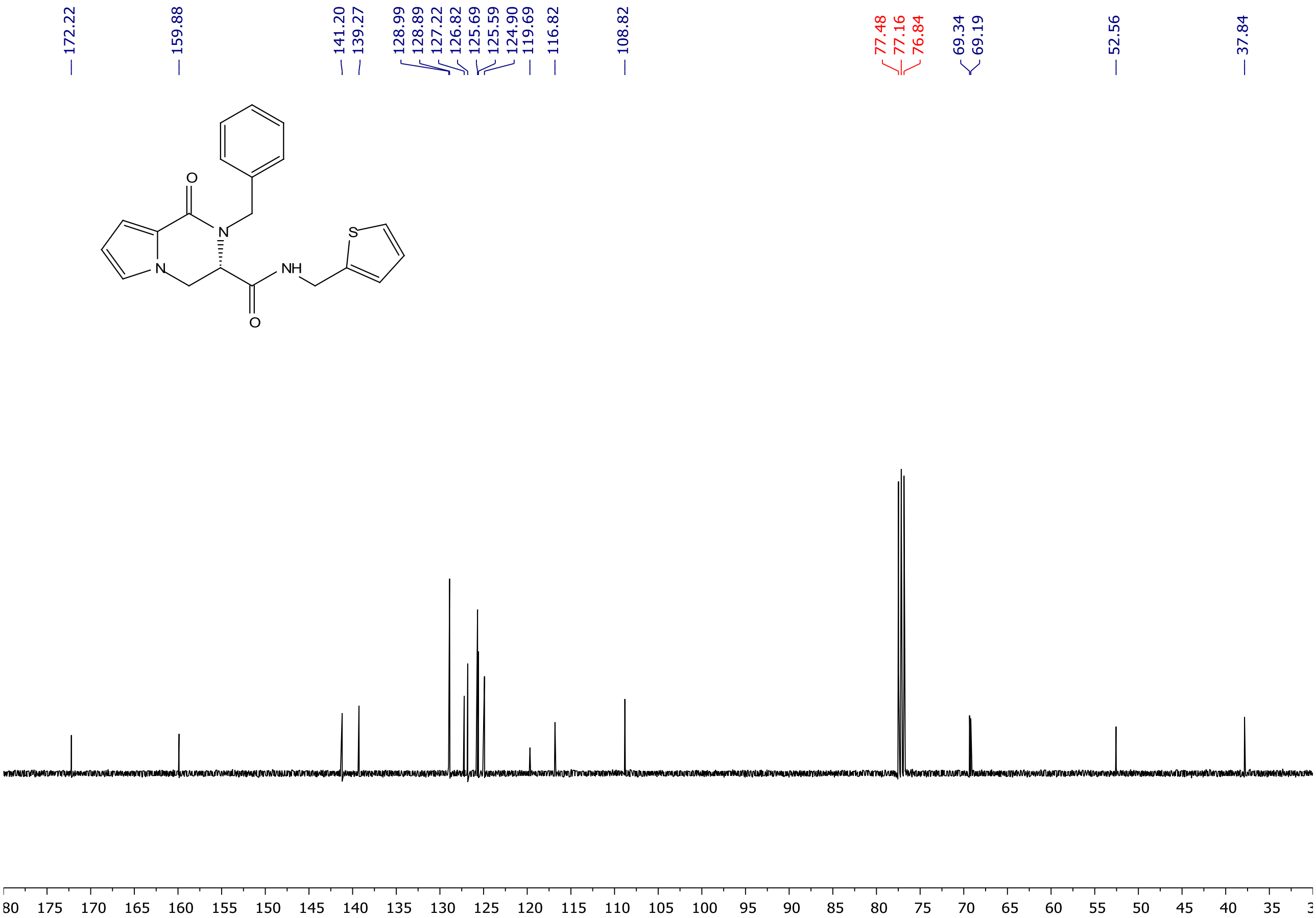

^{1}H and ^{13}C NMR: *(S)-N-Benzyl-2-(4-cyanobenzyl)-1-oxo-1,2,3,4-tetrahydropyrrolo[1,2-a]pyrazine-3-carboxamide* (***C1***)

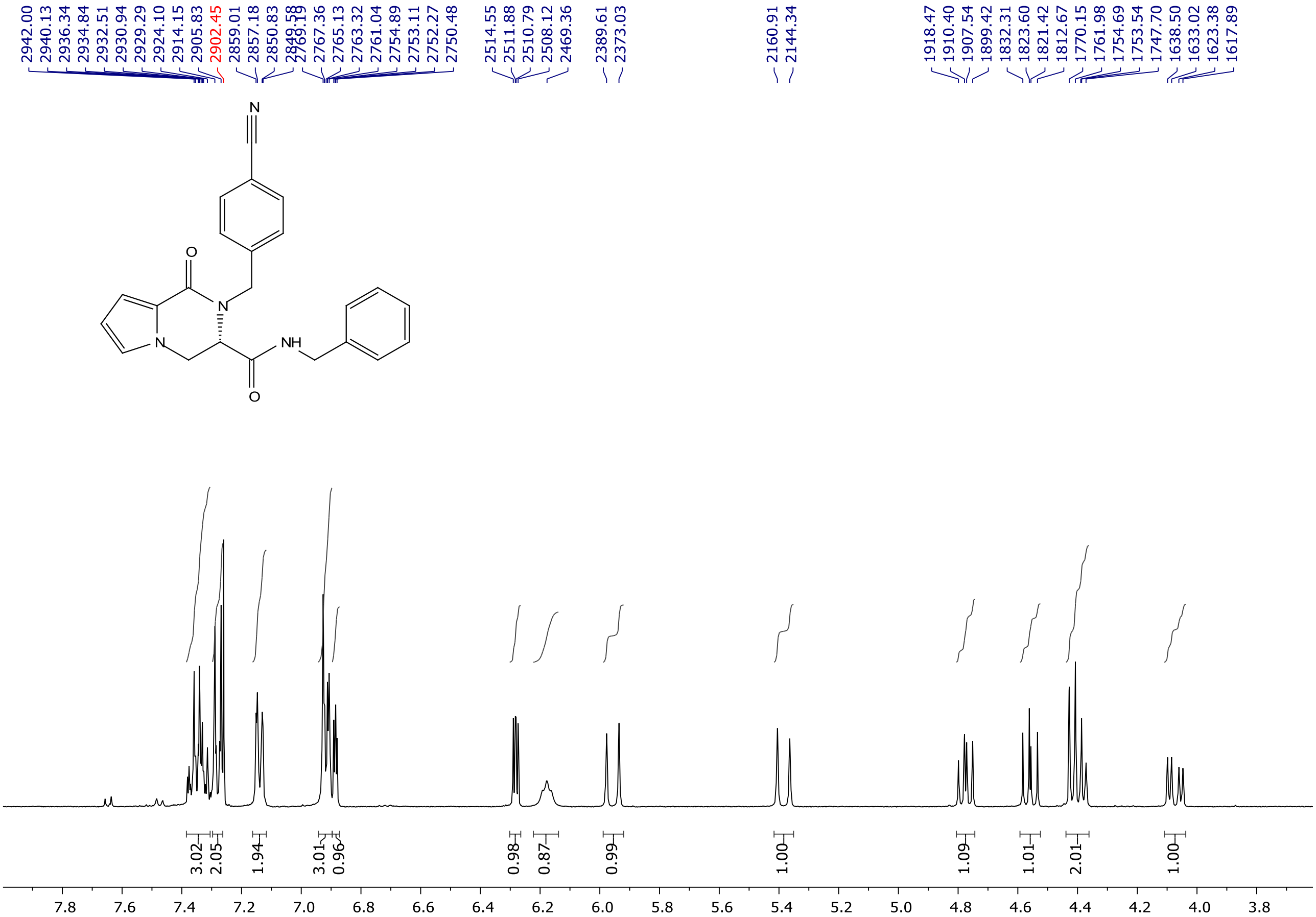

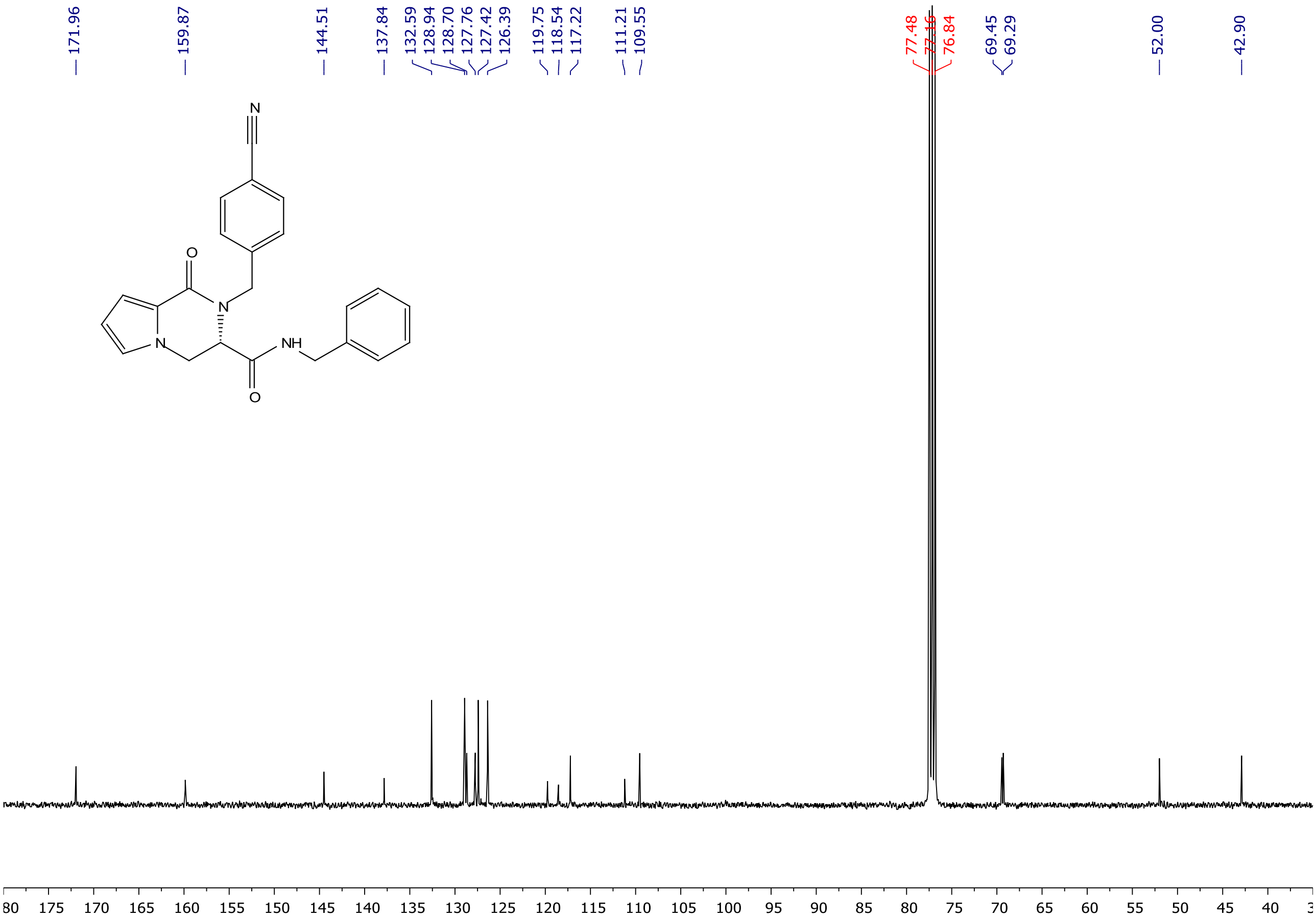

^{1}H and ^{13}C NMR: *(S)-N-(3-Chlorobenzyl)-2-(4-cyanobenzyl)-1-oxo-1,2,3,4-tetrahydropyrrolo[1,2-a]pyrazine-3-carboxamide* (***C2***)

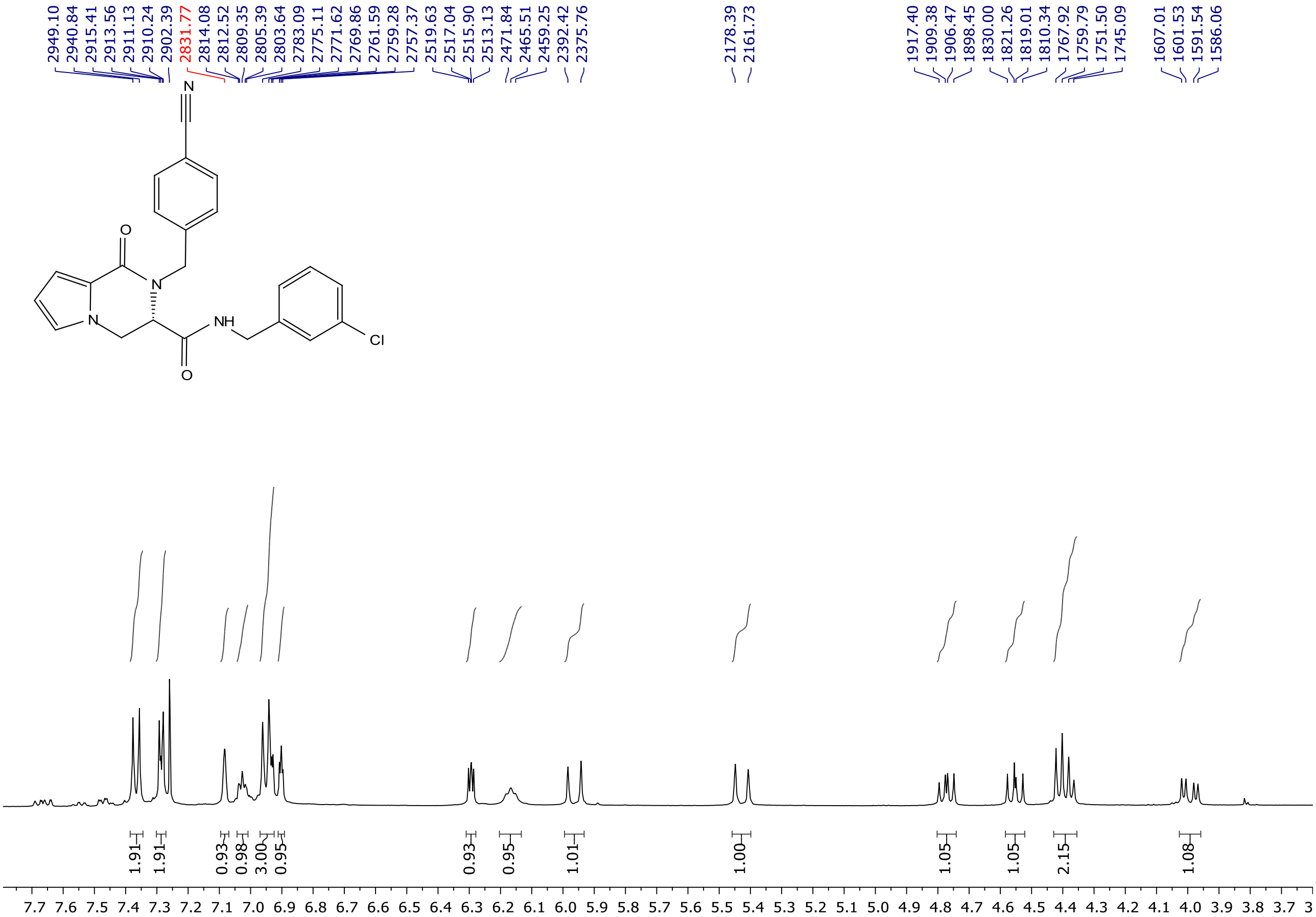

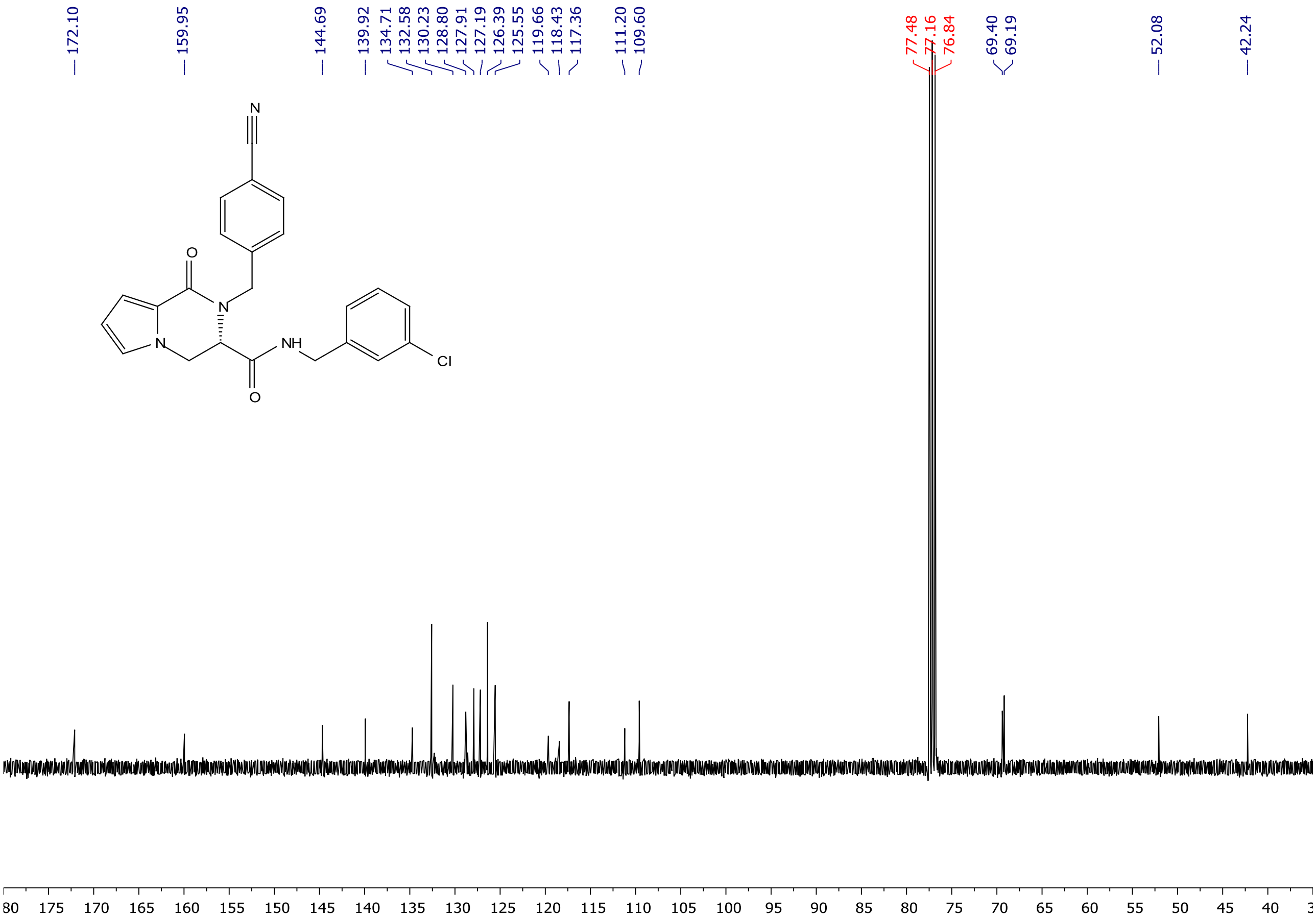

^1H and ^{13}C NMR: *(S)-N-(Furan-2-ylmethyl)-2-(4-cyanobenzyl)-1-oxo-1,2,3,4-tetrahydropyrrolo[1,2-a]pyrazine-3-carboxamide* (***C3***)

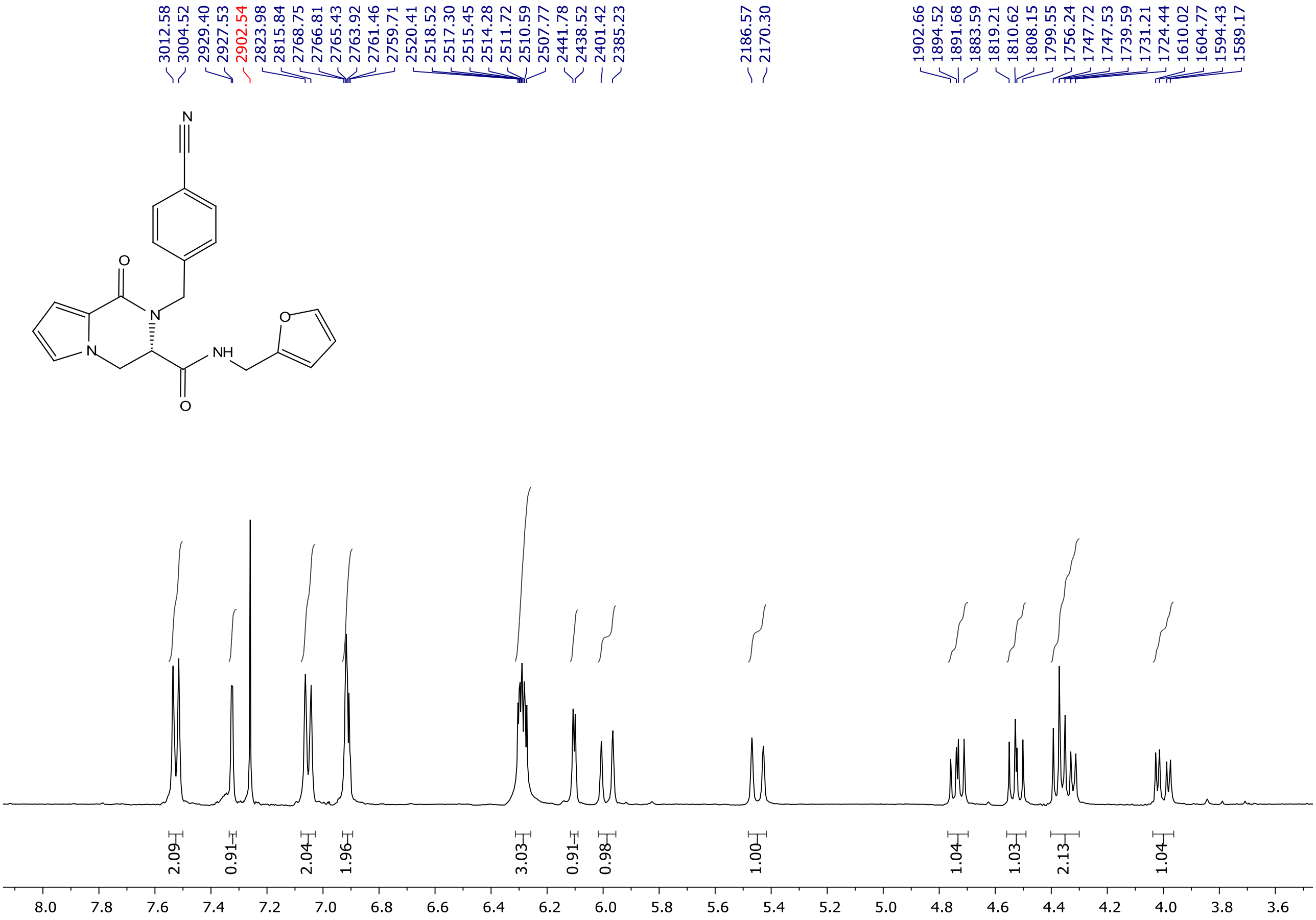

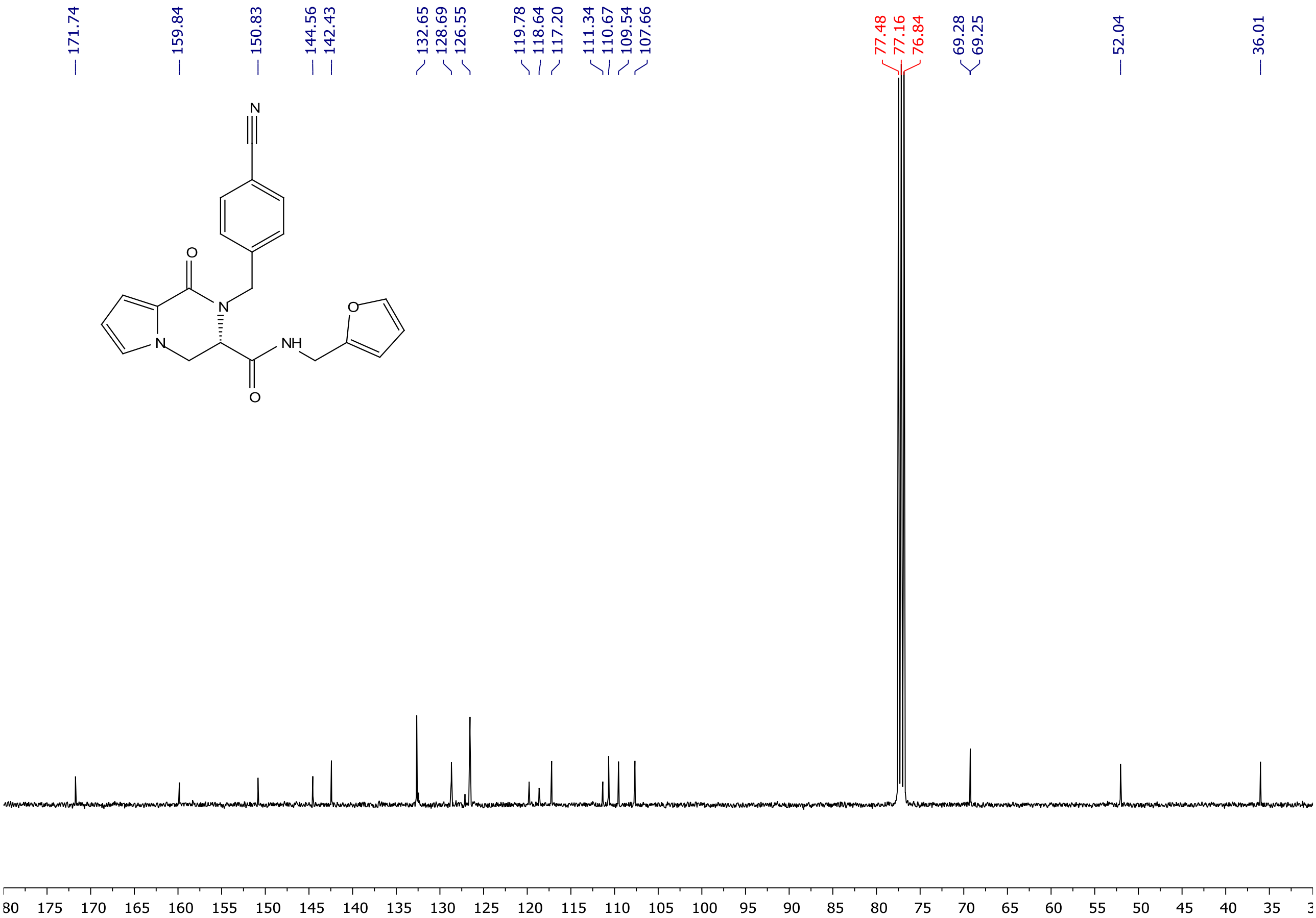

^{1}H and ^{13}C NMR: *(S)-N-((5-Methylfuran-2-yl)methyl)-2-(4-cyanobenzyl)-1-oxo-1,2,3,4-tetrahydropyrrolo[1,2-a]pyrazine-3-carboxamide* (***C4***)

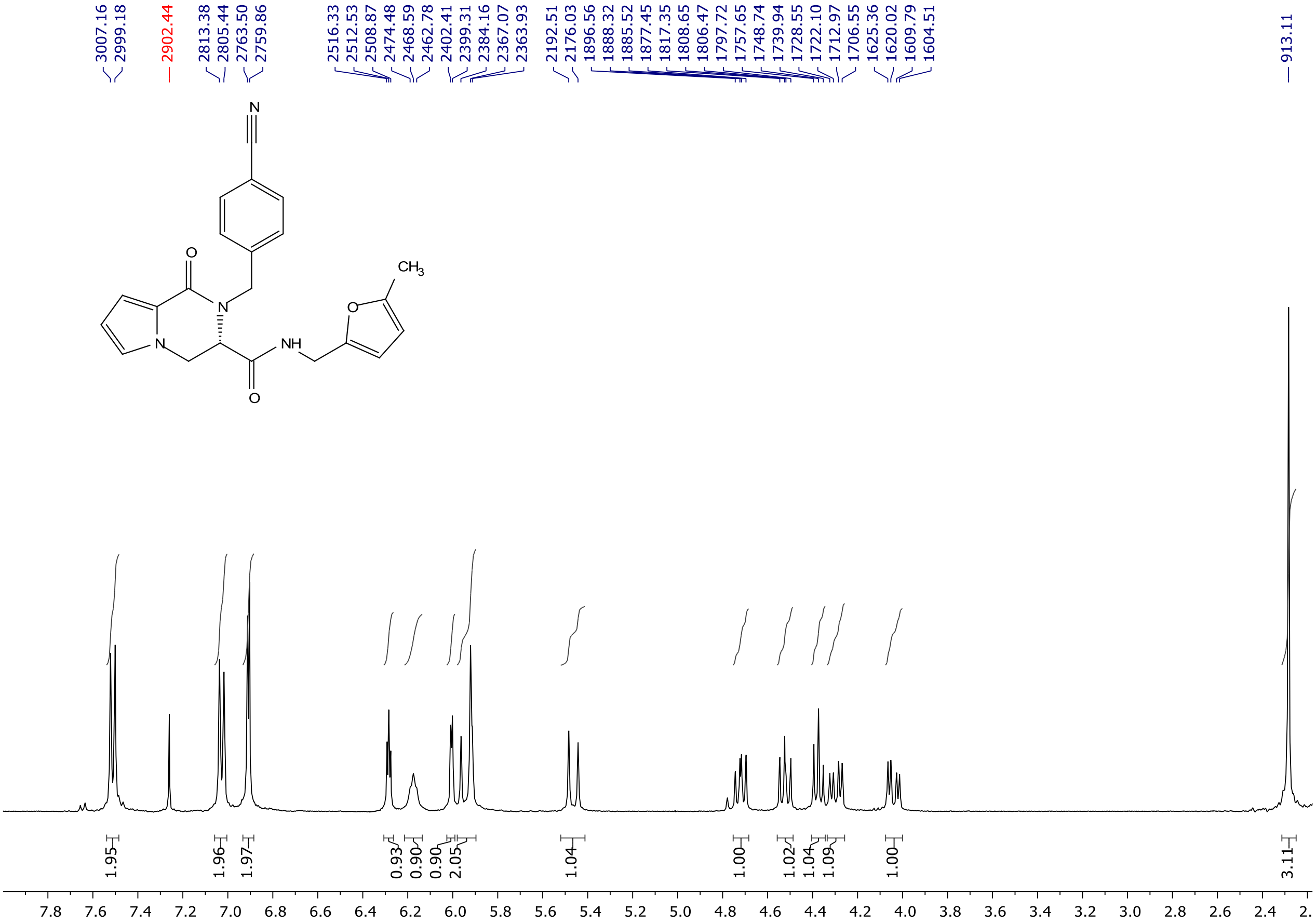

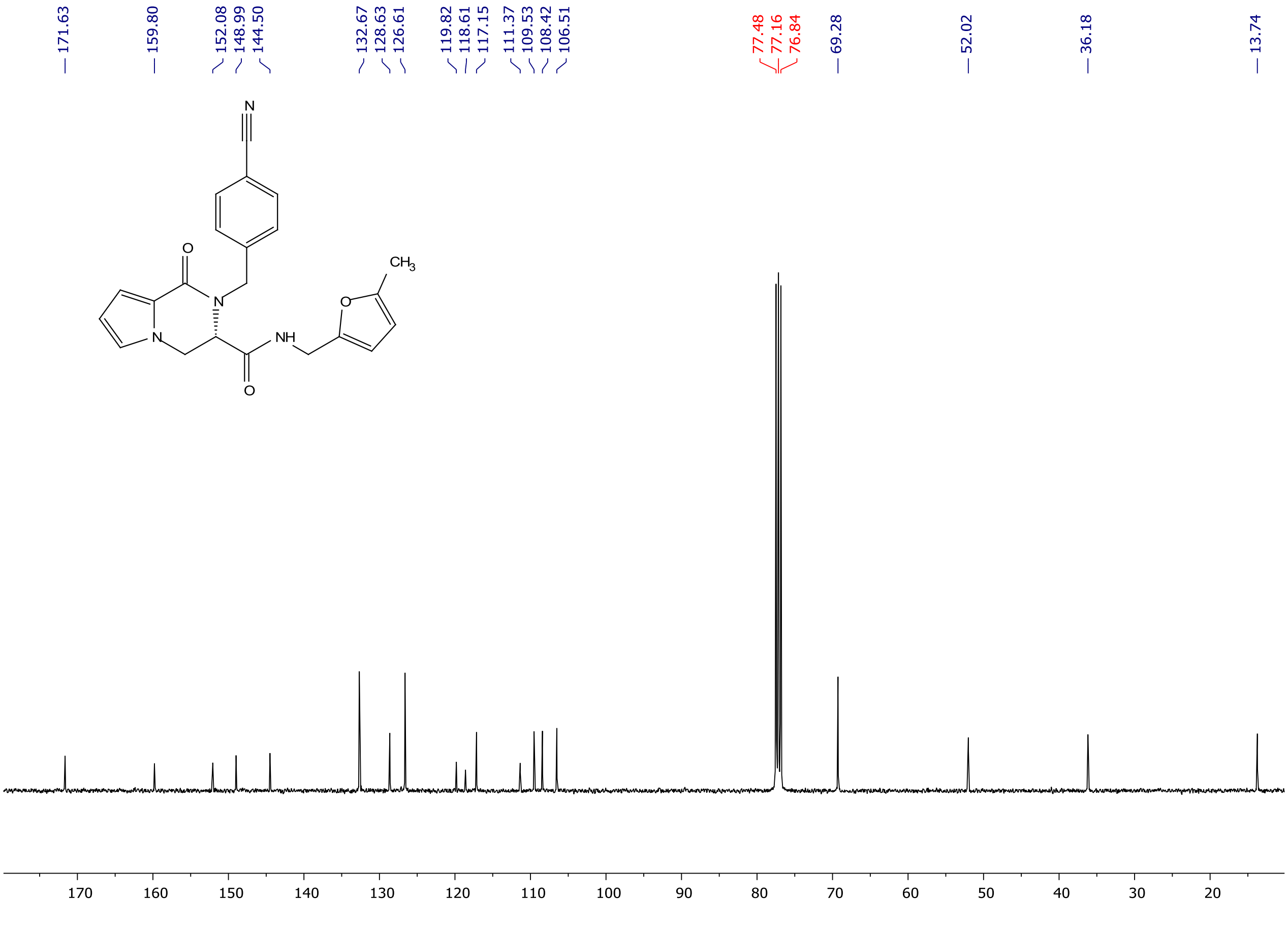

^{1}H and ^{13}C NMR: *(S)-N-(Thiophen-2-ylmethyl)-2-(4-cyanobenzyl)-1-oxo-1,2,3,4-tetrahydropyrrolo[1,2-a]pyrazine-3-carboxamide* ***(C5)***

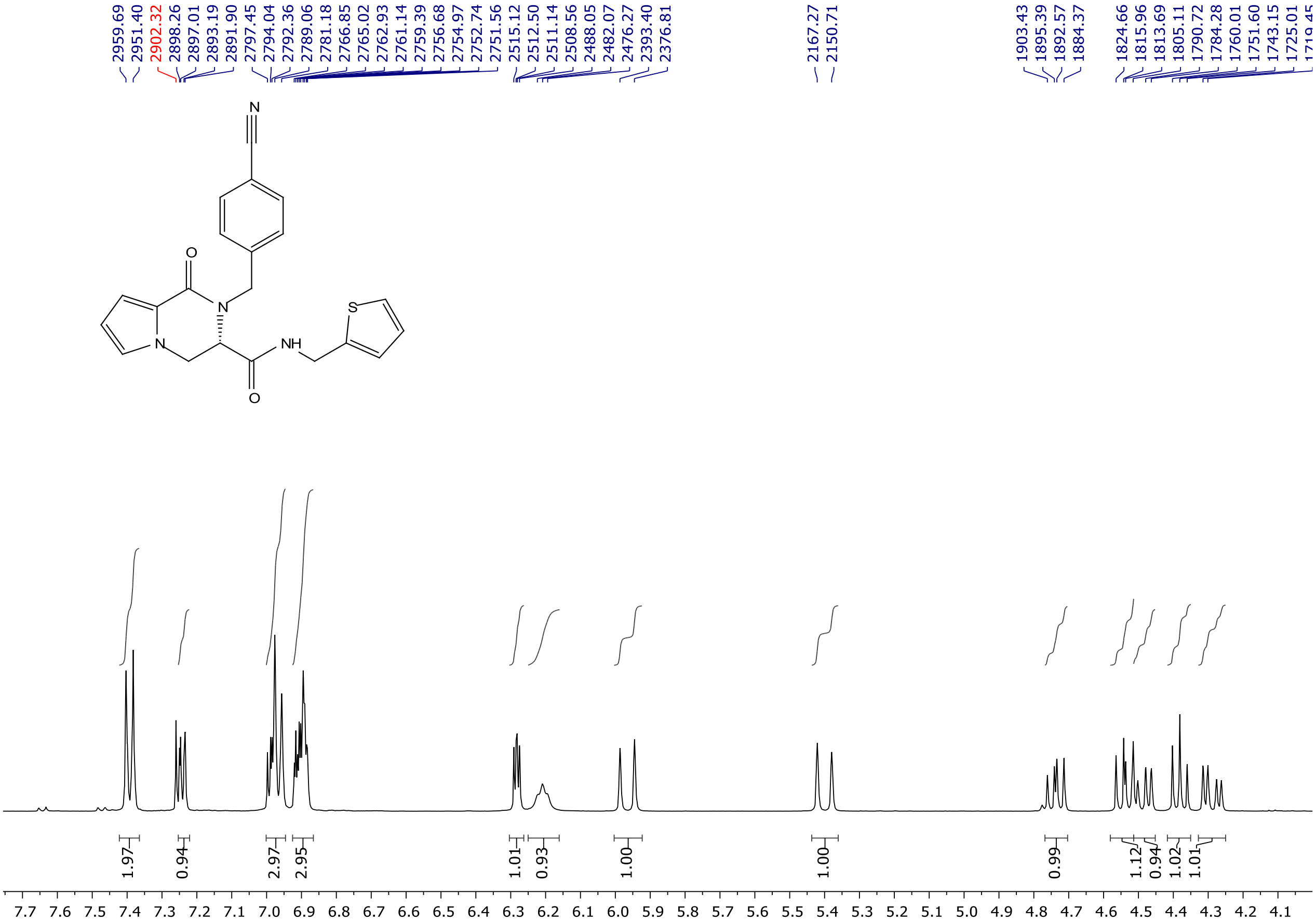

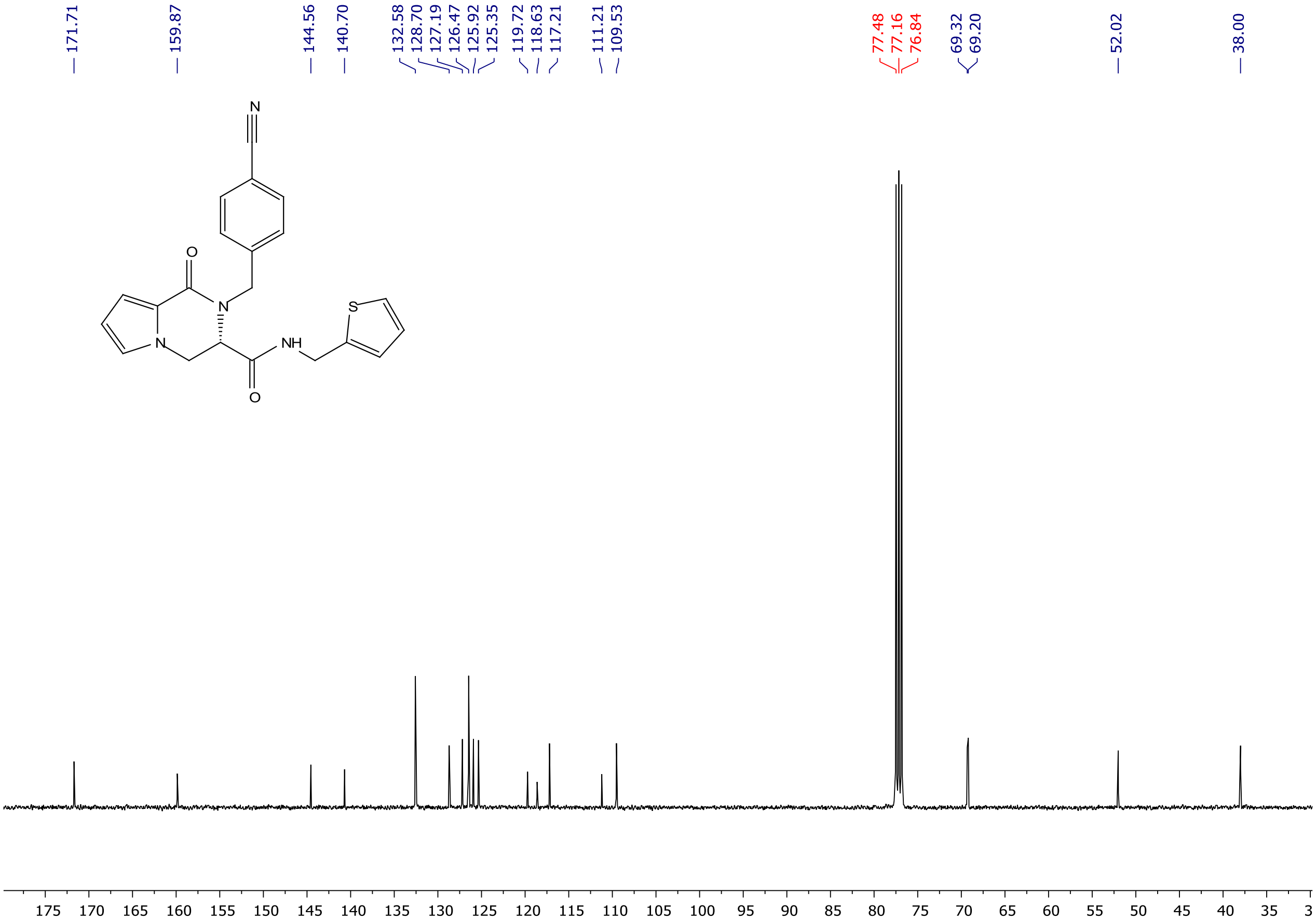

^{1}H and ^{13}C NMR: *(S)-N-Benzyl-1-oxo-2-(4-(trifluoromethyl)benzyl)-1,2,3,4-tetrahydropyrrolo[1,2-a]pyrazine-3-carboxamide (**D1**)*

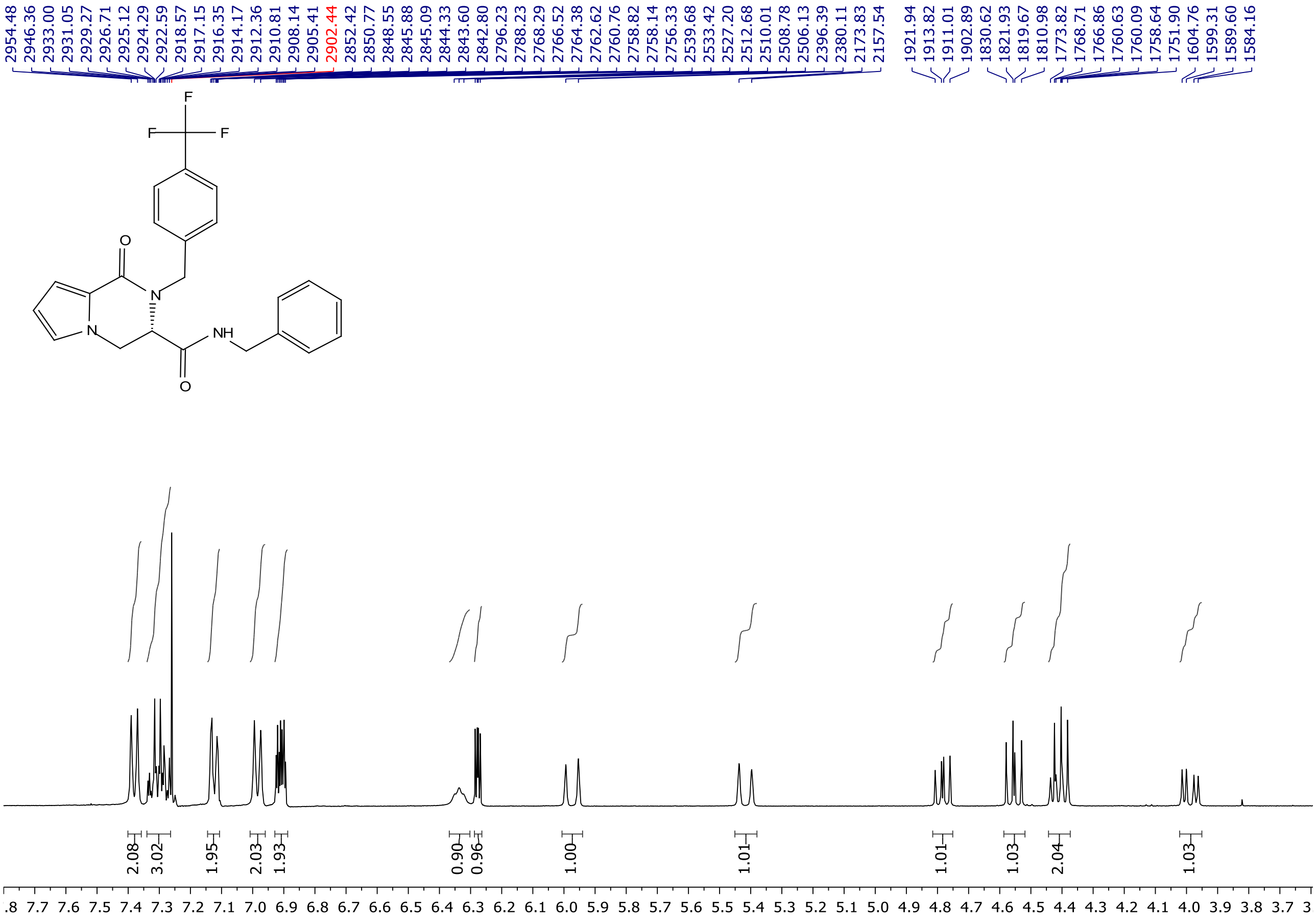

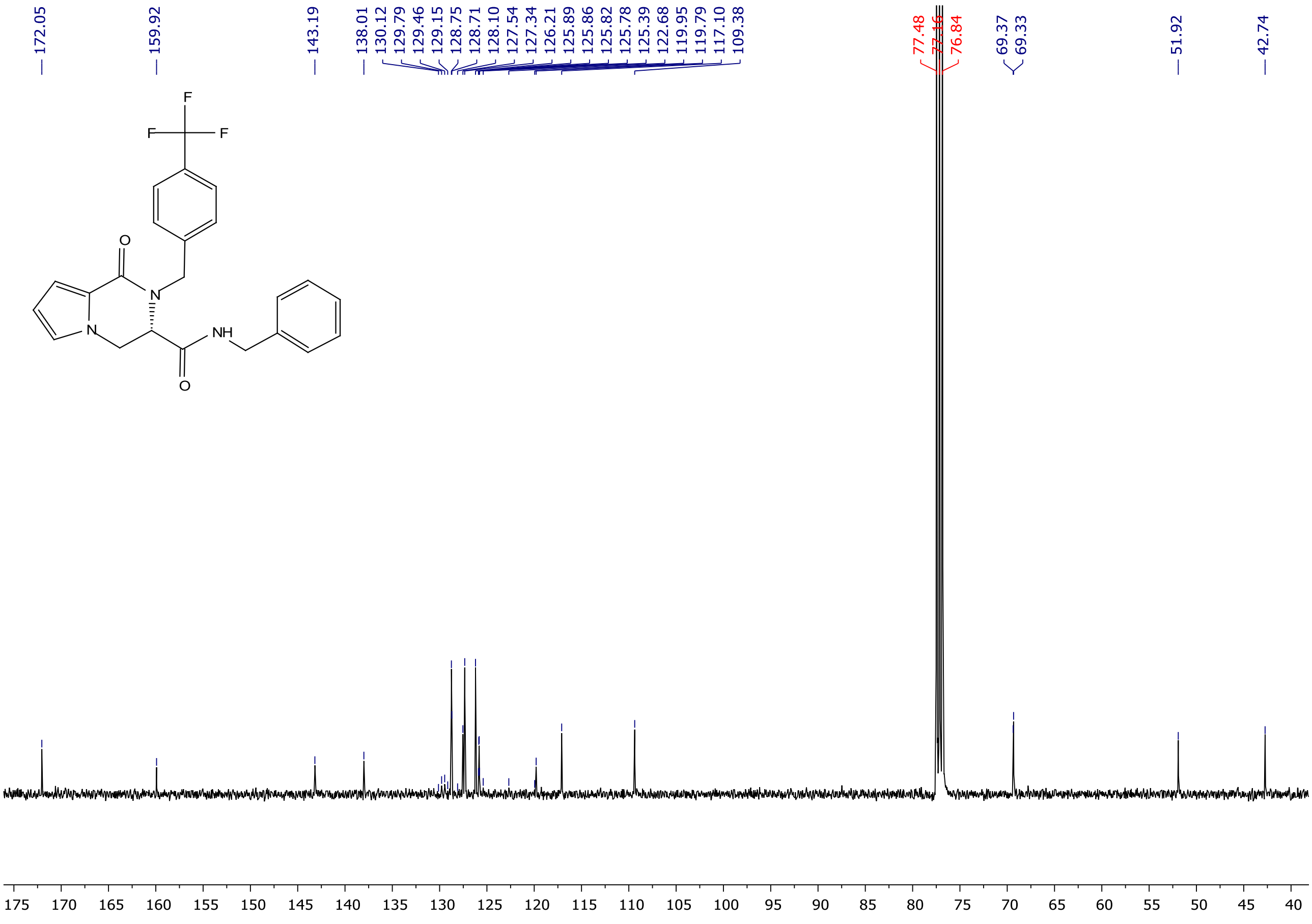

^{1}H and ^{13}C NMR: *(S)-N-(3-Chlorobenzyl)-1-oxo-2-(4-(trifluoromethyl)benzyl)-1,2,3,4-tetrahydropyrrolo[1,2-a]pyrazine-3-carboxamide* (***D2***)

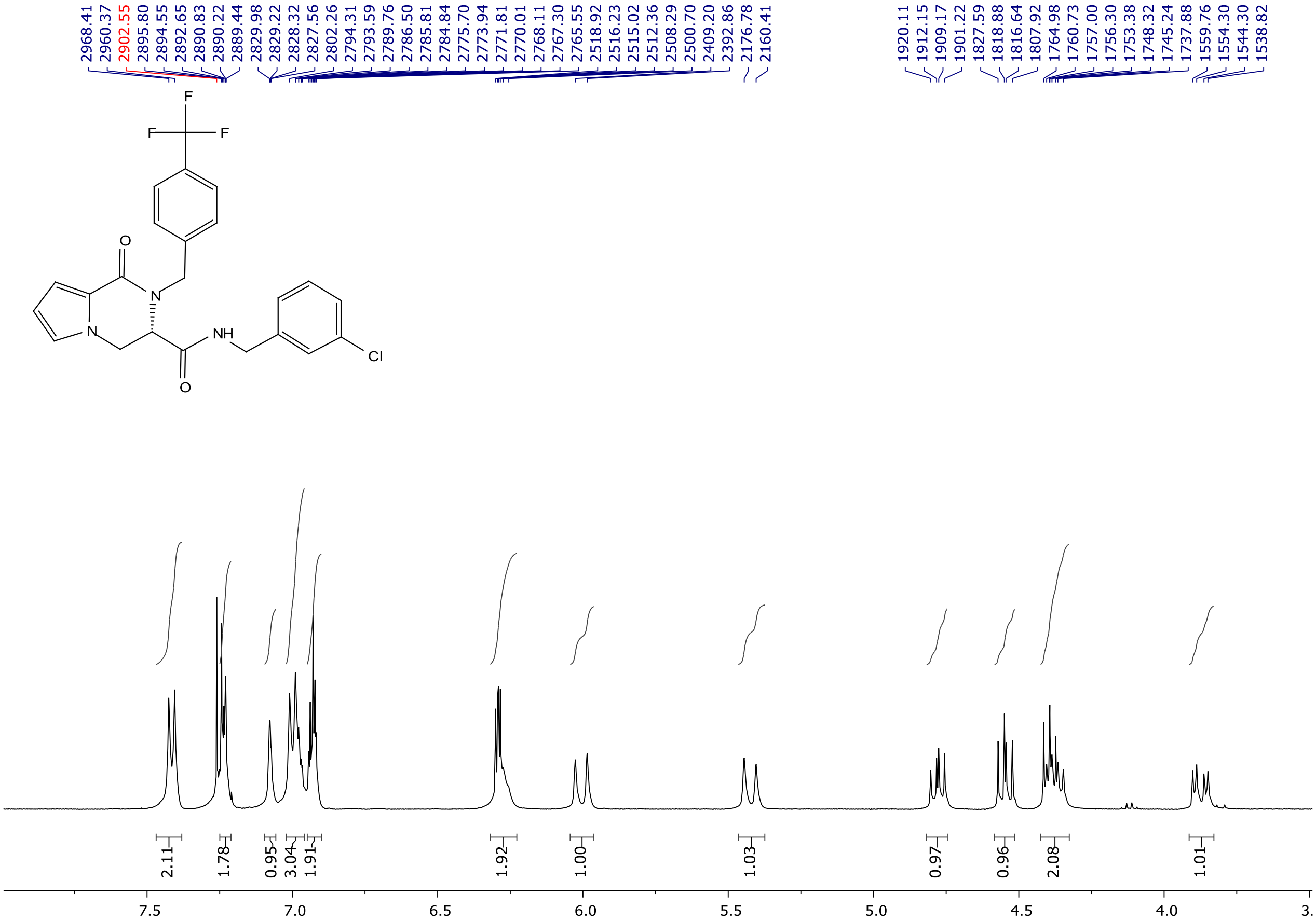

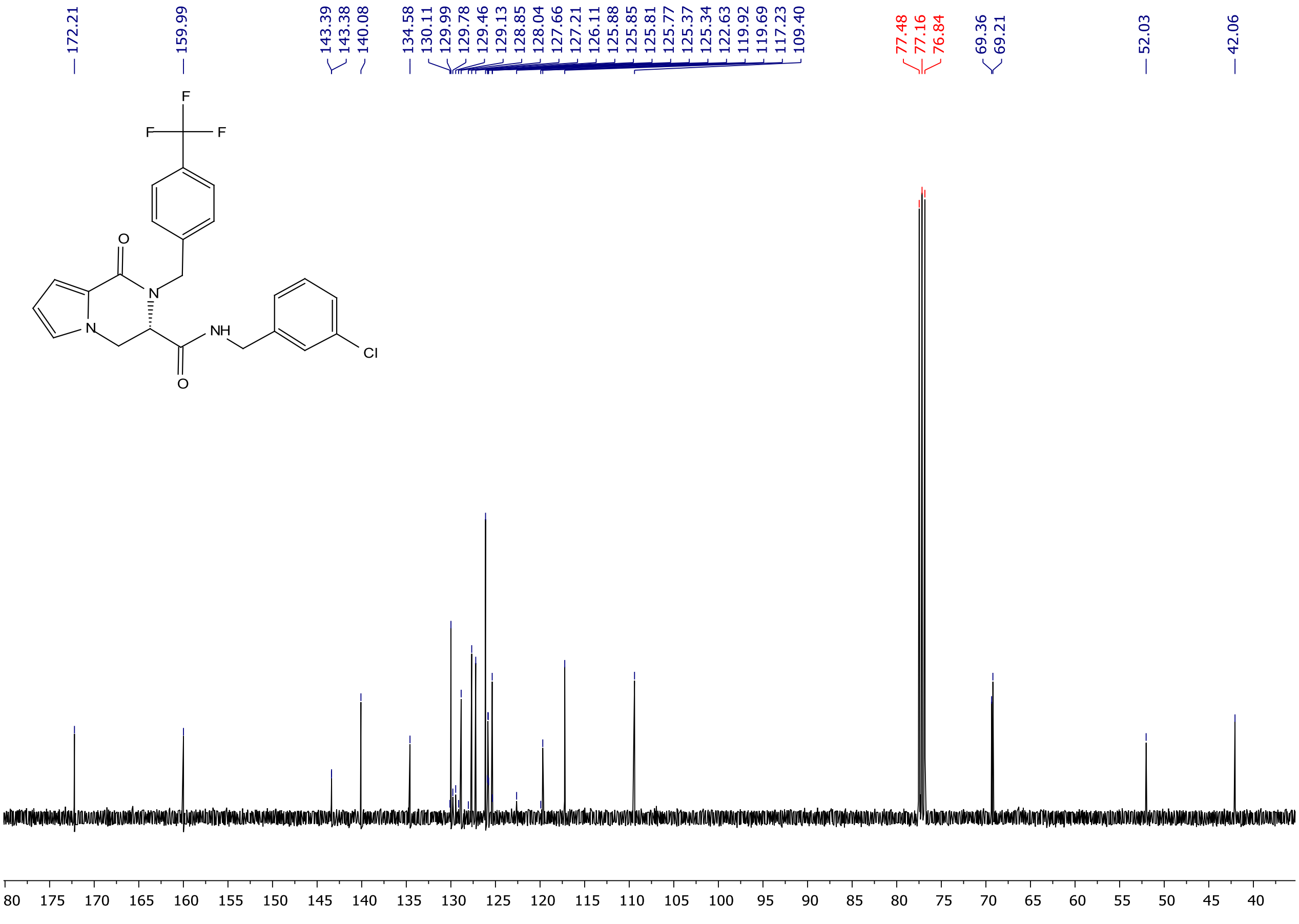

^{1}H and ^{13}C NMR: *(S)-N-(Furan-2-ylmethyl)-1-oxo-2-(4-(trifluoromethyl)benzyl)-1,2,3,4-tetrahydropyrrolo[1,2-a]pyrazine-3-carboxamide* (***D3***)

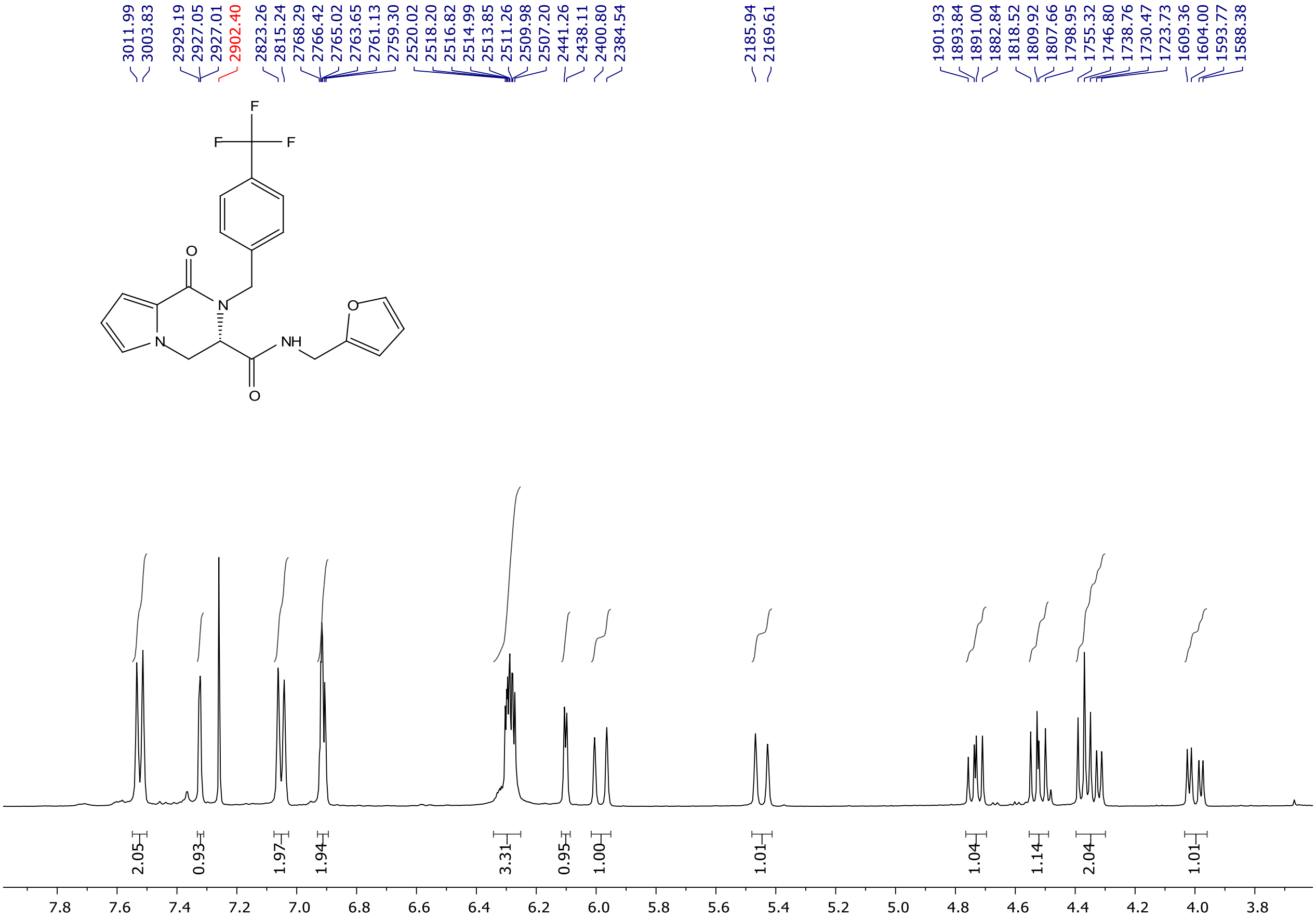

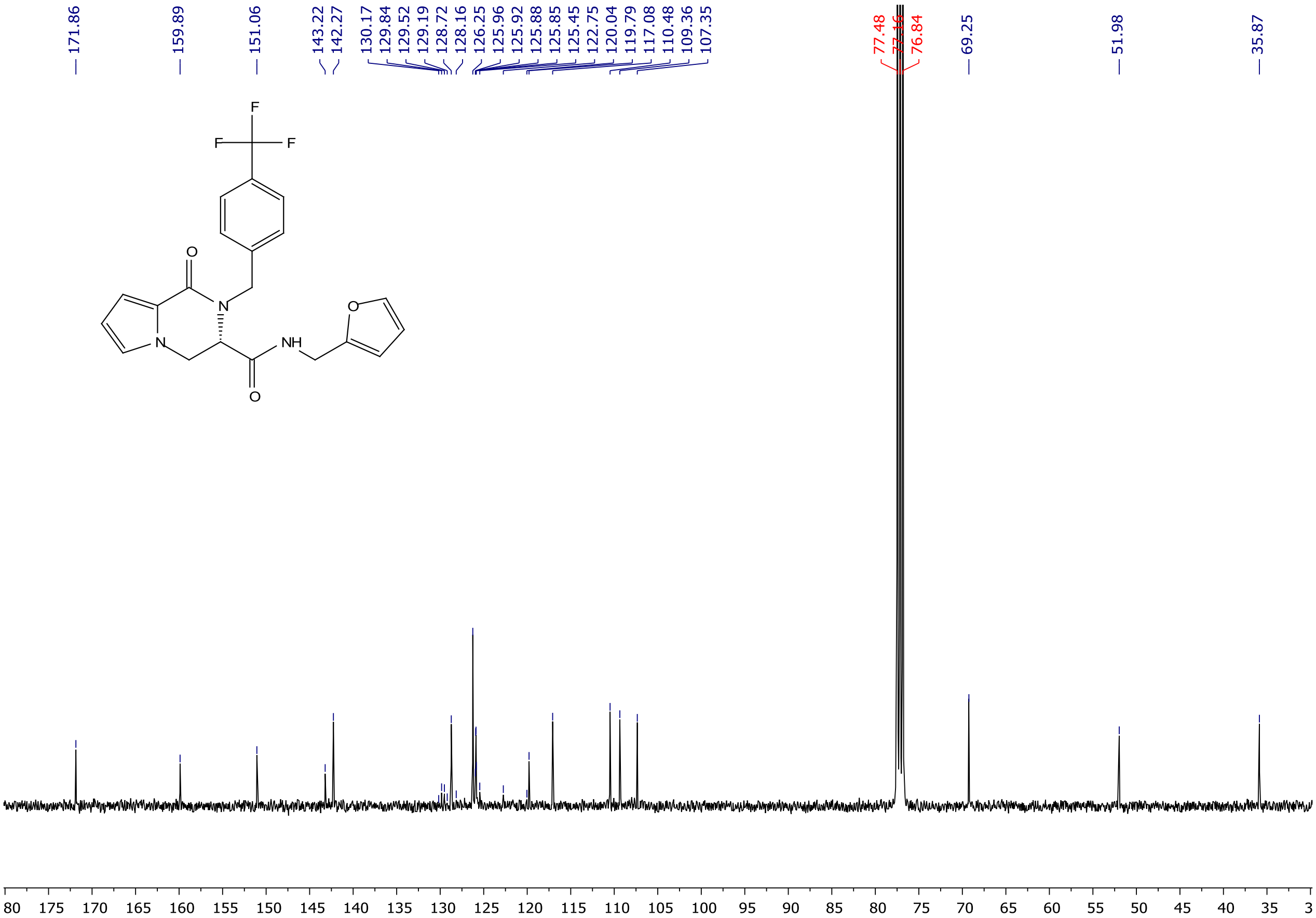

^{1}H and ^{13}C NMR: *(S)-N-((5-Methylfuran-2-yl)methyl)-1-2-(4-(trifluoromethyl)benzyl)-oxo-1,2,3,4-tetrahydropyrrolo[1,2-a]pyrazine-3-carboxamide* (***D4***)

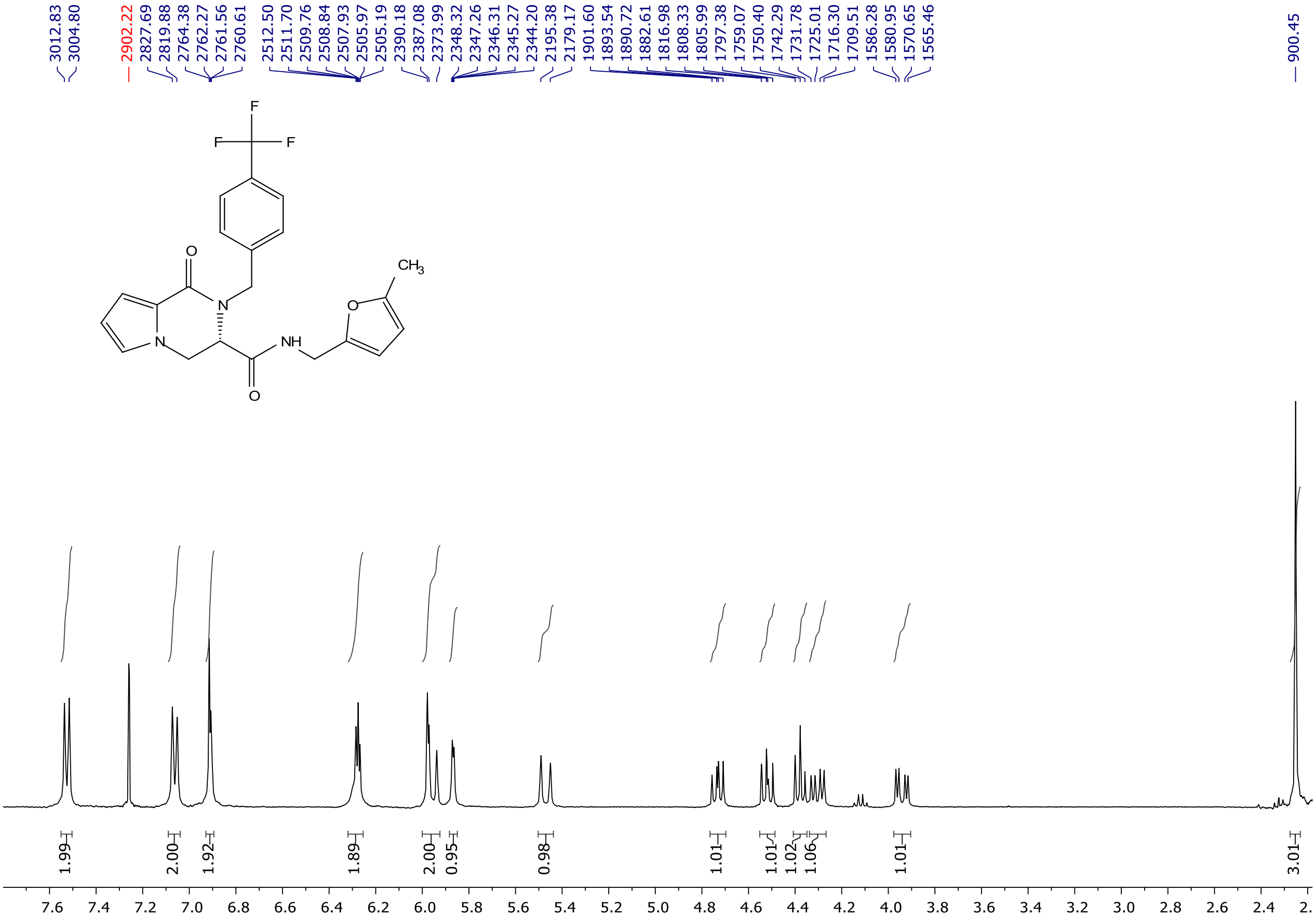

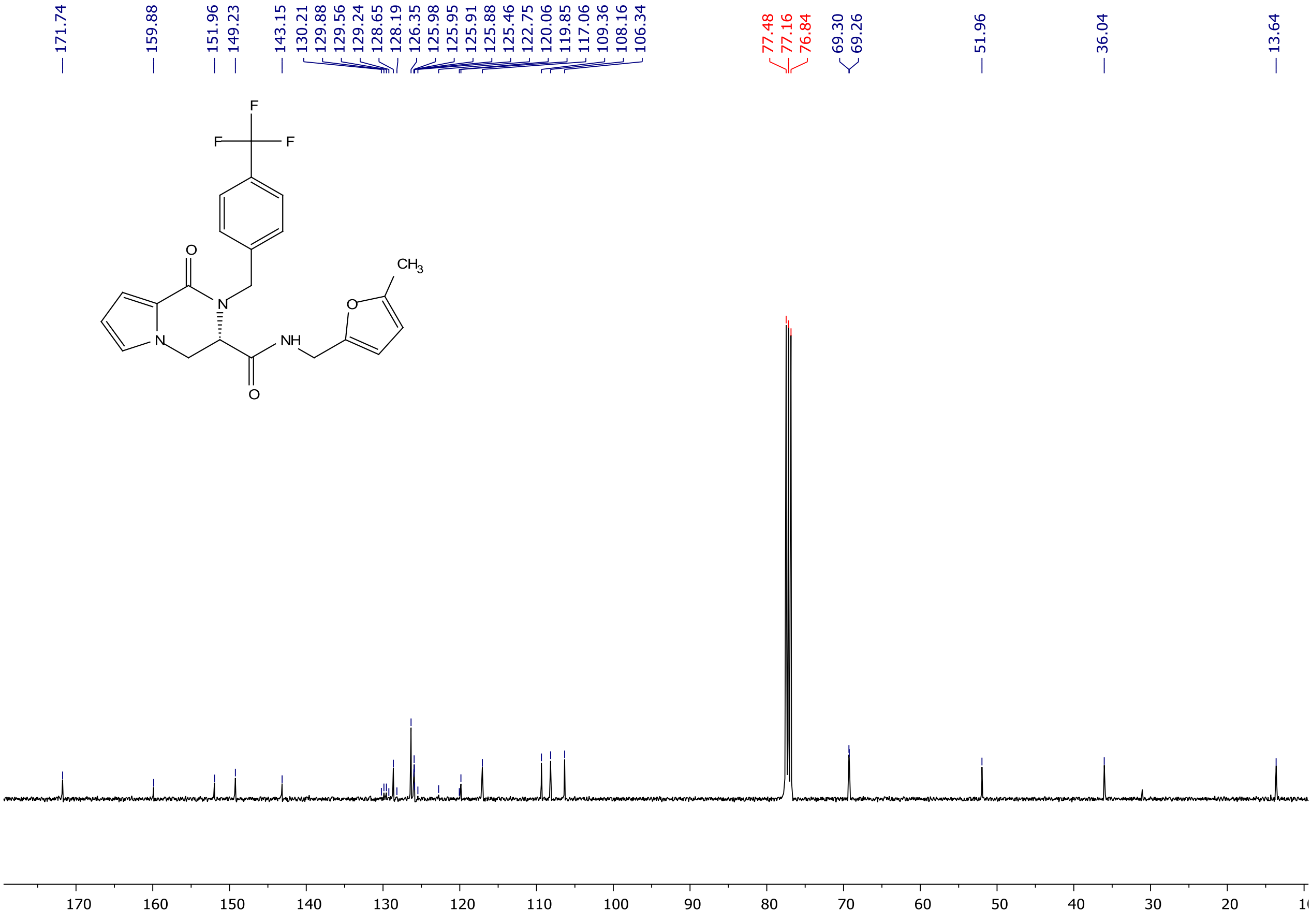

^{1}H and ^{13}C NMR: *(S)-N-(Thiophen-2-ylmethyl)-1-oxo-2-(4-(trifluoromethyl)benzyl)-1,2,3,4-tetrahydropyrrolo[1,2-a]pyrazine-3-carboxamide* (***D5***)

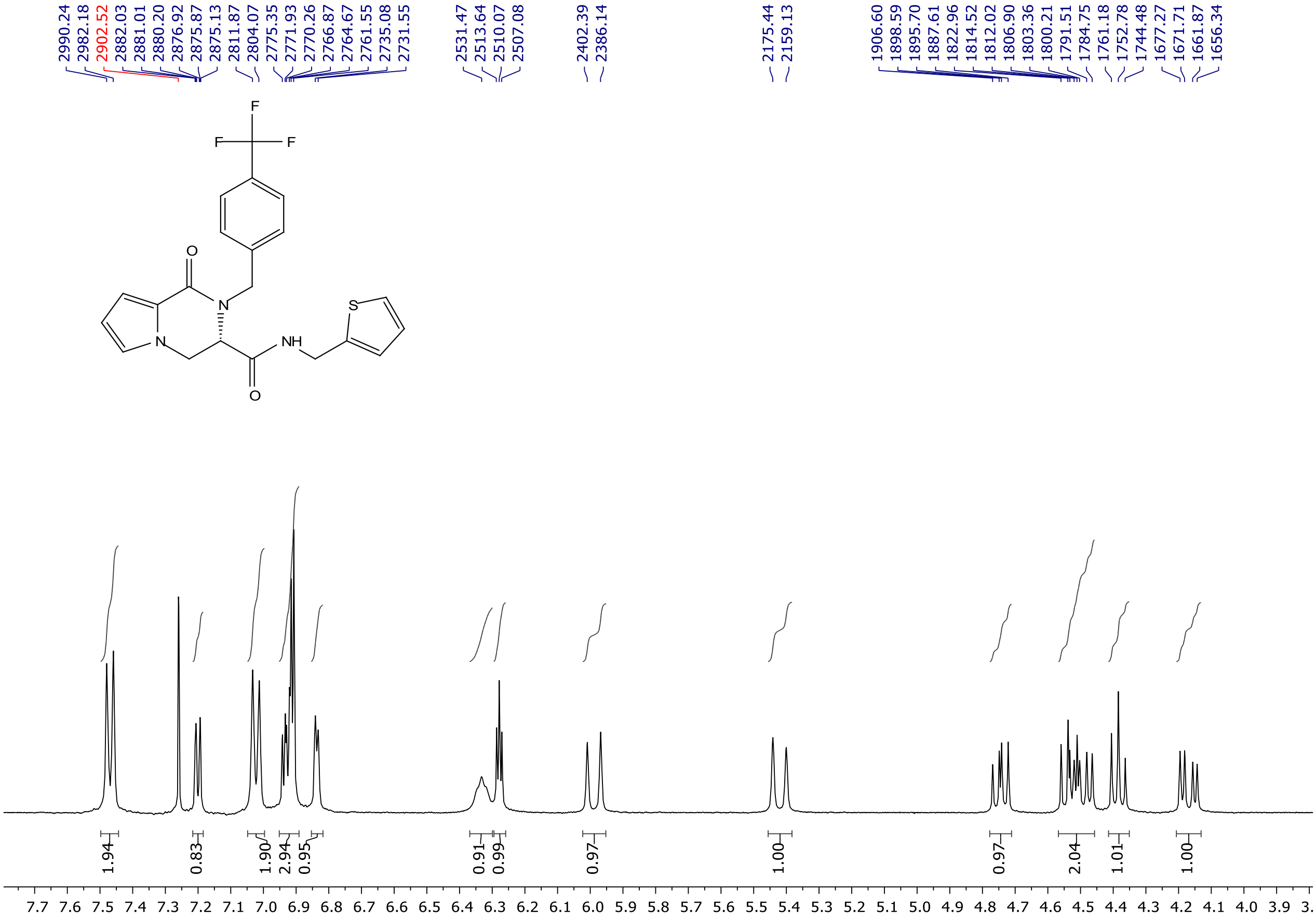

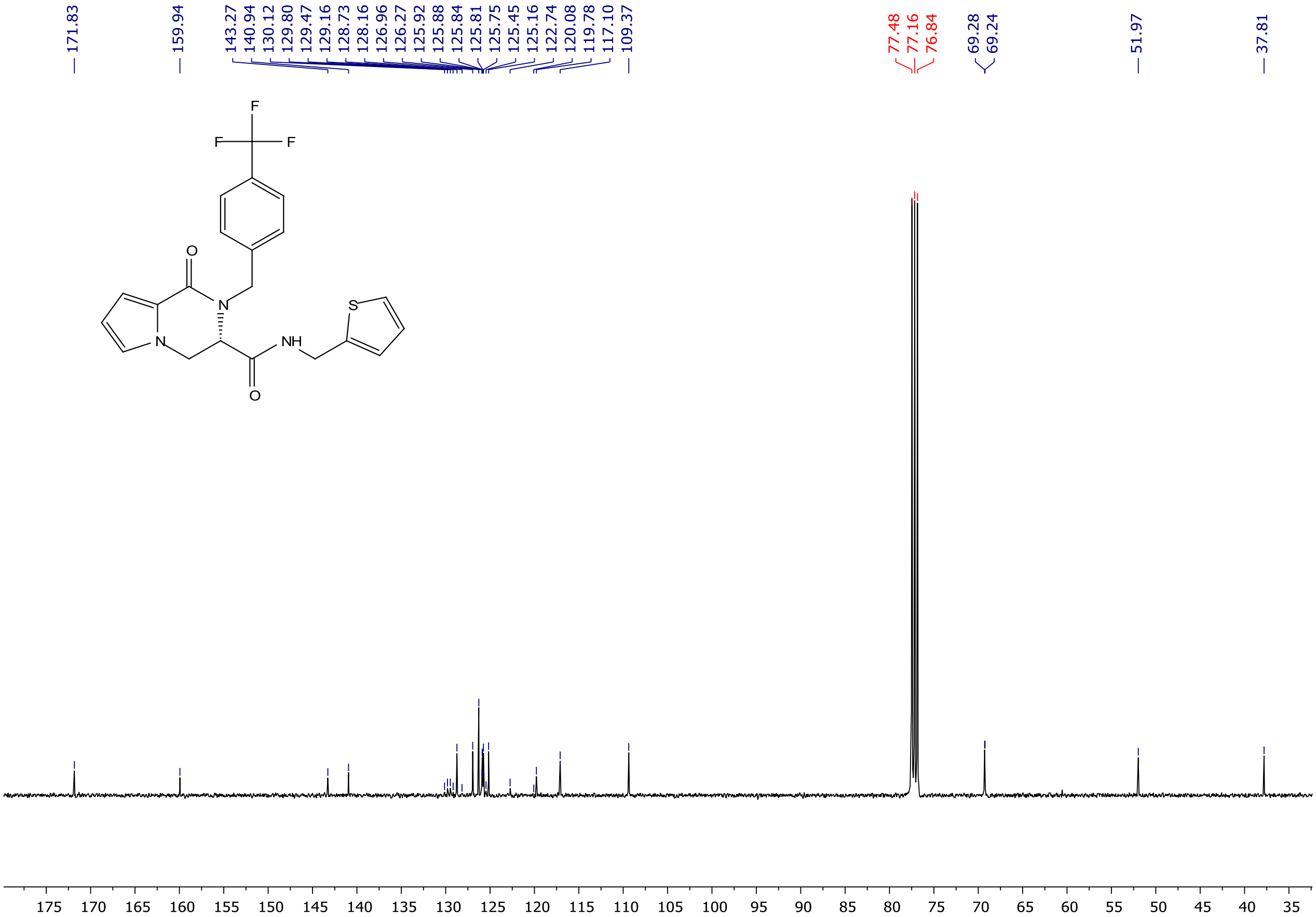

^{1}H and ^{13}C NMR: *(S)-N-Benzyl-1-oxo-2-(4-(trifluoromethoxy)benzyl)-1,2,3,4-tetrahydropyrrolo[1,2-a]pyrazine-3-carboxamide* (***E1***)

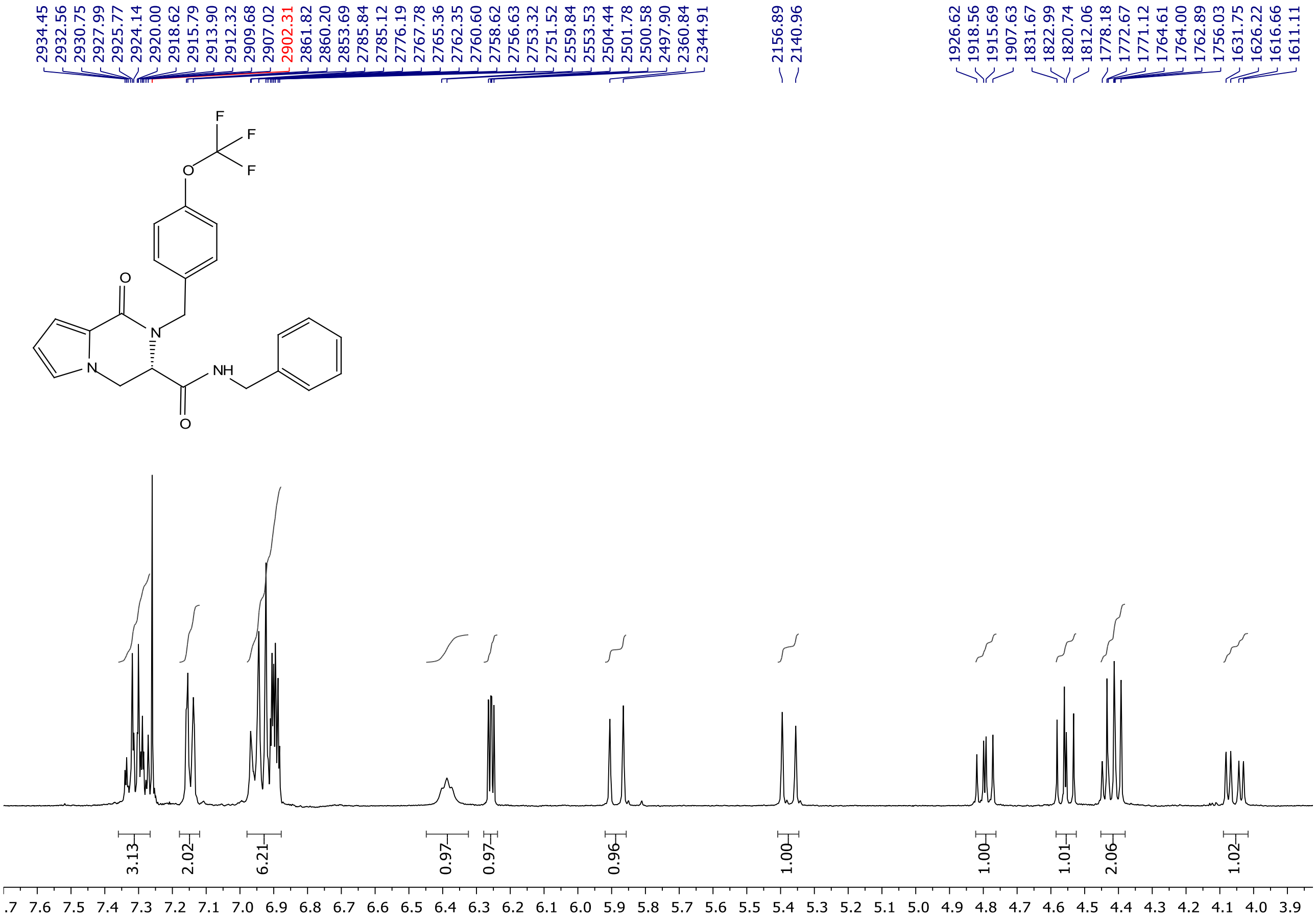

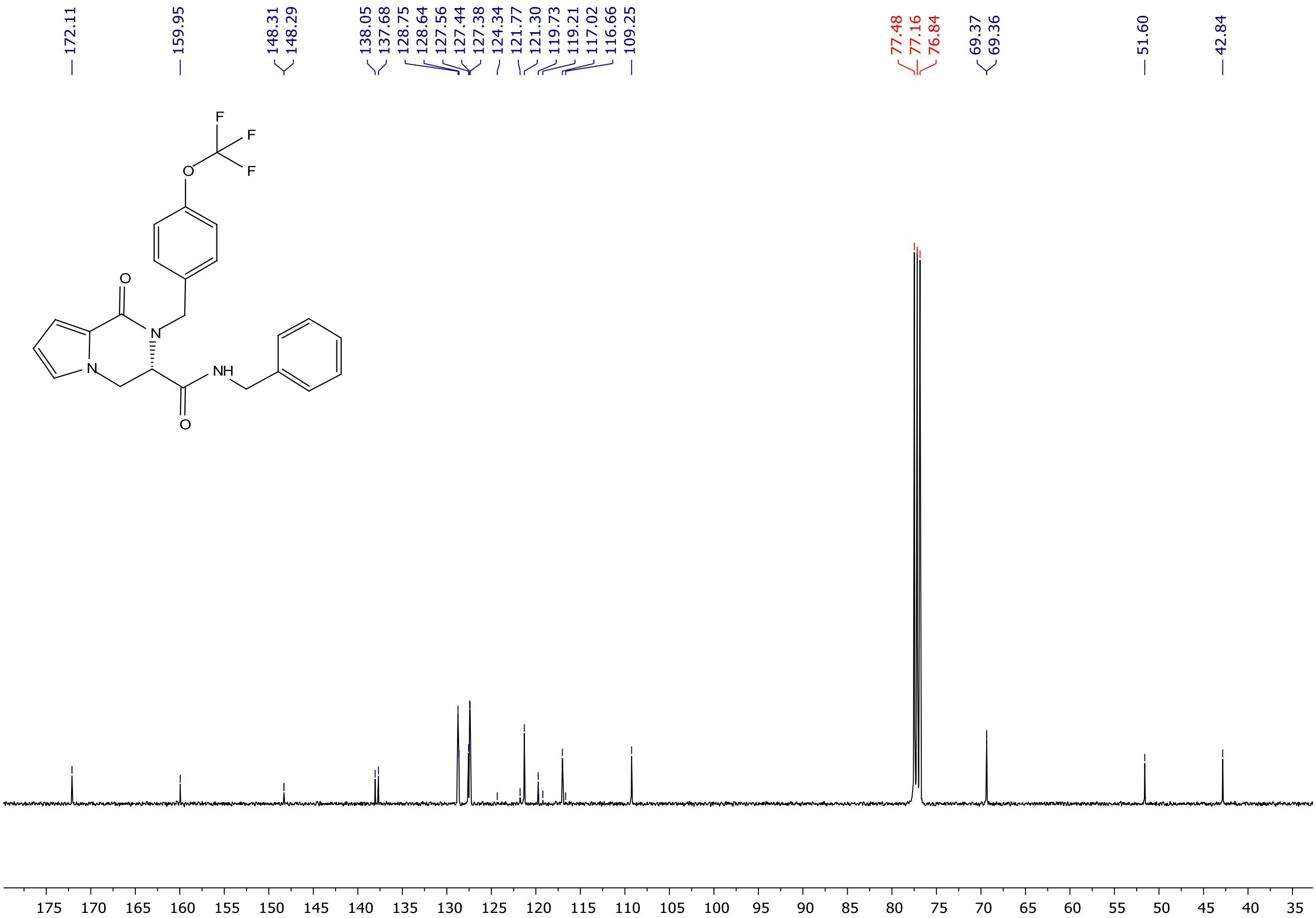

^{1}H and ^{13}C NMR: *(S)-N-(3-Chlorobenzyl)-1-oxo-2-(4-(trifluoromethoxy)benzyl)-1,2,3,4-tetrahydropyrrolo[1,2-a]pyrazine-3-carboxamide (**E2**)*

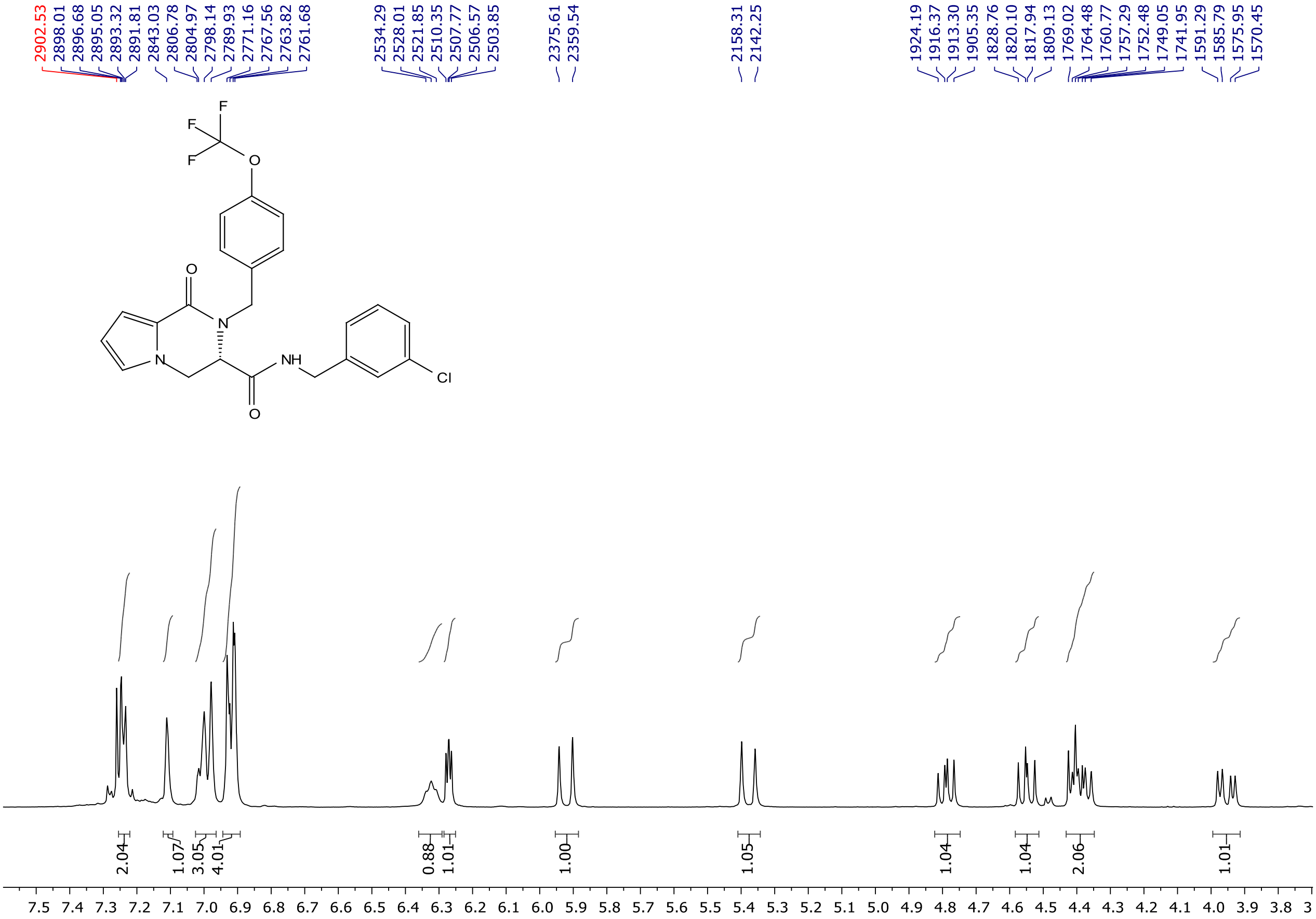

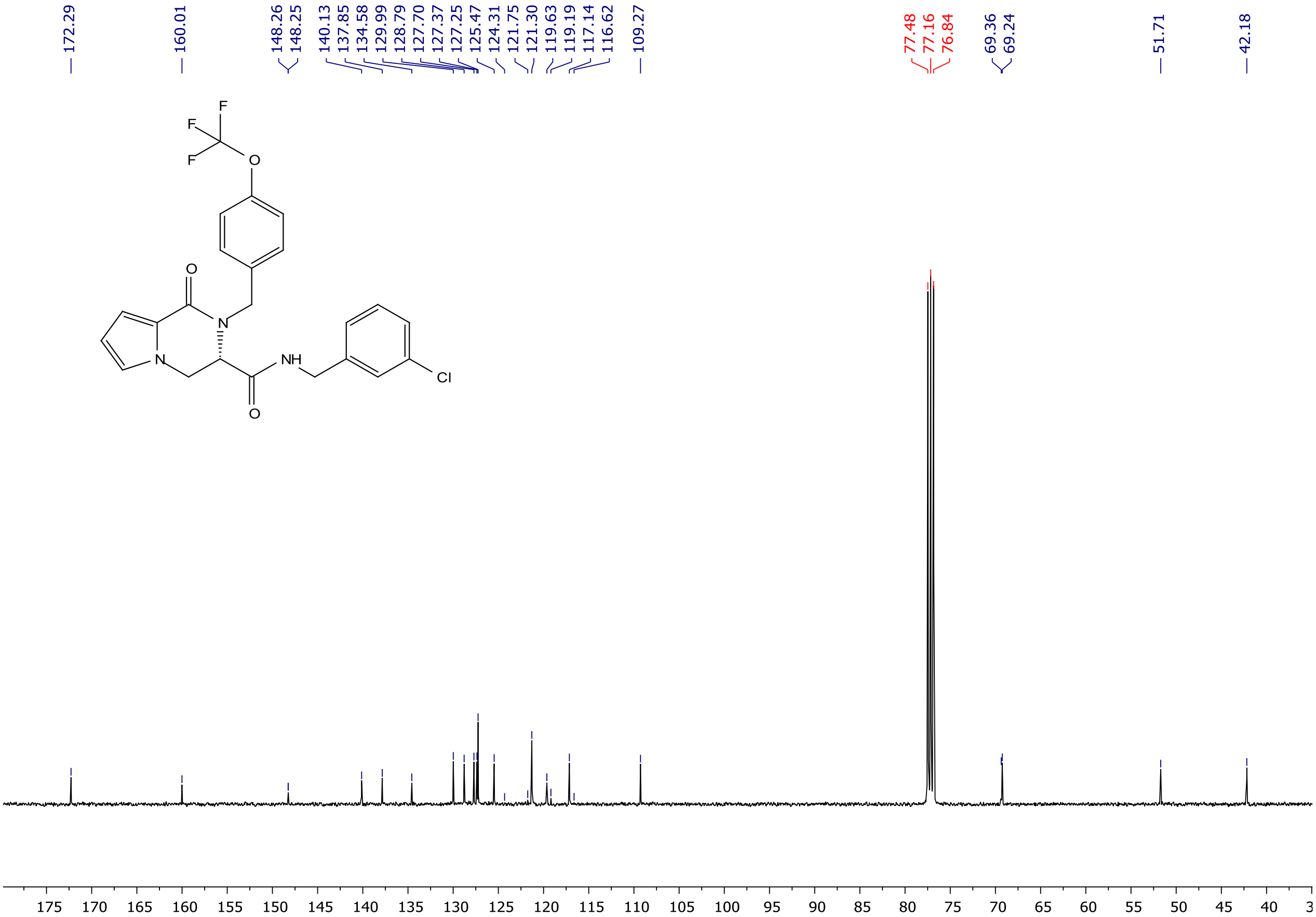

^{1}H and ^{13}C NMR: *(S)-N-(Furan-2-ylmethyl)-1-oxo-2-(4-(trifluoromethoxy)benzyl)-1,2,3,4-tetrahydropyrrolo[1,2-a]pyrazine-3-carboxamide (**E3**)*

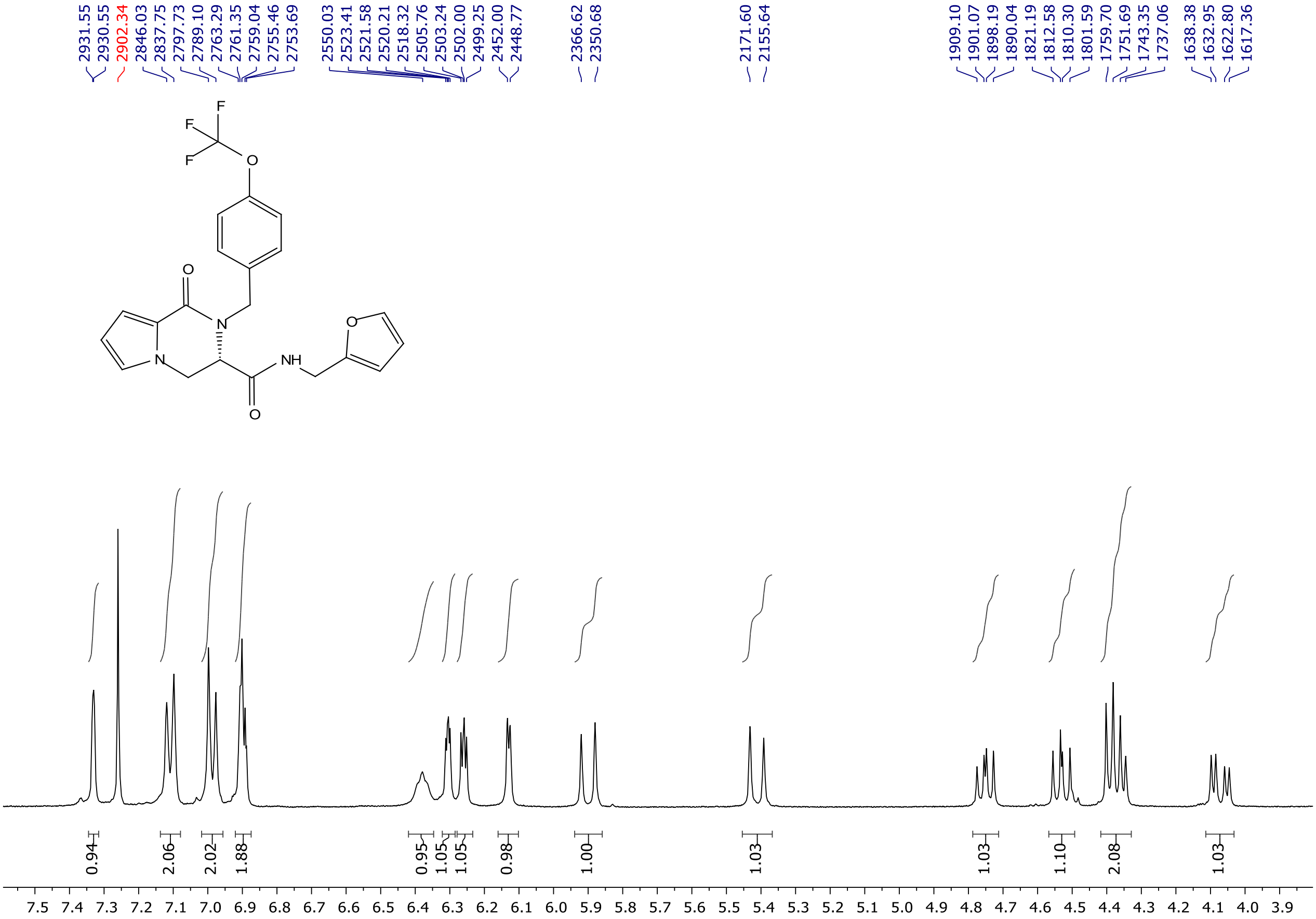

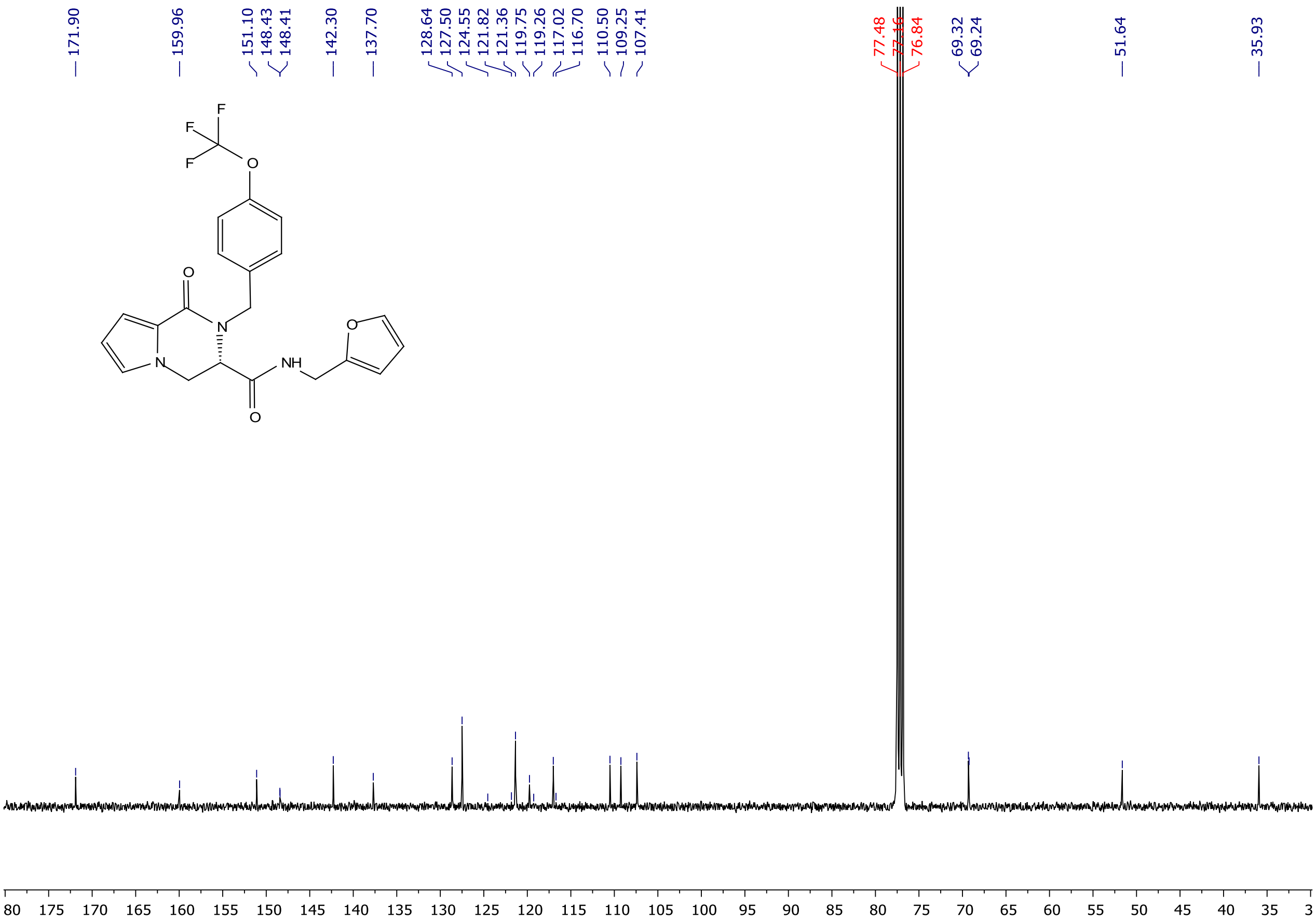

^{1}H and ^{13}C NMR: *(S)-N-((5-Methylfuran-2-yl)methyl)-1-oxo-2-(4-(trifluoromethoxy)benzyl)-1,2,3,4-tetrahydropyrrolo[1,2-a]pyrazine-3-carboxamide* (***E4***)

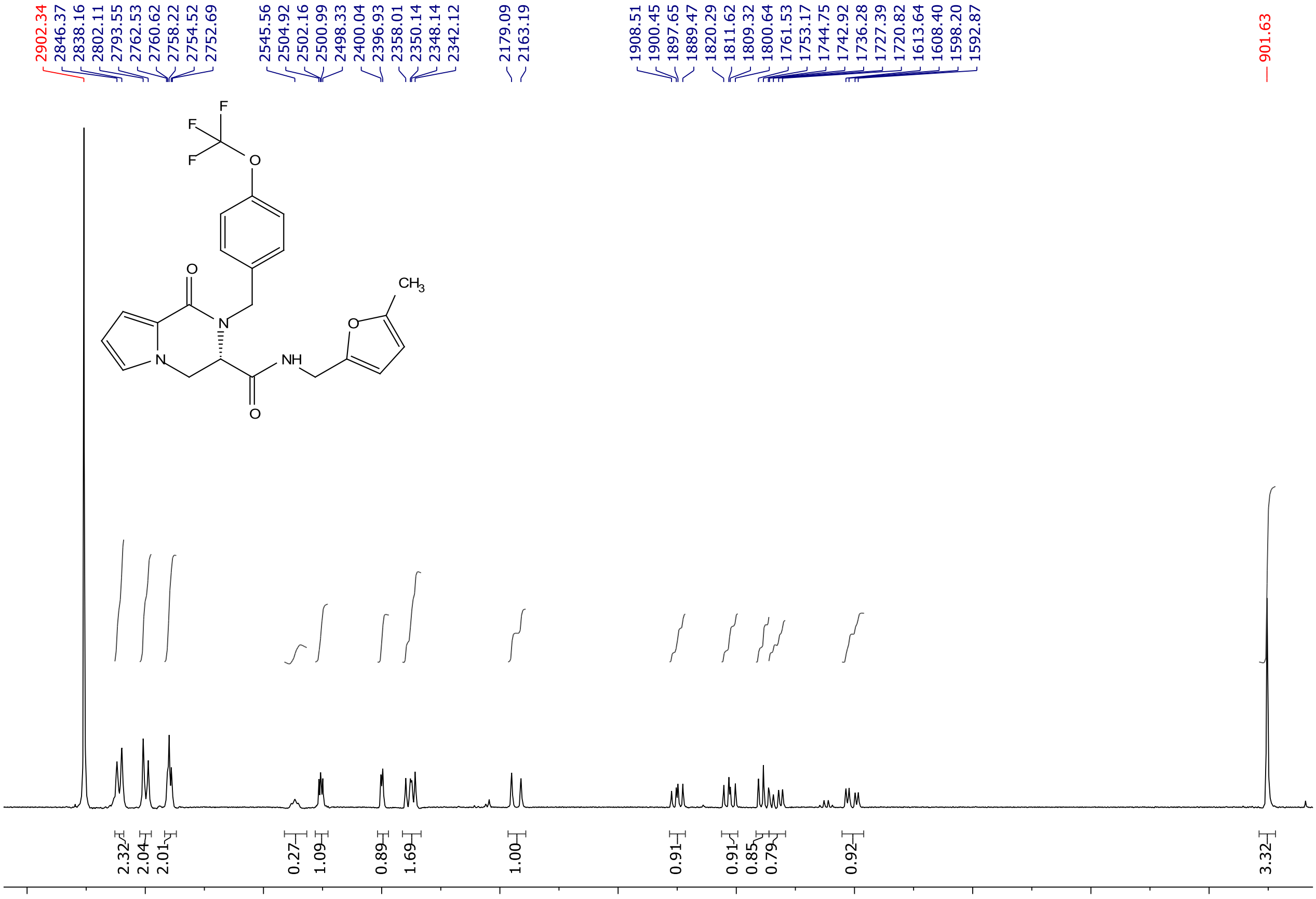

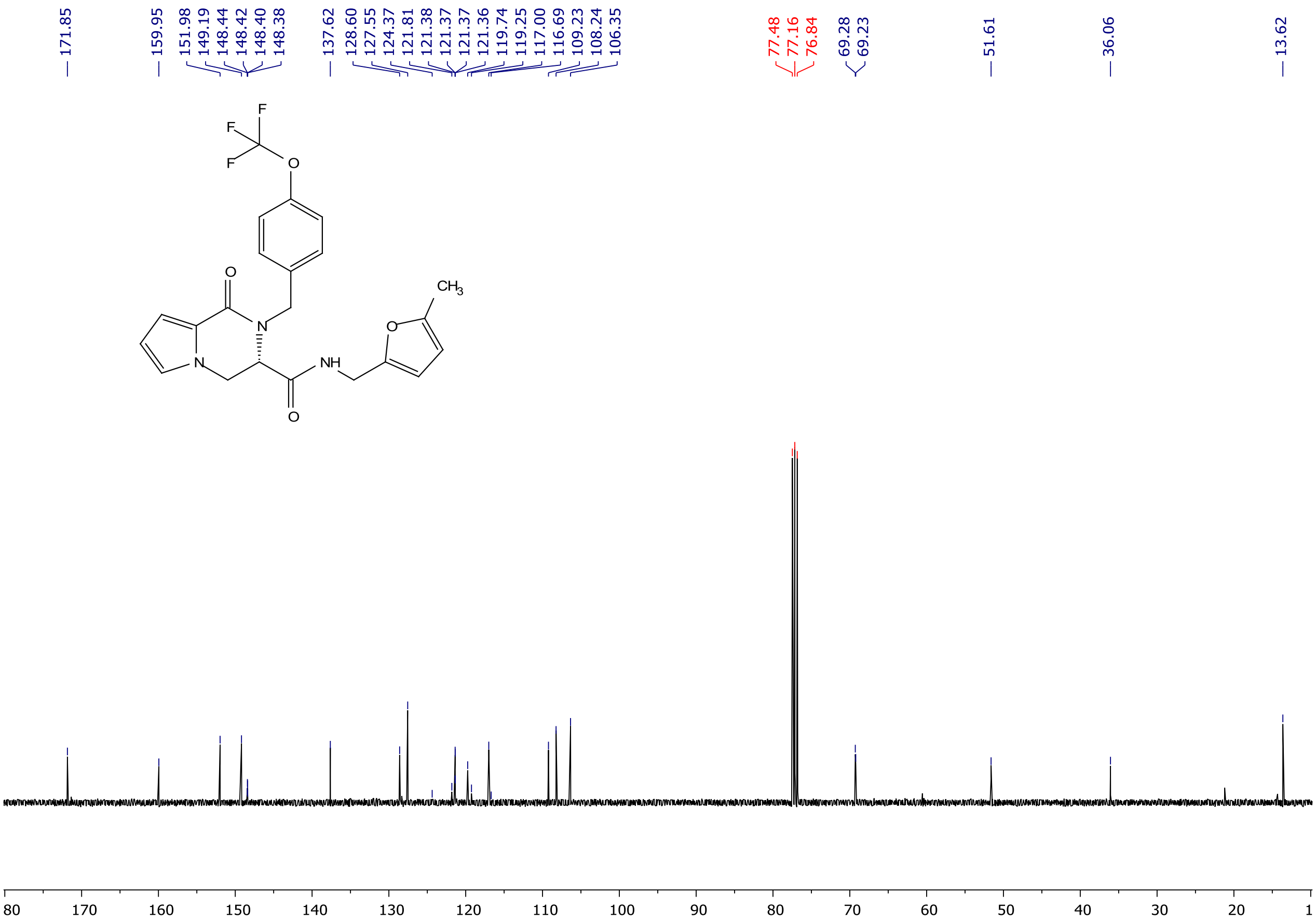

^{1}H and ^{13}C NMR: *(S)-N-(Thiophen-2-ylmethyl)-1-oxo-2-(4-(trifluoromethoxy)benzyl)-1,2,3,4-tetrahydropyrrolo[1,2-a]pyrazine-3-carboxamide (**E5**)*

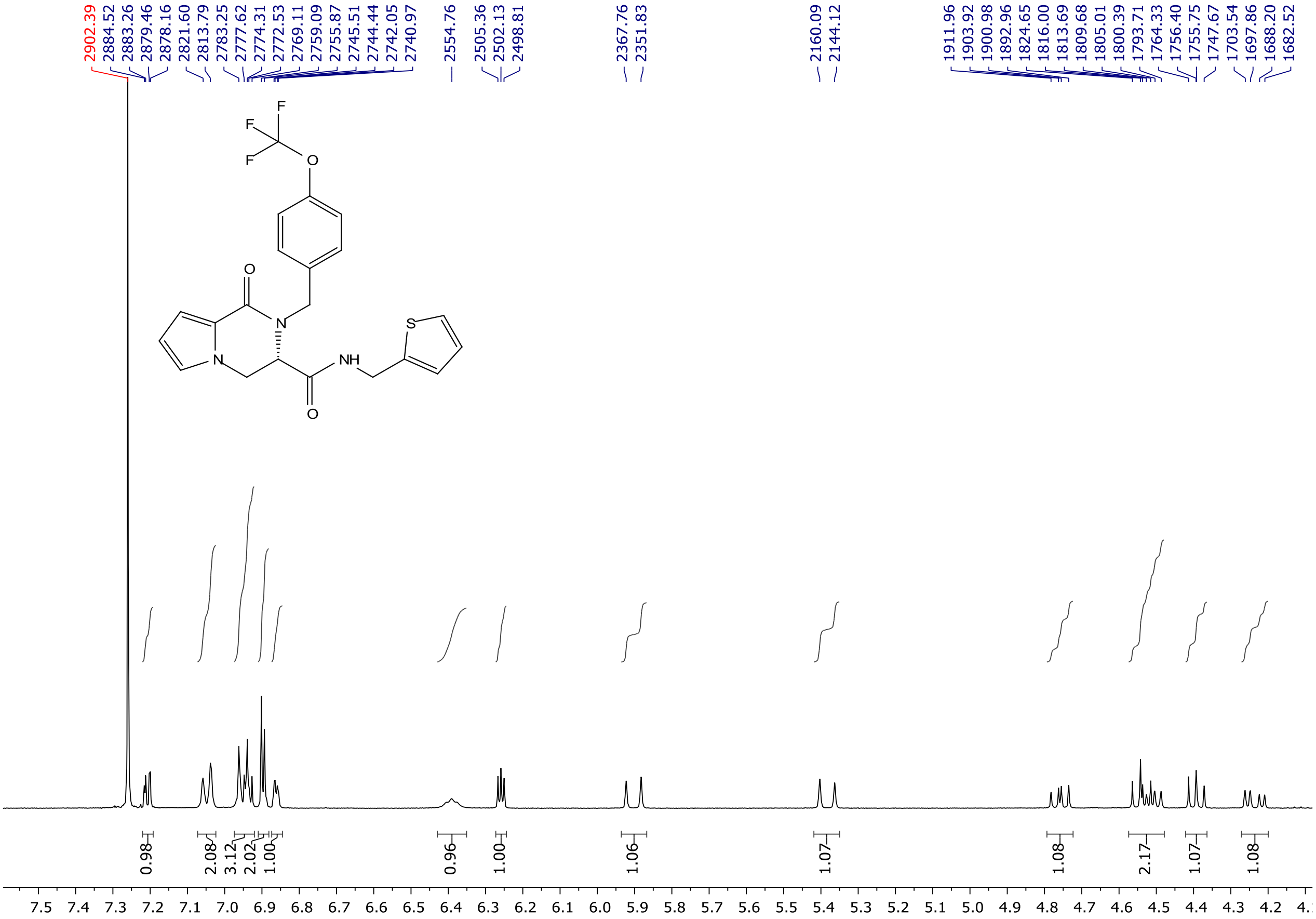

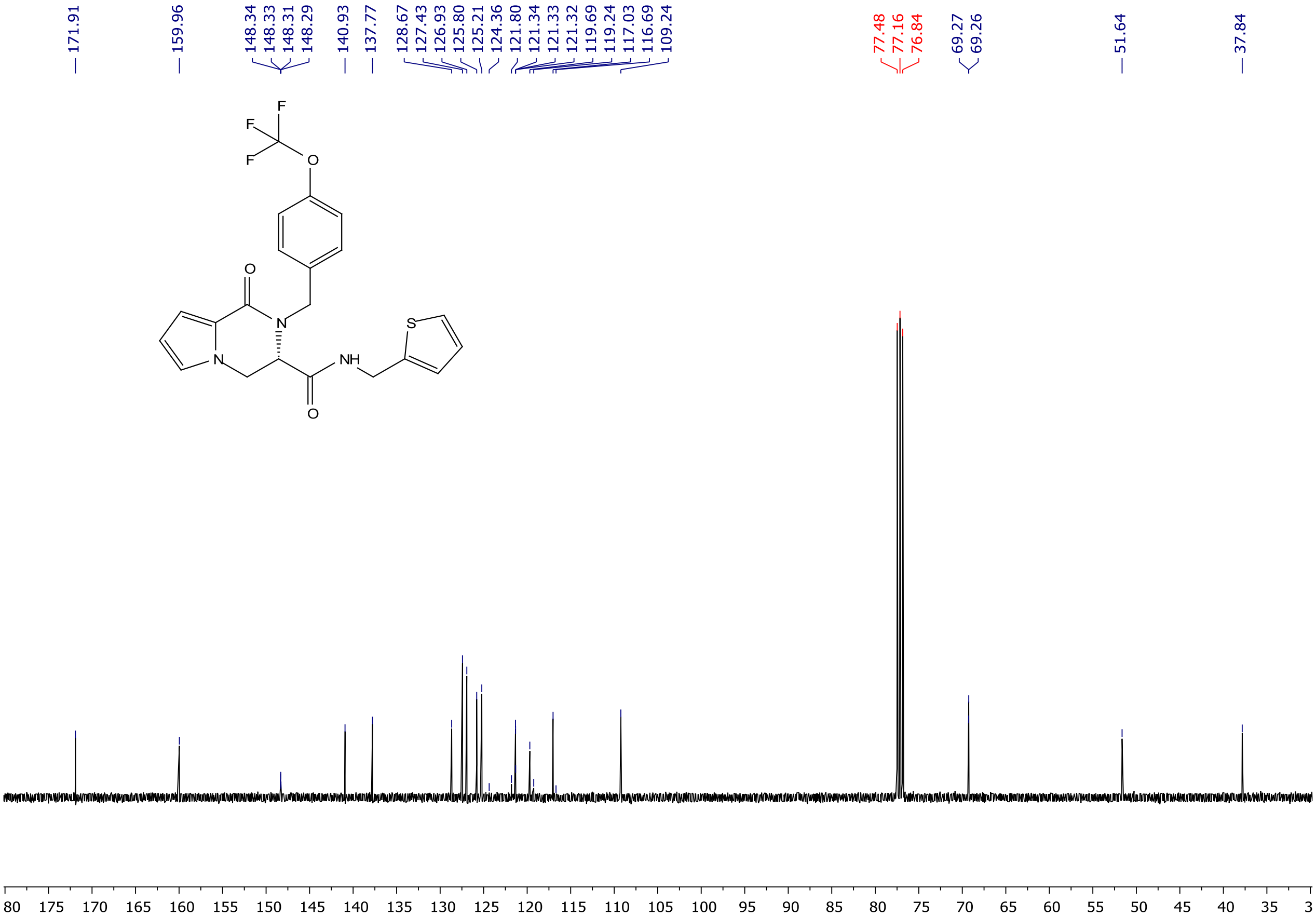

^{1}H and ^{13}C NMR: *(S)-N-Benzyl-2-(4-nitrobenzyl)-1-oxo-1,2,3,4-tetrahydropyrrolo[1,2-a]pyrazine-3-carboxamide (**F1**)*

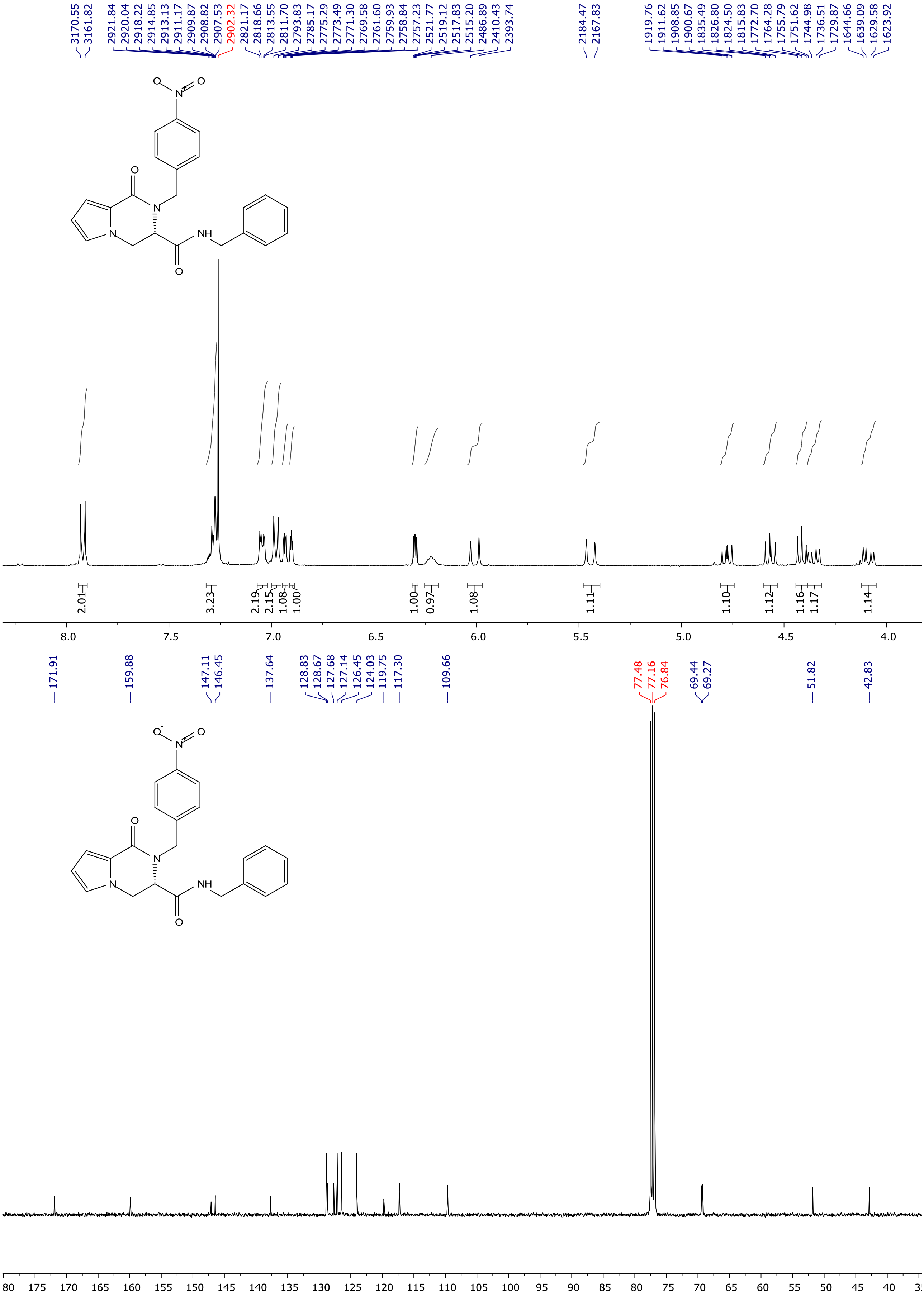

^{1}H and ^{13}C NMR: *(S)-N-(3-Chlorobenzyl)-2-(4-nitrobenzyl)-1-oxo-1,2,3,4-tetrahydropyrrolo[1,2-a]pyrazine-3-carboxamide* (***F2***)

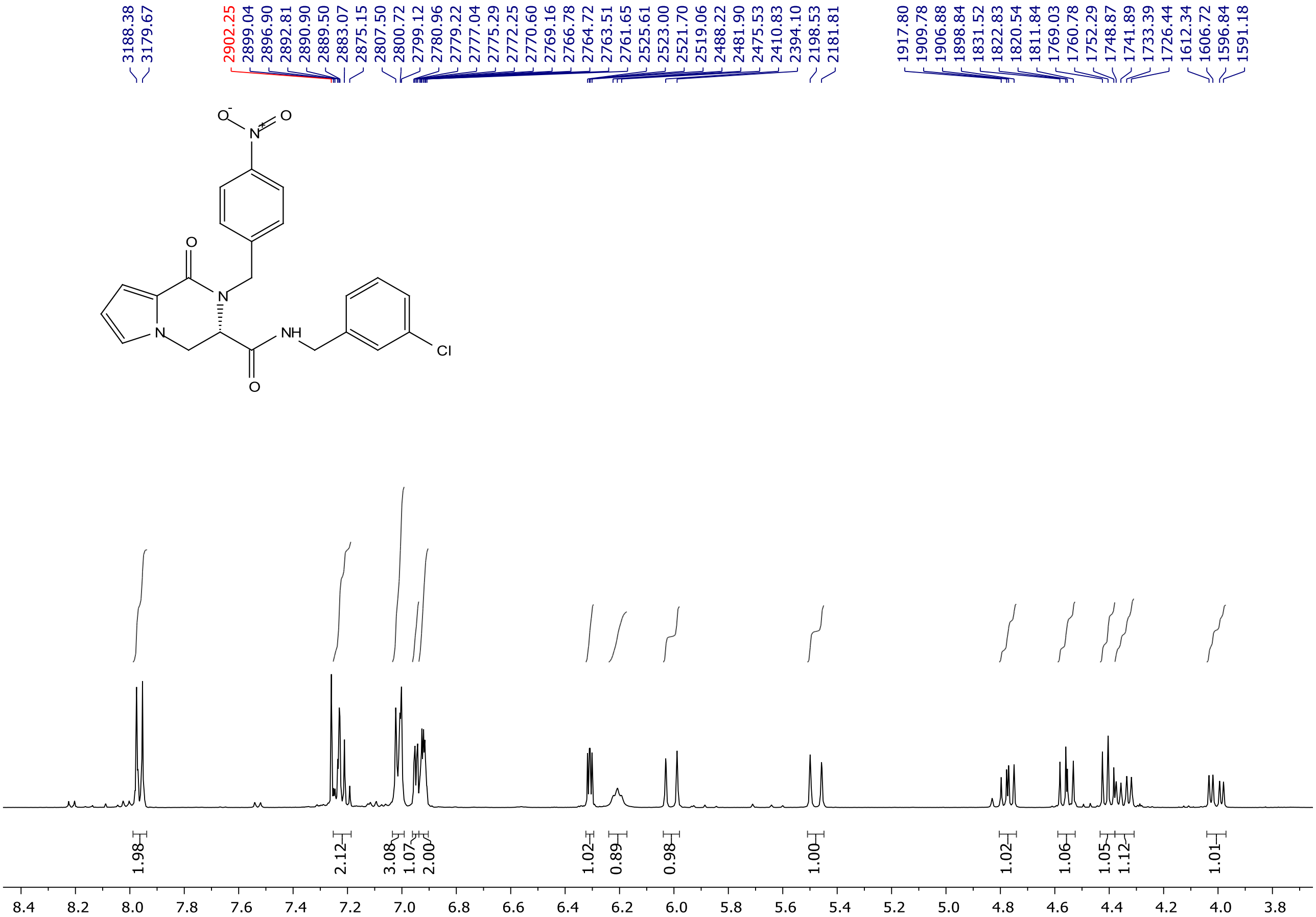

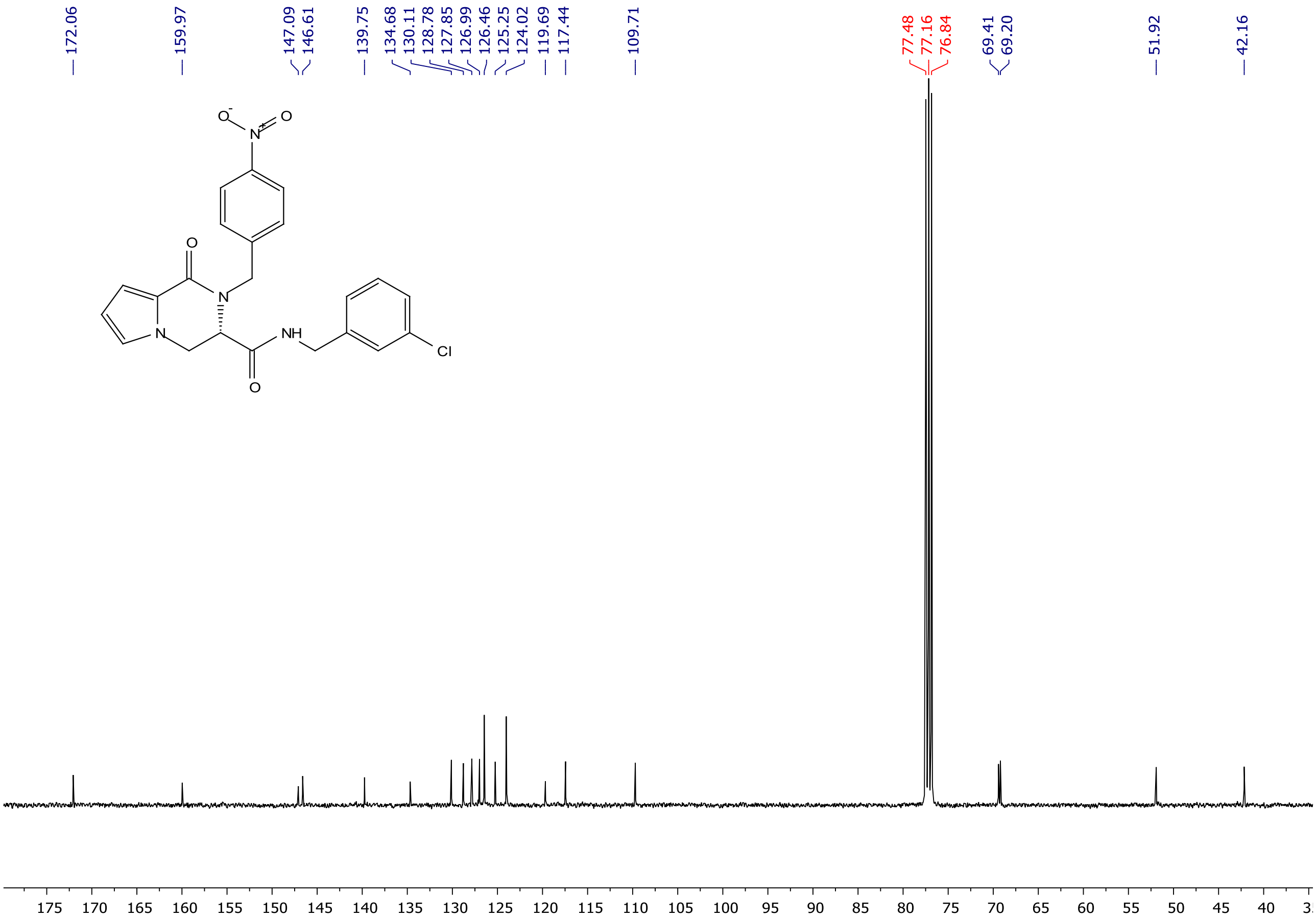

^{1}H and ^{13}C NMR: *(S)-N-(Furan-2-ylmethyl)-2-(4-nitrobenzyl)-1-oxo-1,2,3,4-tetrahydropyrrolo[1,2-a]pyrazine-3-carboxamide* (***F3***)

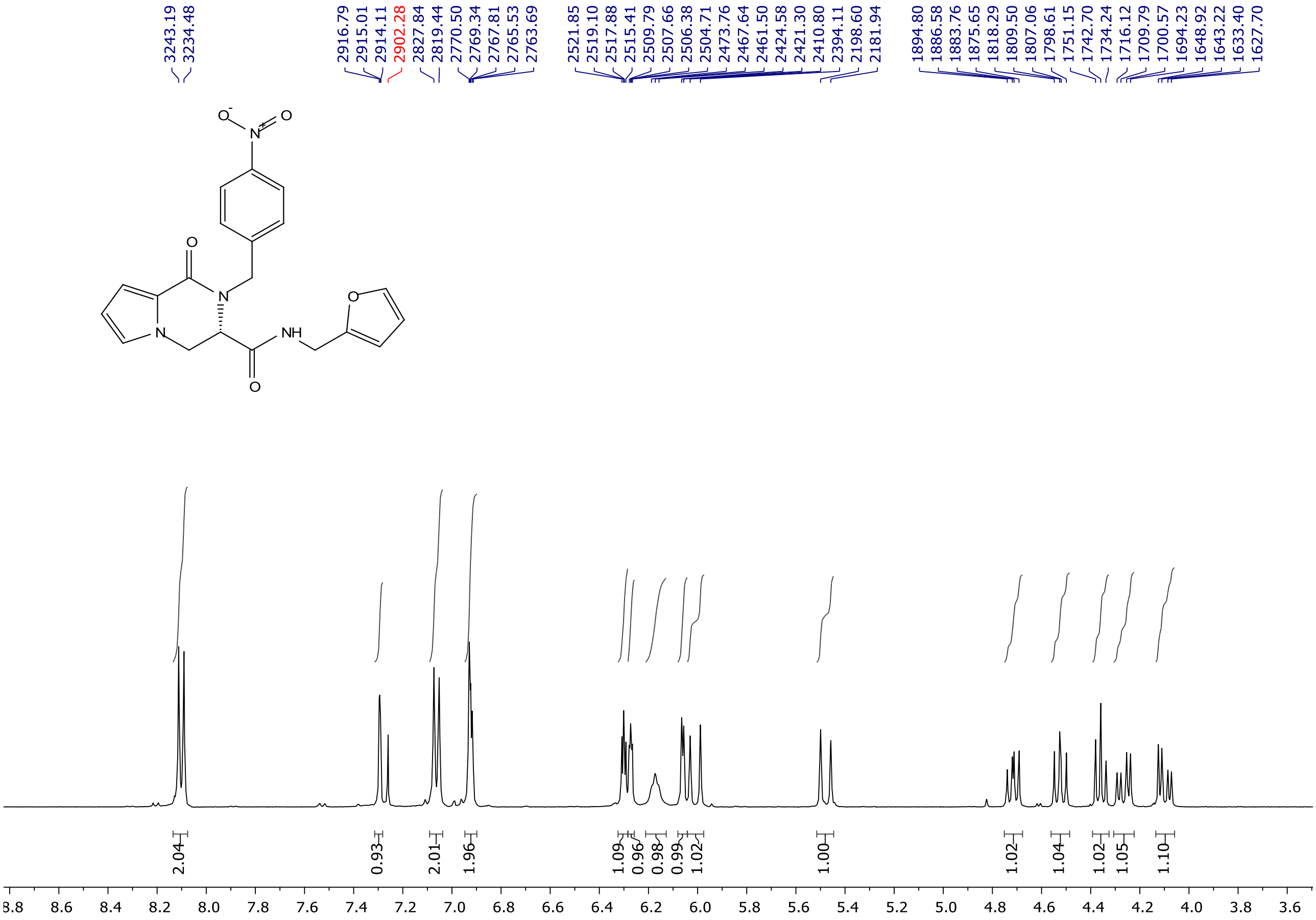

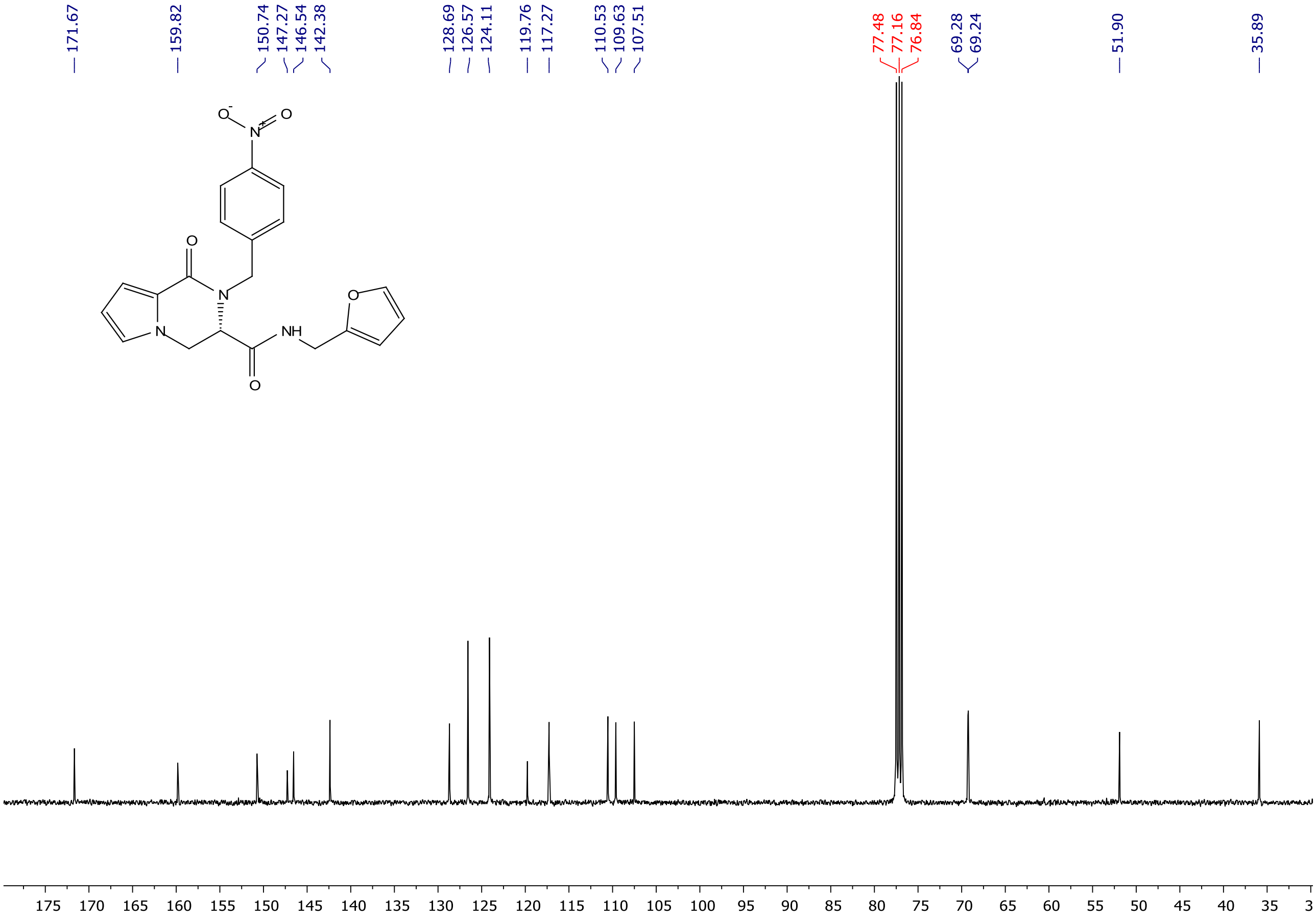

^{1}H and ^{13}C NMR: *(S)-N-((5-Methylfuran-2-yl)methyl)-2-(4-nitrobenzyl)-1-oxo-1,2,3,4-tetrahydropyrrolo[1,2-a]pyrazine-3-carboxamide* (***F4***)

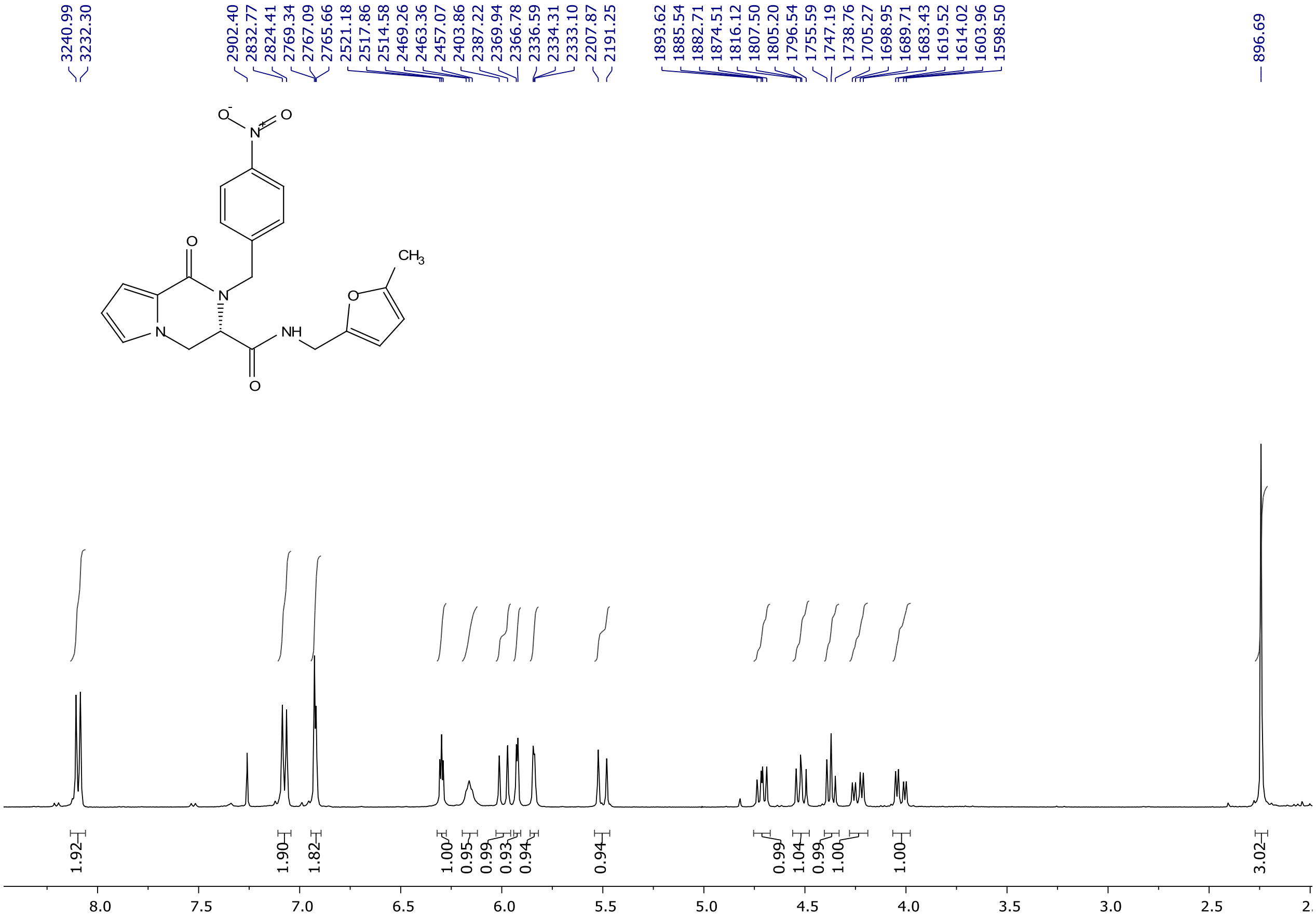

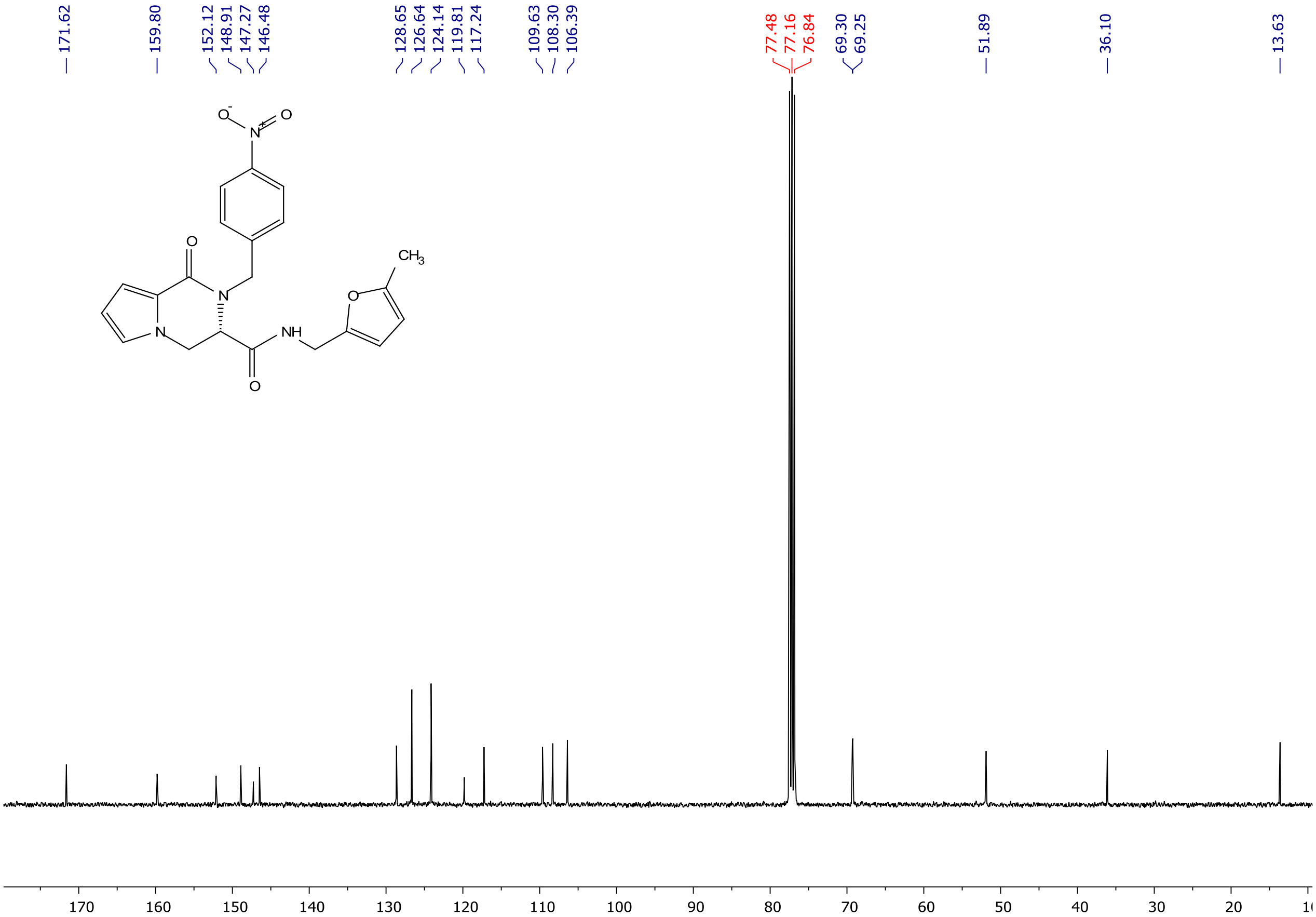

^{1}H and ^{13}C NMR: *(S)-N-(Thiophen-2-ylmethyl)-2-(4-nitrobenzyl)-1-oxo-1,2,3,4-tetrahydropyrrolo[1,2-a]pyrazine-3-carboxamide* ***(F5)***

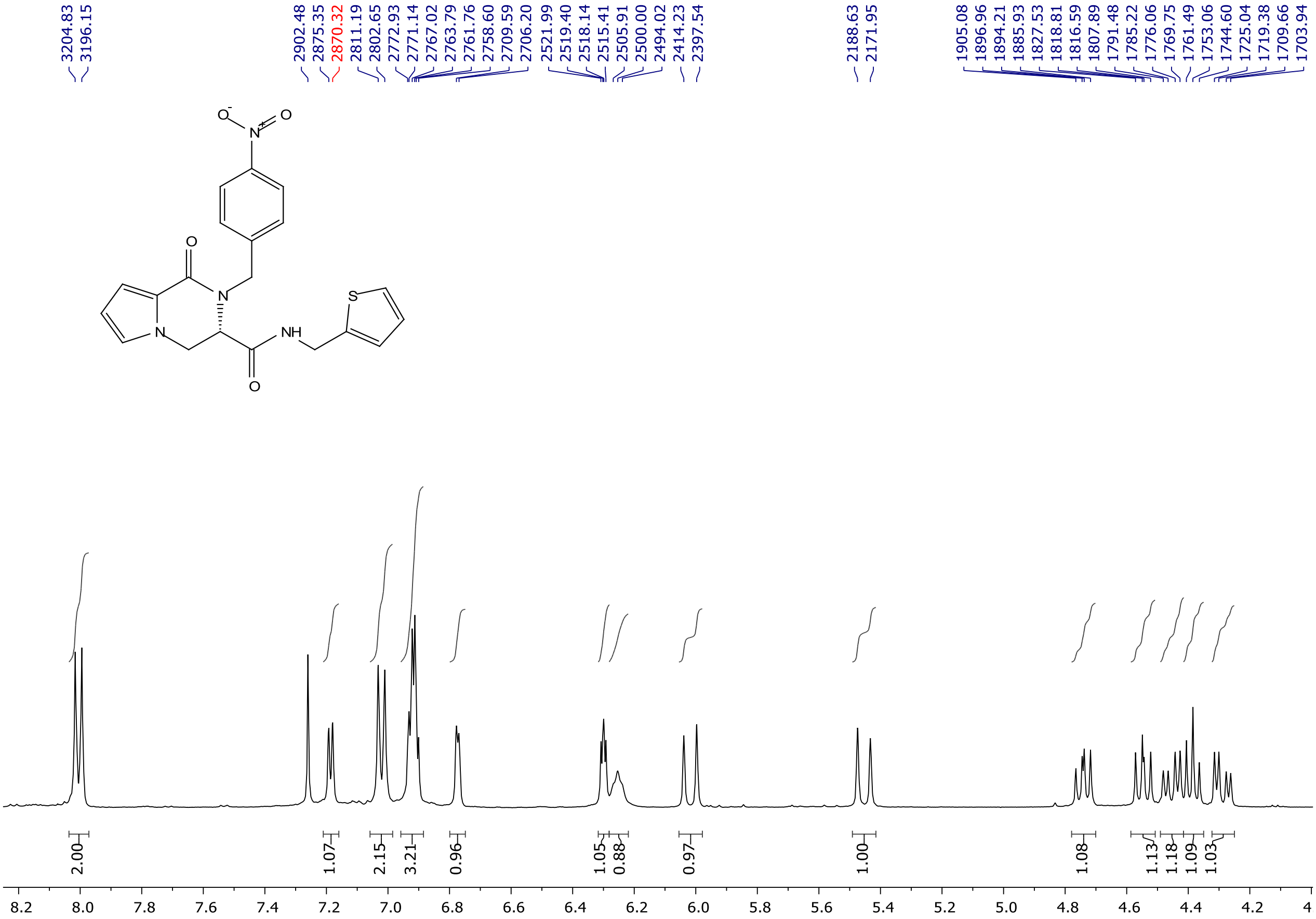

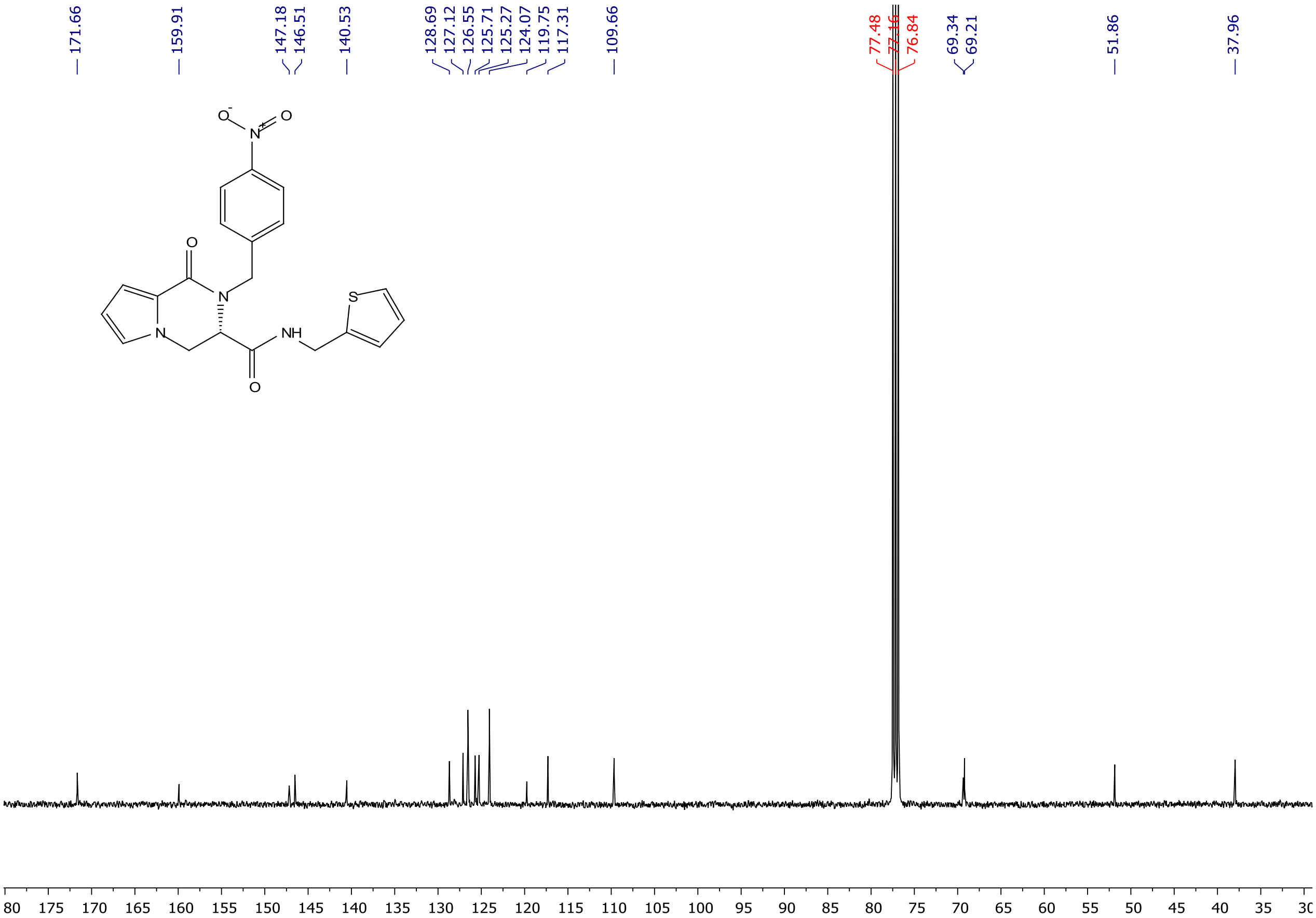

2. Raw data of Western blot for Fig. 4.

4a)

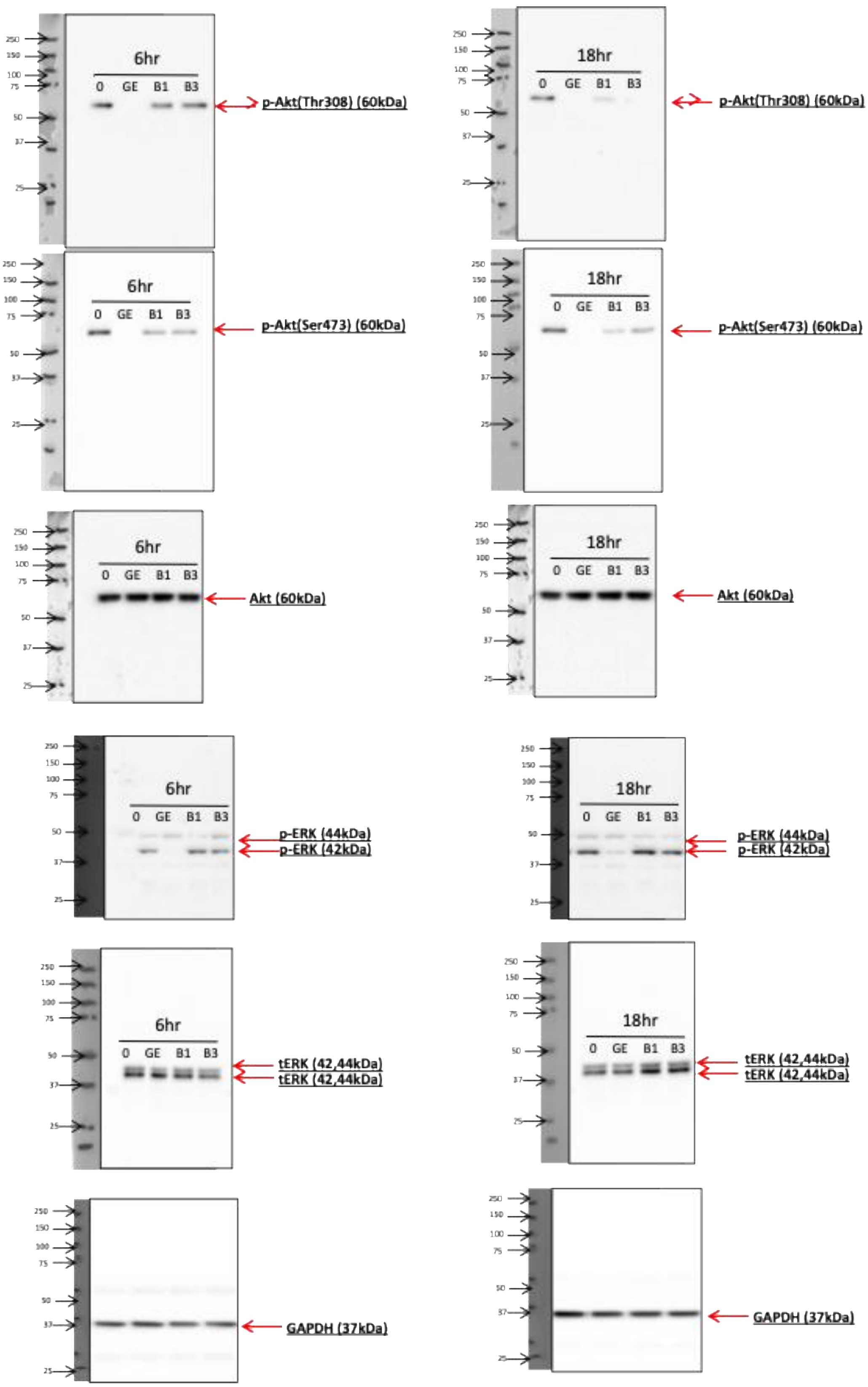

4b)

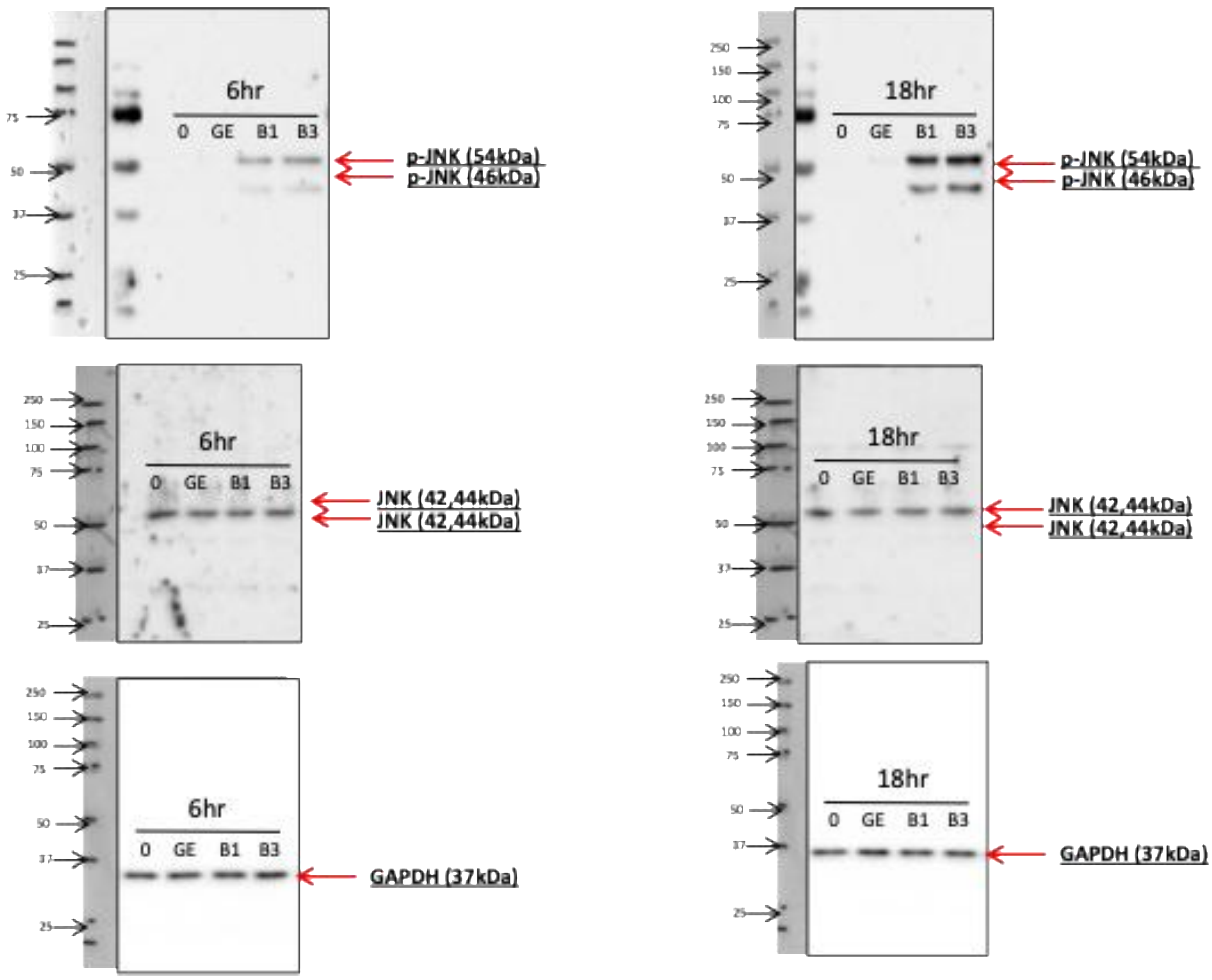
6hr
0 GE B1 B3
p-JNK (54kDa)
p-JNK (46kDa)
18hr
0 GE B1 B3
p-JNK (54kDa)
p-JNK (46kDa)
6hr
0 GE B1 B3
JNK (42,44kDa)
JNK (42,44kDa)
18hr
0 GE B1 B3
JNK (42,44kDa)
JNK (42,44kDa)
6hr
0 GE B1 B3
GAPDH (37kDa)
18hr
0 GE B1 B3
GAPDH (37kDa)
250
150
100
75
50
37
25
